# Genome sequencing of the extinct Eurasian wild aurochs, *Bos primigenius*, illuminates the phylogeography and evolution of cattle

**DOI:** 10.1186/s13059-015-0790-2

**Published:** 2015-10-26

**Authors:** Stephen D E Park, David A. Magee, Paul A. McGettigan, Matthew D. Teasdale, Ceiridwen J. Edwards, Amanda J. Lohan, Alison Murphy, Martin Braud, Mark T. Donoghue, Yuan Liu, Andrew T. Chamberlain, Kévin Rue-Albrecht, Steven Schroeder, Charles Spillane, Shuaishuai Tai, Daniel G. Bradley, Tad S. Sonstegard, Brendan J. Loftus, David E. MacHugh

**Affiliations:** IdentiGEN Ltd, Unit 2, Trinity Enterprise Centre, Pearse Street, Dublin 2, Ireland; Animal Genomics Laboratory, UCD School of Agriculture and Food Science, University College Dublin, Belfield, Dublin 4, Ireland; Department of Animal Science, University of Connecticut, Storrs, CT 06029 USA; Smurfit Institute of Genetics, Trinity College, Dublin 2, Ireland; Research Laboratory for Archaeology and the History of Art, Dyson Perrins Building, South Parks Rd, Oxford, OX1 3QY UK; UCD Conway Institute of Biomolecular and Biomedical Research, University College Dublin, Dublin 4, Ireland; Genetics and Biotechnology Laboratory, Plant and AgriBiosciences Research Centre (PABC), School of Natural Sciences, National University of Ireland Galway, University Road, Galway, Ireland; BGI Shenzhen, Beishan Industrial Zone, Yantian District, Shenzhen, 518083 China; Faculty of Life Sciences, University of Manchester, Oxford Road, Manchester, M13 9PT UK; Animal Genomics and Improvement Laboratory, Agricultural Research Service, USDA, Beltsville, MD 20705-2350 USA; Recombinetics Inc., St. Paul, MN 55104 USA; UCD School of Medicine, University College Dublin, Belfield, Dublin 4, Ireland

**Keywords:** Ancient DNA, Aurochs, *Bos primigenius*, Cattle, Domestication, Evolution, Genome, Hybridization

## Abstract

**Background:**

Domestication of the now-extinct wild aurochs, *Bos primigenius*, gave rise to the two major domestic extant cattle taxa, *B. taurus* and *B. indicus*. While previous genetic studies have shed some light on the evolutionary relationships between European aurochs and modern cattle, important questions remain unanswered, including the phylogenetic status of aurochs, whether gene flow from aurochs into early domestic populations occurred, and which genomic regions were subject to selection processes during and after domestication. Here, we address these questions using whole-genome sequencing data generated from an approximately 6,750-year-old British aurochs bone and genome sequence data from 81 additional cattle plus genome-wide single nucleotide polymorphism data from a diverse panel of 1,225 modern animals.

**Results:**

Phylogenomic analyses place the aurochs as a distinct outgroup to the domestic *B. taurus* lineage, supporting the predominant Near Eastern origin of European cattle. Conversely, traditional British and Irish breeds share more genetic variants with this aurochs specimen than other European populations, supporting localized gene flow from aurochs into the ancestors of modern British and Irish cattle, perhaps through purposeful restocking by early herders in Britain. Finally, the functions of genes showing evidence for positive selection in *B. taurus* are enriched for neurobiology, growth, metabolism and immunobiology, suggesting that these biological processes have been important in the domestication of cattle.

**Conclusions:**

This work provides important new information regarding the origins and functional evolution of modern cattle, revealing that the interface between early European domestic populations and wild aurochs was significantly more complex than previously thought.

**Electronic supplementary material:**

The online version of this article (doi:10.1186/s13059-015-0790-2) contains supplementary material, which is available to authorized users.

## Background

The development of agriculture, including plant cultivation and animal husbandry, effected a shift in subsistence from nomadic foraging and hunting to sedentary food production, and ultimately led to the appearance of the first urban civilizations [1]. Central to this process was the domestication of the now-extinct wild aurochs (*Bos primigenius*), which ranged throughout much of Eurasia and Northern Africa during the late Pleistocene and early Holocene, giving rise to the two major domestic extant cattle taxa—*B. taurus* and *B. indicus* [2, 3]. This has been confirmed by genetic analyses of matrilineal mitochondrial DNA (mtDNA) and nuclear DNA sequences, which reveal a deep pre-domestic bifurcation between modern taurine and zebu animals. These results strongly support the domestication of *B. taurus* and *B. indicus* in the Near East and Southwest Asia, respectively, from geographically disparate and genetically divergent wild cattle populations [4–7].

While genetic studies support a Near Eastern origin for European *B. taurus* cattle, there is considerable debate regarding the extent of genetic exchange between early domestic cattle and indigenous aurochs during the development of animal herding in Europe. To date, studies of ancient mtDNA sequences retrieved from preserved *B. primigenius* specimens demonstrate that the majority of Central and Northern European aurochs possess P mtDNA haplogroup sequences, which are closely related to, but phylogenetically distinct from, the *B. taurus* T macro-haplogroup. The predominance of the T macro-haplogroup in extant North and Central European *B. taurus* cattle, coupled with the almost complete absence of haplogroup P sequences, has been interpreted to mean that genetic contributions from wild females did not significantly influence early domestic cattle populations in these regions [8–11]. Conversely, the identification of sequences of putative aurochs haplogroups Q and R in modern Italian cattle does support the limited local adoption of wild aurochs matrilines in Southern Europe [12–14]. In contrast to mtDNA studies, analyses of paternally inherited Y chromosome haplotypes remain equivocal as to whether local wild male aurochs contributed to European *B. taurus* populations [15–17].

Although mtDNA and Y-chromosome studies have greatly improved our understanding of the pre- and post-domestic history of cattle, uniparental genetic loci have limited utility for reconstructing complete evolutionary histories [18]. In contrast, analyses of whole nuclear genomes or high-density single nucleotide polymorphism (SNP) data, consisting of many thousands of independently evolving loci, provide the opportunity to determine accurately the ancestry of domestic cattle and address long-standing questions regarding natural and artificial selection of key traits, as well as gene flow, introgression and admixture in early domestic and wild populations [5, 19–21].

Recently, high-throughput DNA sequencing technologies have revolutionized population genomic studies, enabling high-resolution analyses of modern and extinct genomes from human, domestic livestock and wild animal populations. Deep sequencing of mammalian livestock and their wild ancestors has revealed key genetic changes underlying the animal domestication process; in particular, how natural and artificial selection, combined with genetic drift, have shaped post-domestication patterns of genetic variation, generating the broad spectrum of phenotypic variation observed in modern domestic animal populations [19, 22–26].

**Fig. 1:**
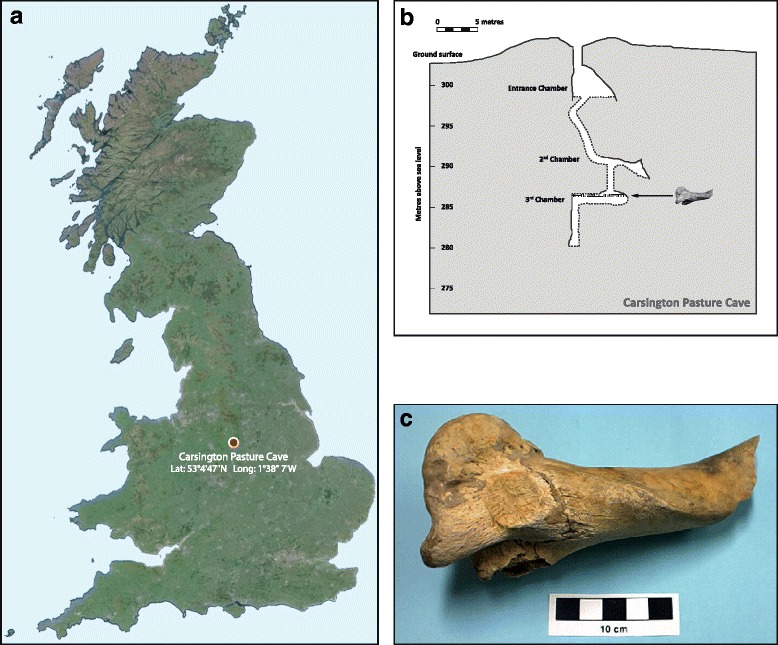
The sampling location of aurochs specimen CPC98. **a** The location of Carsington Pasture Cave, Derbyshire, England. **b** Schematic showing a cross section of the Carsington Pasture Cave system; the CPC98 aurochs humerus was retrieved from the third chamber. **c** The CPC98 aurochs humerus bone used for DNA extraction and analysis in this study

Previously, we reported the first complete *B. primigenius* mtDNA sequence using DNA extracts purified from an exceptionally well-preserved and archaeologically verified British aurochs humerus bone specimen (laboratory code CPC98; haplogroup P; [GenBank:NC_013996]) [10]. This specimen was retrieved in 1998 from Carsington Pasture Cave in Derbyshire, England, and radiocarbon dated to 6,738 ± 68 calibrated (cal.) years before present (yBP) (Fig. 1). This pre-dates the beginning of the Neolithic period in Britain (5,900–5,580 cal. yBP) [27] and also the appearance in Britain of domestic cattle characterized by uniquely mapped mtDNA macro-haplogroup T sequences, providing a secure basis for the classification of CPC98 as *B. primigenius* [28]. We now report whole-genome sequencing data from this aurochs specimen and analyse these data with 81 newly-sequenced modern *B. taurus* and *B. indicus* genomes, and with high-density SNP array data from a wide range of modern cattle populations. We discuss our findings in light of current understanding of the evolutionary history and domestic origins of cattle.

## Results and discussion

### Aurochs genome sequence coverage

Sequencing data from the *B. primigenius* nuclear genome was aligned to the *B. taurus* UMD3.1 reference genome [29]. For this purpose, six DNA libraries (C1–C6) were independently prepared from six separate aurochs bone powder extractions. These libraries were used for single-read (SR; libraries C1–C3) and paired-end (PE; libraries C4–C6) sequencing with the Illumina® Genome Analyzer IIx and HiSeq 2000 platforms (Additional file 1: Figure S1 and Table S1). Collation of data from all libraries yielded a total of 3.37 billion sequence reads, ranging in raw read length from 36 to 70 bp (Additional file 1: Table S2). Subsequent filtering to remove reads that were poorly sequenced, of low complexity, or that consisted largely or entirely of Illumina® sequencing adaptors yielded 2.86 billion filtered reads for alignment to the reference genome. Of these, 805 million reads (28.1 %) mapped to the UMD3.1 reference genome. Removal of duplicate reads (i.e. reads from the same sequencing library that mapped to the same nucleotide position on the same chromosome strand) yielded 619.7 million reads and subsequent removal of reads that mapped to multiple locations produced a total of 470 million uniquely mapped reads, of which 417 million reads mapped at Phred-scaled map quality scores ≥30 (Additional file 1: Table S3). The 470 million uniquely mapped reads comprised 16.62 Gb of aurochs nuclear and mtDNA sequences and covered 2.37 Gb of the *B. taurus* nuclear genome (89.43 % of the 2.65 Gb UMD3.1 bovine genome assembly) with a mean sequencing depth of 6.23× across all nucleotide positions (Additional file 1: Table S4). Additionally, 63,174 of these 470 million reads (comprising 2,582,767 bp) aligned to the 16,338 bp *B. primigenius* P haplogroup mtDNA sequence previously reported by us [10] [GenBank:DQ124389]. This yielded a mean sequence depth of 158.06× across all *B. primigenius* mtDNA nucleotide positions.

A scatter plot generated for the number of high-quality aligned reads to individual chromosomes revealed a uniform mean read density for all autosomes. However, the density of reads mapping to the X chromosome was approximately 50 % that of autosomal read density, demonstrating that the CPC98 bone specimen is from a male animal (Additional file 1: Figure S2).

### Aurochs genome sequence authenticity

Previous studies have shown that the analysis of ancient DNA (aDNA) is highly sensitive to two major sources of error that can result in the generation of inauthentic DNA sequence data: (1) contamination with DNA derived from exogenous, modern samples; and (2) post-mortem nucleotide modification, primarily the deamination of cytosine to uracil residues that causes a high rate of artefactual C → T transitions in newly synthesized DNA during polymerase chain reaction (PCR) amplification and sequencing [30, 31]. Therefore, we analysed possible contributions from both error sources in the aurochs genome sequence.

First, the complete CPC98 mtDNA sequence was used to estimate the amount of modern bovine DNA contamination (Additional file 1: Supplementary Methods, Section 7). For this, we catalogued mtDNA SNPs distinguishing the three currently available complete haplogroup P sequences [GenBank: NC_013996, DQ124389 and JQ437479] from a panel of 233 complete modern macro-haplogroup T and I sequences and haplogroup Q and R sequences retrieved from GenBank. This analysis identified 15 haplogroup P-discriminating SNPs. Nucleotide calls for the 15 SNP positions from the individual CPC98 reads were classified as being of aurochs or modern origin according to the allele they possessed. A total of 1,959 individual CPC98 reads spanned the 15 haplogroup P-discriminating SNPs, of which 10 reads showed an allele characteristic of T, Q, R and/or I mtDNA sequences, giving an upper estimate of modern bovine mtDNA contamination in the CPC98 specimen of 0.51 % (Additional file 1: Table S5). While this estimate is comparable to the levels of modern mtDNA contamination observed for whole Neanderthal, Denisovan and Pleistocene human genomes [32–35], it also falls within the reported sequence error rate of the Illumina® GA IIx and HiSeq 2000 platforms [36]. To further refine the estimate of modern contamination through exclusion of transitions that may be affected by diagenetic nucleotide misincorporations, we examined the number of CPC98 reads spanning the single transversion-type mutation (position 14,129). A total of 97 CPC98 reads covered this position, all displaying the P haplogroup variant, yielding a modern contamination estimate based solely on transversions of 0.00 %.

Potential nuclear DNA contamination was estimated by taking advantage of the hemizygous X chromosome in the male CPC98 specimen and through identification of diagnostic SNPs in the non-recombining portion of the X chromosome (Additional file 1: Supplementary Methods, Section 7). Using strict filtering criteria, this procedure gave upper estimates of European taurine and zebu nuclear DNA contamination of 3.4 % and 0.2 %, respectively. It is important to note that there is potential bias in these estimates because the reference genome in all alignments was generated from a European taurine Hereford animal [37]. Therefore, false reads carrying the European taurine allele will have higher alignment scores than false reads carrying the zebu allele, which would tend to elevate the European taurine contamination estimate relative to the zebu estimate. In addition, this procedure assumes that there are no assembly errors or copy-number variations that would alter the expectation of haploid genotypes at these X-chromosomal SNP positions in CPC98.

Variation in the distribution of DNA fragment lengths obtained from ancient specimens has also been used as an indirect measure to estimate the extent of modern DNA contamination [38]. Post-mortem fragmentation is a feature of aDNA, with authentic endogenous sequences generally having a fragment size under 200 bp [38, 39]. The median aurochs fragment size was estimated to be 50 bp using the uniquely mapped PE sequence reads (17.8 million reads comprising 994.4 Mb), with 99.99 % of mappable inserts ranging between 16 and 150 bp and 99.19 % of these ranging between 20 and 150 bp (Additional file 1: Figure S3). These results are consistent with endogenous DNA fragment lengths reported for Palaeolithic hominin specimens, which typically display mean sizes of 30–100 bp [32–35].

We previously estimated the extent of DNA sequencing error for the CPC98 specimen by counting non-consensus nucleotide calls from individual mtDNA reads that differed from the CPC98 mtDNA consensus sequence [10]. We repeated this analysis using the larger number of sequence reads mapping to bovine mtDNA haplogroup P genome sequence obtained for the present study. Notably, increasing the number of mtDNA sequence reads did not reveal any excess of the characteristic C → T transitions due to post-mortem cytosine deamination within the individual CPC98 sequence reads. The absence of significant post-mortem cytosine deamination may be attributable to the Phusion High-Fidelity DNA polymerase used during library preparation. This polymerase has been shown to inefficiently amplify DNA fragments containing uracil residues that have been generated via the deamination of cytosine [40, 41]; however, this enzyme can amplify fragments containing thymine residues that have been generated by post-mortem deamination of endogenous 5′-methyl-cytosines (5meC). Therefore, we acknowledge that while the DNA sequence errors observed in the current study are most likely artefacts of the DNA sequencing methods used, individual CPC98 reads displaying C → T transitions may have been generated, in part, by 5meC post-mortem deamination processes [40, 42, 43].

The mapDamage 2.0 analysis results for a subset of sequence reads highlight increases in C → T and G → A transitions typical of aDNA damage patterns at the 5′ and 3′ ends of reads, respectively (Additional file 1: Figure S4). However, these increases are lower than those observed in other bones of similar age; for example, those recently reported by Gamba and colleagues [44]. Possible explanations for this reduction in observable DNA damage may include the use of Phusion polymerase as documented above, as well as the Illumina® AT-overhang ligation step used in the library preparation protocol, which has previously been shown to prohibit the ligation of damaged cytosines [45].

Taken together these results support the authenticity of the *B. primigenius* sequence reads obtained from the CPC98 libraries.

### Aurochs DNA sequence variant analysis

A total of 5,233,471 DNA sequence variants differentiating the CPC98 and reference genome were identified. Of these, 2,135,925 passed a composite quality filter including a minimum read depth threshold of 5× and a recalibrated logarithm of the odds (LOD) score greater than 2 (Table 1). In brief, these ~2.1 million variants comprised 2,009,261 biallelic SNPs (73.3 % of which were homozygous) and 104,655 indels (86.3 % of which were homozygous). The transition to transversion (ti/tv) ratio was estimated at 2.19:1.00, which is similar to the ti/tv ratio obtained from a female Holstein genome sequence (2.18:1.00) [46]. The Ensembl Variant Effect Predictor (VEP) package [47] was used to determine the effect of the aurochs DNA sequence variations (i.e*,* SNPs and indels) on genes, transcripts, protein sequence and regulatory regions (Additional file 2). We found that 96.9 % of the CPC98 SNPs and 94.2 % of the CPC98 indels were previously described and present in dbSNP build 140. Homozygous variants were noticeably more likely to be previously described, and transition SNPs slightly more likely than transversions (Table 1). We also cross-referenced the CPC98 variants to a set of SNPs and indels detected by shallow coverage genome re-sequencing data (128.4×) from 81 modern taurine and zebu samples representing 11 breeds originating from Europe, Africa and India (Additional file 1: Supplementary Methods, Section 8.2). In these re-sequenced modern animals, 84.9 % of CPC98 SNPs and 81.3 % of CPC98 indels were present. The high proportion of CPC98 SNPs and indels that are previously described supports the authenticity of these variants.

**Table 1:** SNP and indel variants detected between the *B. taurus* UMD3.1 reference genome and aurochs specimen CPC98

Variant type	CPC98 genotype^a^	Transition or transversion	Count	Present in dbSNP 140^b^	Confirmed in modern re-sequenced animals^c^
SNP	Heterozygous	Transition	368,538	93.1 %	72.7 %
		Transversion	166,652	91.5 %	70.0 %
	Homozygous	Transition	1,010,894	98.6 %	90.1 %
		Transversion	463,177	98.3 %	88.8 %
Weighted mean				96.9 %	84.9 %
Indel	Heterozygous	-	14,309	80.6 %	59.2 %
	Homozygous	-	90,346	96.4 %	84.8 %
Weighted mean				94.2 %	81.3 %

^a^The CPC98 genotype is given as homozygous for positions where the CPC98 sequence is homozygous for a different allele to that in the reference genome, and heterozygous where CPC98 carries only one allele that differs from the reference

^b^The percentage of variants that are recorded in dbSNP version 140

^c^The percentage of variants that were observed in the panel of 81 sequenced modern animals

### North European *Bos primigenius*: an evolutionary outgroup of modern taurine cattle

A large reference SNP data set for phylogenetic and population genetic analyses was assembled from previously published studies. This consisted of Illumina® BovineSNP50 genotype data from 1,228 individual cattle comprising the CPC98 specimen; 1,225 samples from 73 modern cattle populations (representing European and African *B. taurus*, Asian *B. indicus*, and various crossbred *B. taurus* × *B. indicus* and African *B. taurus* × European *B. taurus* populations); and two yak (*B. grunniens*) samples (Additional file 1: Table S6). This SNP data set was filtered to retain 15,498 autosomal SNPs covered at a read depth ≥10 in the CPC98 genome sequence data and a genotyping rate ≥90 % across all 1,228 animals in the BovineSNP50 data set. Further information on this reference SNP data set is provided in Additional file 1: Supplementary Methods, Section 8.3.

**Fig. 2:**
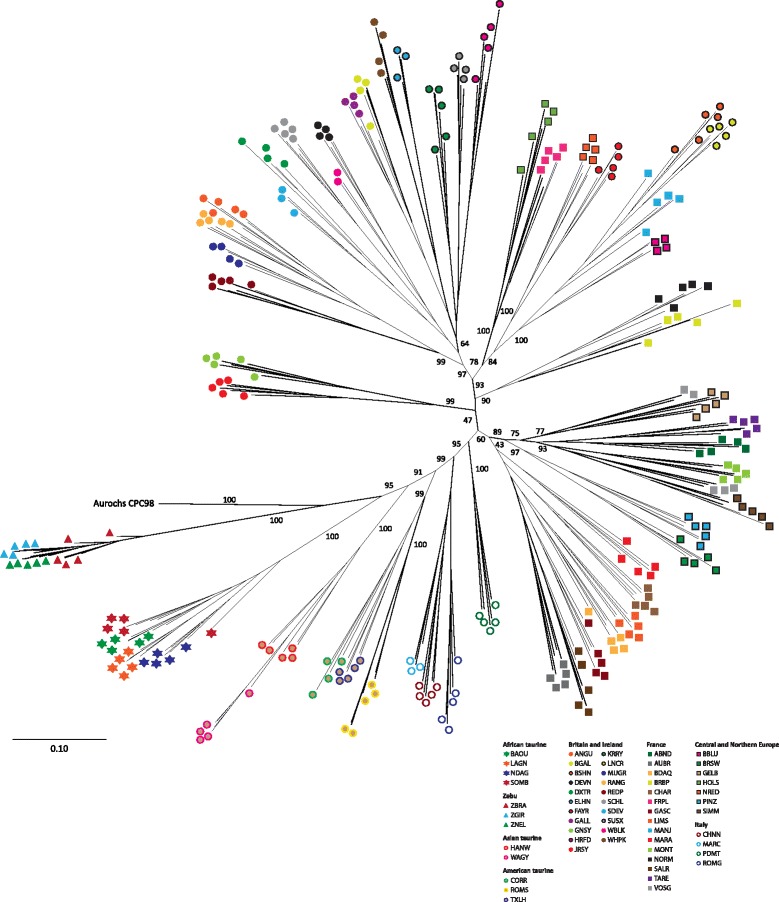
Maximum likelihood phylogenetic tree constructed with a reduced panel of 278 animals using 10,923 high-quality filtered single nucleotide polymorphisms. Bootstrap support values for the phylogeny were generated using 100 pseudoreplicates of the single nucleotide polymorphism genotypes and *p*-distance values were used for the evolutionary distances between individual animals

**Fig. 3:**
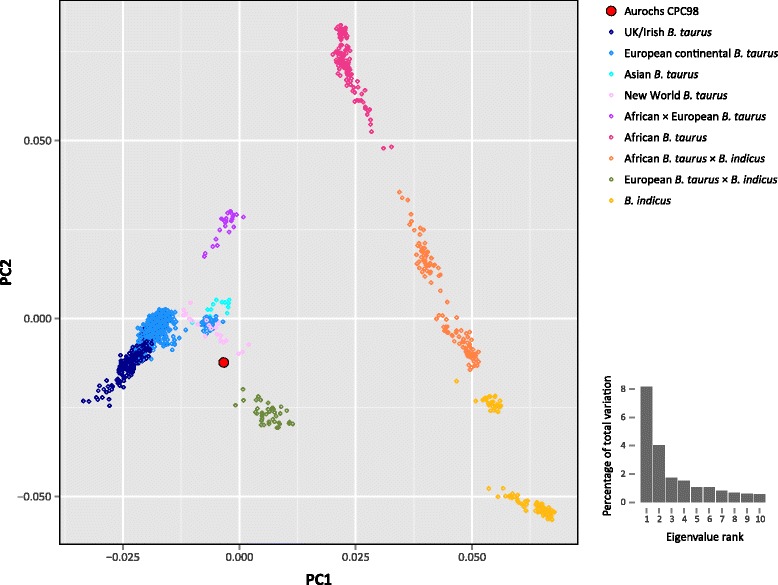
Principal component analysis plot constructed for principal component (*PC*) 1 and PC2 are shown for 1,226 animals using 12,874 high-quality filtered single nucleotide polymorphisms. The position of the aurochs CPC98 specimen is highlighted. The smaller histogram plot shows the relative variance contributions for the first 10 PCs

The SNPhylo software package [48] was used with the cattle SNP data set to generate a maximum likelihood (ML) phylogenetic tree (Fig. 2). For this analysis, the BovineSNP50 data set was filtered using a linkage disequilibrium threshold (*r*^2^ ≤ 0.5) to generate a subset of 10,923 high-quality SNPs. For visual clarity, the tree was constructed with a reduced panel of 278 representative cattle (a maximum of five randomly sampled animals per breed from 59 modern breeds excluding the *B. taurus* × *B. indicus* and African *B. taurus* × European *B. taurus* crossbred populations) (Additional file 1: Table S6). Bootstrap support values for the phylogeny were generated using 100 pseudoreplicates of the SNP genotypes. The phylogeny presented in Fig. 2 was also validated with an additional ML tree (Additional file 1: Figure S5) with Bayesian-based support for each branch generated using the PhyML package [49]. The tree shows complete separation of taurine and zebu populations, illustrating the evolutionary divergence between the two taxa and supporting the separate domestic origins of *B. taurus* and *B. indicus* [4–7]. It is important to note, however, that SNP ascertainment bias inherent in the Illumina® BovineSNP50 assay design may have a detectable effect on the estimated divergence between the European *B. taurus* and other cattle groups [50]. The British CPC98 aurochs specimen is an outgroup—with 100 % bootstrap support—to the entire modern *B. taurus* clade in this phylogeny. This is corroborated by the principal component analysis plot shown in Fig. 3 in which CPC98 is peripheral to the European *B. taurus* sample group along principal component 1. These results provide strong support for the hypothesis that the North European aurochs is an evolutionary outgroup to all domesticated taurine cattle [8, 28].

**Fig. 4:**
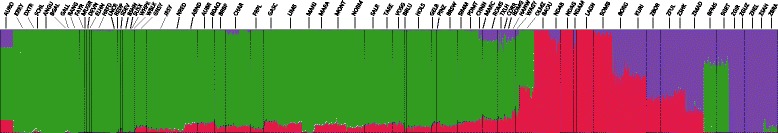
Unsupervised hierarchical clustering of 1,226 individuals (comprising 73 populations, where the N’Dama from Burkina Faso were considered as two distinct populations) using genotype data for 7,749 high-quality filtered single nucleotide polymorphisms. Results for an inferred number of clusters *K* = 3 are shown. *Green* European taurine, *red* African taurine, *purple* zebu

To investigate the genetic structure and admixture history of the populations and aurochs analysed, STRUCTURE [51] was run using BovineSNP50 data from all samples (Additional file 1: Table S6). The results of this analysis for *K* = 3 are shown in Fig. 4 and STRUCTURE plots for additional *K* values (4 and 5) are shown in Additional file 1: Figure S6. Using all geographical groups, the three-population model (*K* = 3) differentiates the European taurine, African taurine and zebu components. The CPC98 aurochs specimen displays contributions from each of these three biogeographical groups. The zebu component detected in three of the four Italian populations (Chianina, Marchigiana and Romagnola) and the two East Asian taurine populations (Hanwoo and Wagyu) has been hypothesized to represent historical admixture from *B. indicus* [20, 52]. However, the tripartite genetic structure observed in the CPC98 aurochs specimen supports an alternative hypothesis, that alleles now exclusive to African and Asian cattle may once have been present more widely prior to domestication, but were subsequently lost from the European domesticated *B. taurus* lineage. Whether some native Italian and East Asian taurine breeds have retained pre-domestic alleles lost elsewhere in Europe or acquired additional alleles through hybridization with aurochs remains an unresolved question.

### Hybridization between aurochs and domestic cattle populations in Europe

Studies of uniparental genetic loci (mtDNA and Y-chromosome haplotypes) and autosomal marker variation have been equivocal on the question of whether early European domestic cattle populations interbred with local wild aurochs [12–14, 28, 53–55]. The presence of putative *B. primigenius* P, R, Q and, most recently, C mtDNA haplogroups in modern and early domestic cattle populations from Europe and Asia has provided some support for the local domestication of wild aurochs females in Europe. However, the vast majority of extant taurine cattle mtDNA haplotypes (T haplogroup) derive from cattle domesticated in the Near East, so it is likely that local adoptions of wild matrilines into domestic pools were rare. Based on discontinuous distributions of two distinct Y-chromosome haplogroups (Y1 and Y2) in European taurine bulls, and genotyping of European male aurochs samples, it has previously been hypothesized that Northern European cattle retain a Y haplogroup (Y1) indicative of ancient hybridization between wild *B. primigenius* males and domestic *B. taurus* females [16]. However, subsequent work has seriously challenged this interpretation and it is now generally accepted that the currently available bovine Y-chromosome genetic markers (SNPs and microsatellites) are incapable of resolving the question of whether wild European aurochs bulls mated with domestic female cows [11, 15, 17, 56–58].

**Fig. 5:**
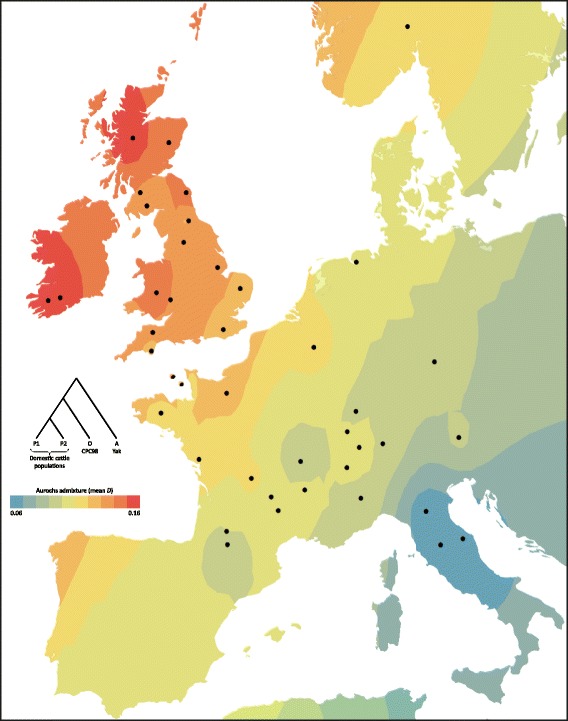
Geographic contour map of aurochs genomic admixture with individual European breed *D* statistics (ABBA/BABA test results) plotted according to population origin and visualized using the ArcMap component of the ArcGIS software suite. The ABBA/BABA test tree topology is also shown and the contour point value for each European breed (P1) was generated from the mean *D* statistic where P2 is set to each of seven West African taurine populations in turn

To assess the evidence for post-domestication hybridization between British aurochs and the ancestors of modern European cattle, we used the ABBA/BABA test of genomic admixture [59]. The results of this analysis for individual European breeds are shown in Additional file 1: Table S7, while Additional file 1: Table S8 summarizes the results of the block jackknife resampling procedure used to test significance of the *D* statistic comparisons among the major geographical breed groupings. Figure 5 shows a contour map of the individual European breed *D* statistics plotted according to population origin. These results provide compelling evidence supporting the hypothesis of a significant genetic contribution from British aurochs to modern domestic cattle populations from Britain and Ireland. In particular, inspection of Additional file 1: Table S7 and Fig. 5 demonstrates that this ancient genetic legacy is most apparent in traditional or landrace cattle breeds of Scottish, Irish, Welsh and English origin (i.e. Highland, Dexter, Kerry, Welsh Black and White Park).

**Fig. 6:**
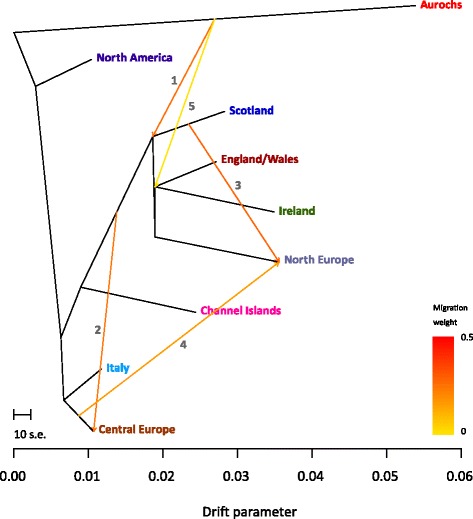
TreeMix maximum likelihood phylogeny with migration edges constructed using genotype data (15,498 single nucleotide polymorphisms) from eight European and American regional cattle population groups and the single CPC98 aurochs specimen. This phylogeny was generated with five migration edges with the first migration edge (labelled *1*) hypothesized to be introgression from wild British aurochs into domestic cattle ancestral to the British, Irish and Northern European clade. The fifth migration edge (*5*) is hypothesized to be another introgression of wild British aurochs into domestic cattle ancestral to the England/Wales and Ireland subclade. The scale bar shows 10 times the mean standard error of the estimated entries in the sample covariance matrix

Interpopulation migration between British aurochs and European taurine population groups was further investigated using phylogenies and ancestry graphs generated with the TreeMix program [60]. Additional file 1: Figures S7, S8 and Fig. 6 shows the results of these analyses. It is noteworthy that two migration edges from British aurochs to the ancestors of British and Irish cattle are evident in Fig. 6 (numbers 1 and 5). These results also provide support for the hypothesis of significant gene flow from British aurochs into the progenitors of modern cattle from Britain and Ireland. It is important to note, however, that the three-population test for admixture [61] did not detect evidence of significant admixture for any of the 252 possible three-population combinations generated by the threepop program for the aurochs and the eight cattle populations shown in Fig. 6.

### Functional analyses of the aurochs and modern cattle genomes

We performed functional genomic analyses using whole-genome sequencing data from the CPC98 aurochs genome and the genomes of 81 newly sequenced modern *B. taurus* and *B. indicus* samples (Additional file 1: Supplementary Methods, Section 8.2) to investigate how domestication may have shaped the genomes of modern taurine cattle.

We screened for functionally significant SNP and indel variants arising post-domestication in European *B. taurus* and increasing to fixation, possibly through selection. SNPs and indels were filtered to retain those that were near fixation (≥95 %) for different alleles in the European *B. taurus* and *B. indicus* cattle groups, and for which the CPC98 aurochs specimen was homozygous for the *B. indicus* major allele. The Ensembl VEP tool was used to filter these further to 263 gene-associated variants by excluding intronic and intergenic variants, and retaining only variants in exons, 5′ and 3′ untranslated regions (UTRs), and flanking sequences 5 kb upstream or downstream of gene boundaries (Additional file 1: Table S9). These 263 variants were hypothesized to have emerged in the European *B. taurus* lineage post-domestication and eight missense mutations identified as part of this analysis are detailed in Additional file 1: Table S10. It is noteworthy that two of these missense mutations were located in olfactory receptor genes (ENSBTAG00000024891, ENSBTAG00000019925). In recent years, comparative whole-genome analyses have highlighted olfaction genes as targets of selection during and after domestication for a range of mammalian species, including cats, dogs, pigs and horses [23, 24, 62–66]. One of the eight missense mutations was also observed in the *DGAT1* gene, which harbours a major quantitative trait nucleotide for milk traits and has been subject to intense selection in many dairy cattle populations [67–69].

One hundred and ninety-three genes were associated with the 263 post-domestication European *B. taurus* variants and these were analysed using Ingenuity® Systems Pathway Analysis (IPA). The subset of 166 genes mappable to molecules within the IPA Knowledge Base were enriched for neurobiological, immune signalling, and growth and metabolism functions (Additional file 1: Table S11 and Table S12), suggesting that these biological processes played an important role in the domestication and subsequent evolution of European *B. taurus* cattle.

We also used the Hudson-Kreitman-Aguadé (HKA) test to investigate genomic regions under selection in the *B. taurus* lineage. The HKA test is based on the ratio of diversity within an ingroup of DNA sequences to the distance to an outgroup [70]. In the absence of selective sweeps, this ratio is expected be constant throughout the genome; however, selective sweeps, which cause a reduction in effective population size, are expected to attenuate ingroup DNA sequence diversity. All *B. taurus* genome sequences and the CPC98 aurochs genome were included for HKA analysis, with *B. taurus* considered as the ingroup and the CPC98 specimen as the outgroup. The genome regions displaying positive selection are shown in Additional file 1: Figure S9. The HKA results shown in Fig. S9 were not significant when adjusted for multiple testing (Benjamini-Hochberg false discovery rate method [71]); therefore, these results should be considered suggestive. Notwithstanding this, within regions showing positive selection in the *B. taurus* lineage at *P* ≤ 0.05 (Additional file 1: Table S13), we found a total of 106 annotated unique candidate loci (including protein-coding and miRNA-coding genes). Of these, 96 genes mapped to molecules within the IPA Knowledge Base and were used to identify biological functional categories enriched for genes potentially under positive selection.

Among the top-ranking functional categories for the HKA analyses (Additional file 1: Table S14) were genes involved in neurobiology, and muscle development and function, which supports the hypotheses that the domestication process was accompanied by selection for behavioural and meat traits. Functional pathway analysis also revealed enrichment of genes involved in haematological system development and immunity (Additional file 1: Table S15). This observation may reflect the adaptation of early domesticates to increased interaction with humans and concomitant exposure to novel pathogens due to increased confinement under managed systems of animal breeding and husbandry [3, 72]. Indeed, recent studies in domestic mammals have reported that immune-related genes are subject to positive selection against rapidly evolving pathogens [73, 74].

Finally, to identify polymorphisms associated with differential miRNA regulation, we screened 659 orthologous miRNA genes for polymorphisms differentiating the aurochs genome from the 81 modern *B. taurus* genomes sequenced for this study (Additional file 1: Supplementary Methods, Section 8.2). We identified five miRNA genes containing mature miRNA polymorphisms, including one polymorphism in the seed region of miR-2893 (Additional file 1: Figure S10). Such miRNA gene polymorphisms can lead to regulatory shifts in the set of mRNAs targeted by a miRNA (Additional file 1: Table S16). IPA analysis of the different sets of gene targets revealed enrichment of canonical pathways involved in axonal guidance, mTOR signalling and androgen signalling for the *B. taurus* bta-miR-2893 variant, but not for the *B. primigenius* bpr-miR-2893 variant. In contrast, melanocyte development and pigmentation signalling pathways were enriched for gene targets of the aurochs bpr-miR-2893 variant but not for bta-miR-2893 (Additional file 1: Figure S11). Among the genes targeted by the bpr-miR-2893 variant but not the bta-miR-2893 variant were *PHYHIP* and *FADS2*, which are involved in neurodevelopment and fatty acid metabolism, respectively [75, 76]. Conversely, genes targeted specifically by the bta-miR-2893 variant but not the bpr-miR-2893 variant included *FCRL1,* which has an immunological role [77]. These results indicate that selection of miRNA regulatory variants (such as bta-miR-2893) with roles in neurobiology, immune function, and growth and metabolism have contributed to the recent evolution of modern cattle.

## Conclusions

To date, comparative analyses of aurochs and modern cattle DNA sequences have relied on non-recombining uniparental loci that are not representative of ancestry across the whole genome. Therefore, it is important that information obtained from mtDNA and Y-chromosomal DNA is complemented with data from autosomal polymorphisms. In this regard, comparative analysis of an aurochs genome with genome information from modern animals highlights the complex domestic history of cattle, most notably a Northern Eurasian aurochs component to the genetic ancestry of modern British and Irish cattle, which may have arisen through purposeful restocking with wild aurochs by early herders in Britain. Our results also revealed a number of genes associated with neurobiology, growth and metabolism, and immunobiology that exhibit evidence for positive selection within the time frame since cattle domestication ~10,000 years ago. Sequencing data from the aurochs genome provides an important reference for testing hypotheses regarding the genetic consequences of recent artificial and natural selection in modern cattle. In addition, future analyses of whole-genome sequences from modern animals and additional aurochs samples will refine the catalogue of genomic loci that contribute to key functional traits in domestic cattle.

## Methods

Detailed descriptions of laboratory and analytical methods are provided in Additional file 1: Supplementary Methods.

### Archaeological specimen details

The aurochs specimen analysed in this study (laboratory code CPC98) consists of the proximal half of a humerus retrieved from chamber 3 of Carsington Pasture Cave, Derbyshire, England [78]. Figure 1 shows the geographical location of Carsington Pasture Cave, a vertical section of the cave system, showing chamber 3, and a photograph of the CPC98 humerus bone.

### Aurochs CPC98 humerus bone DNA extraction

All DNA extraction, purification and Illumina® sequencing library preparation steps were performed in a dedicated ancient DNA laboratory located at the Department of Genetics, Trinity College, Dublin, Ireland. The C1, C2 and C3 DNA extracts were prepared from three independently generated bone powder samples obtained from different locations on the aurochs CPC98 humerus. The methods used for bone powder processing and DNA extraction and purification for the C1 − C3 samples have been fully described by us elsewhere [10]. The C1 − C3 DNA extracts were used for Illumina® single-end library preparation [10].

For the present study, three additional DNA extracts (C4, C5 and C6) were generated from three further independent bone powder samples using our previously described methods. Three separate ~30 μl aliquots were prepared from each of the three DNA extracts (labelled C4_1–3_, C5_1–3_ and C6_1–3_); all nine DNA aliquots were used to generate paired-end CPC98 Illumina® sequencing libraries.

Additional file 1: Figure S1 shows a schema detailing the laboratory procedures used for the preparation of the C1 − C6 DNA extracts, the Illumina® sequencing libraries generated, the sequencing platforms used and the sequencing centre locations.

### High-throughput sequencing of aurochs libraries

Pooled Illumina® sequencing libraries C1 − C3 generated from three aliquots of each extract were previously sequenced and analysed by us using the Illumina® Genome Analyzer IIx (GA IIx) platform (Illumina, Inc., San Diego, CA, USA) [10]. For the present study, additional sequencing data were generated from these libraries using the Illumina® HiSeq 2000 platform at the Beijing Genome Institute (BGI), Shenzhen, China, using 49 bp reads.

The two single-end Illumina® GA sequencing libraries, C1 and C2, were used for targeted enrichment of bovine genes using the Agilent SureSelect oligonucleotide hybridization solution-based capture system (Agilent Technologies, Santa Clara, CA, USA). The complete list of targeted genes is provided in Additional file 1: Table S17.

The nine newly purified CPC98 DNA extract aliquots (C4_1–3_, C5_1–3_ and C6_1–3_) were used for Illumina® paired-end DNA sequencing. Individual libraries were combined according to their initial extract number (C4_1–3_, C5_1–3_ and C6_1–3_) to form three final pooled paired-end libraries labelled C4, C5 and C6, respectively (Additional file 1: Figure S1). Sequencing of paired-end libraries was performed at the Animal Genomics and Improvement Laboratory, Agricultural Research Service, USDA, Beltsville, MD, USA, and at the Conway Institute of Biomolecular and Biomedical Research, University College Dublin, Ireland (unidirectional sequencing only).

### Preliminary bioinformatics, genome mapping and SNP/indel calling

The UMD3.1 bovine genome build was used with the haplogroup P complete mtDNA genome sequence [GenBank:DQ124389] replacing the UMD3.1 reference mtDNA sequence. Sequence read quality filtering and trimming was performed and trimmed reads were aligned to the UMD3.1 bovine genome sequence using the BWA software package [79]. Following this, two rounds of duplicate removal, SNP-indel sensitive read realignment and read mate position fixing were performed using the GATK software package [80], polymorphism data from dbSNP Build 133 [81] and picard tools [82]. The final individual library files were merged to give a single alignment file incorporating all uniquely aligned non-duplicate reads. Following this, the mapDamage 2.0 software package was used to examine aDNA preservation and damage parameters [83]. In addition, estimates of exogenous modern bovine mtDNA and nuclear DNA contamination of CPC98 sequence reads were generated by exploiting diagnostic SNP positions in the mtDNA genome and on the X chromosome.

### Identification of SNP and indel variation

SNP and indel variants differentiating the CPC98 sequence from the *B. taurus* reference genome were identified using the GATK software package. An initial round of variant calling was used to identify very high confidence SNPs and indels from within the CPC98 data, and thence to generate a Gaussian model of variant confidence incorporating a range of sequence read and variant parameters. This model was then applied to the full list of variants, and only high confidence variants were retained. Additional filtering ensured that all variants were covered by at least five sequence reads as well as satisfying several further confidence criteria. Variant calling identified 2,009,261 SNPs and 104,655 indels distinguishing the CPC98 genome from the *B. taurus* reference genome (Table 1). Of the 2,009,261 high confidence SNPs, 99.9 % had a read depth between 5 and 31. Of the 104,655 high confidence indels, 99.9 % had a read depth between 5 and 24.

### Comparative sequence data from low-coverage cattle genome sequencing

Shallow Illumina® sequencing was performed using a panel of 81 individual cattle samples from 11 different *B. taurus* and *B. indicus* breeds to give a composite sequence coverage of 128.4×. Stringent SNP filtering resulted in 18,794,430 SNPs and 1,480,183 indels.

### Population genomics, phylogenetics and functional analyses

To assemble a bovine SNP population reference data set, nucleotide calls were obtained for the CPC98 aurochs at nucleotide positions represented on the Illumina BovineSNP50 v2 BeadChip; in addition, BovineSNP50 genotype data from a further 1,524 modern cattle and yak (*B. grunniens*) samples were obtained from previously published work. After quality control and filtering, a data set comprising 15,498 autosomal SNPs genotyped in 1,225 cattle from 73 modern populations and two yak samples was generated for downstream analyses (Additional file 1: Table S6).

STRUCTURE analysis was performed with 7,749 SNP loci and a range of assumed populations *K* from 2 to 25 was explored [51]. Outputs were visualized using the DISTRUCT program [84]. The SNPhylo software package [48] was used to construct a ML phylogenetic tree with a filtered subset of 10,923 high-quality SNPs and a reduced panel of 278 cattle (≤5 animals per breed from 60 modern breeds). A bootstrapped ML tree was visualized and annotated using the FigTree program [85]. An additional ML tree with Bayesian branch support values was produced using the PhyML package [49] and PCs were generated using SMARTPCA from the EIGENSOFT 4.2 software package [86]. The TreeMix software package [60] was also used for phylogenetic analyses and to investigate interpopulation migration using ancestry graphs. The BovineSNP50 reference SNP data set (15,498 SNPs) for 50 taurine cattle breeds from Europe and the Americas grouped into regional population groups was used with the single CPC98 aurochs specimen and the Brahman zebu breed as an outgroup. In addition, the three-population test for admixture described by Reich and colleagues [61] and implemented with the threepop program in the TreeMix software package was used for the CPC98 aurochs and the eight regional population groups with blocks of 50 SNPs as described above.

The ABBA/BABA (*D* statistic) test [59] was performed using the primary set of 15,498 autosomal SNPs to identify breeds showing significant admixture with British aurochs. ABBA/BABA tests were run using a population-level test, with significance assessed using a weighted block jackknife as described by Green and colleagues [87]. The ABBA/BABA test uses four populations: an outgroup species that diverged from the other three early in their history (O, *B. grunniens*); an archaic population, the putative source of the gene flow (A, the CPC98 aurochs); and two closely related test populations (P1 and P2). The tree topology is therefore (((P1, P2), A), O) and the test is whether there has been gene flow between A and P1, or conversely between A and P2. For populations P1 and P2, all possible pairwise combinations of the 60 taurine populations were used, with the Burkina Faso N’Dama considered as two distinct populations as described by Gautier and colleagues [88]. A geographical representation of individual European breed *D* statistics was visualized as a contour plot using the means of the seven pairwise comparisons to the West African taurine populations (Baoulé, Lagune, Somba and four separate N’Dama populations [Burkina Faso × 2, Gambia, Guinea]) for each European taurine population.

We evaluated autosomal and X chromosomal high-confidence SNPs (*n* = 2,006,065) and indels (*n* = 104,655) distinguishing the CPC98 genome sequence from the *B. taurus* UMD3.1 reference genome for potential functional effects using the Ensembl VEP tool (build 78). We also analysed SNPs (*n* = 6,762,918) and indels (*n* = 83,218) identified by shallow resequencing in modern animals and callable in the CPC98 genome for functional effects, filtering to include only variants fixed or near fixed (≥95 %) for different alleles in *B. taurus* and *B. indicus,* and for which CPC98 was called as homozygous for the *B. indicus* allele. Results were annotated with gene symbols, names and coordinates obtained from Ensembl build 78.

To detect evidence of selective sweeps, we applied a modified genome-wide multilocus partial HKA test method (HKAdirect) as described by Esteve-Codina and colleagues [89]. Genes overlapping the detected regions were tabulated and subject to downstream systems analyses using IPA and the IPA Knowledge Base.

Aurochs 3′ UTR and miRNA sequences were retrieved by mapping the coordinates of bovine genes (UMD3.1) to the genome of the CPC98 aurochs. Unannotated mammalian miRNAs in the bovine genome were identified in aurochs using MapMi [90], and TargetScan 6.1 [91] was used to identify and score the different miRNA target sites. This score was then used to establish the *SSr* scoring system based on the total score of miRNA binding sites of each gene and the ratio of variant and common binding sites identified. Following this, IPA was used to identify enriched canonical pathways for genes with polymorphic miRNA binding sites, and significantly enriched pathways were ranked based on the average score of miRNA-targeted genes in these pathways.

### Data availability

The CPC98 aurochs sequence read data and bam alignment file is available from the NCBI BioProject Repository (project number: PRJNA294709). The additional sequence read data generated from 81 individual cattle samples from 11 different *B. taurus* and *B. indicus* breeds is also available from the NCBI BioProject Repository (project numbers: PRJNA295800, PRJEB2359, PRJEB2364 and PRJEB1985). The Illumina BovineSNP50 v2 BeadChip SNP genotype data was assembled from published studies as detailed in Additional file 1: Table S6.

## Additional files

Additional file 1:
**Supplementary information.** Supporting figures, supporting tables and supplementary methods. (DOCX 4141 kb) Additional file 2:
**Variant effect predictor results.** Detailed results from the variant effect predictor (VEP) analyses applied to the CPC98 aurochs and modern animal SNPs and indels. (XLSX 114 kb)
