# Supplementary material for: Genome sequencing of the extinct Eurasian wild aurochs, Bos primigenius, illuminates the phylogeography and evolution of cattle
Source: Genome Biol. 2015 Oct 26;16:234. doi: 10.1186/s13059-015-0790-2 (PMC4620651; doi:10.1186/s13059-015-0790-2)
Supplement: Additional file 1: — Supplementary information. Supporting figures, supporting tables and supplementary methods. (DOCX 4141 kb) [file 13059_2015_790_MOESM1_ESM.docx]

**Genome sequencing of the extinct Eurasian wild aurochs, *Bos primigenius*, illuminates the phylogeography and evolution of cattle**

**Supplementary Information**

Stephen D.E. Park^1^, David A. Magee^2,3^, Paul A. McGettigan^2^, Matthew D. Teasdale^4^, Ceiridwen J. Edwards^5^, Amanda J. Lohan^6^, Alison Murphy^6^, Martin Braud^7^, Mark T. Donoghue^7^, Yuan Liu^8^, Andrew T. Chamberlain^9^, Kévin Rue-Albrecht^2^, Steven Schroeder^10^, Charles Spillane^7^, Shuaishuai Tai^8^, Daniel G. Bradley^4^, Tad S. Sonstegard^10^, Brendan J. Loftus^6,11^ and David E. MacHugh^2,6,*^

**Institutional addresses of authors:**

^1^ IdentiGEN Ltd., Unit 2, Trinity Enterprise Centre, Pearse Street, Dublin 2, Ireland;

^2^ Animal Genomics Laboratory, UCD School of Agriculture and Food Science, University College Dublin, Belfield, Dublin 4, Ireland;

^3^ Department of Animal Science, University of Connecticut, Storrs, Connecticut 06029, USA;

^4^ Smurfit Institute of Genetics, Trinity College, Dublin 2, Ireland;

^5^ Research Laboratory for Archaeology and the History of Art, Dyson Perrins Building, South Parks Rd, Oxford, OX1 3QY, United Kingdom;

^6^ UCD Conway Institute of Biomolecular and Biomedical Research, University College Dublin, Dublin 4, Ireland;

^7^ Genetics and Biotechnology Laboratory, Plant and AgriBiosciences Research Centre (PABC), School of Natural Sciences, National University of Ireland Galway, University Road, Galway, Ireland;

^8^ BGI Shenzhen, Beishan Industrial Zone, Yantian District, Shenzhen, 518083, China;

^9^ Faculty of Life Sciences, University of Manchester, Oxford Road, Manchester, M13 9PT United Kingdom;

^10^ Animal Genomics and Improvement Laboratory, Agricultural Research Service, USDA, Beltsville, MD 20705‐2350, USA;

^11^ UCD School of Medicine, University College Dublin, Belfield, Dublin 4, Ireland.

^*^ Correspondence and requests for materials should be addressed to D.E.M.

Contents

[Table of Supporting Figures 3](#_Toc430893453)

[Table of Supporting Tables 4](#_Toc430893454)

[Supporting Figures 5](#_Toc430893455)

[Supporting Tables 32](#_Toc430893456)

[Supplementary Methods 82](#_Toc430893457)

[1. Aurochs CPC98 humerus bone DNA extraction 82](#_Toc430893458)

[2. High-throughput sequencing of aurochs single-read libraries 83](#_Toc430893459)

[3. Paired-end aurochs library preparation and sequencing 84](#_Toc430893460)

[3.1. Blunt end-repair of aurochs DNA extracts 84](#_Toc430893461)

[3.2. Generation of 3'-dATP overhangs on the end-repaired aurochs DNA extracts 84](#_Toc430893462)

[3.3. Illumina® adaptor ligation 84](#_Toc430893463)

[3.4. PCR amplification of purified end-repaired, adaptor-ligated DNA templates 85](#_Toc430893464)

[3.5. High-throughput sequencing of aurochs paired-end libraries C4-C6 85](#_Toc430893465)

[4. Targeted enrichment of aurochs nuclear genes 86](#_Toc430893466)

[5. Bovine genome template sequence 87](#_Toc430893467)

[6. Alignment of Illumina sequence reads to genome sequences 88](#_Toc430893468)

[7. Assessment of aurochs genome sequence authenticity 89](#_Toc430893469)

[8. Population genomics, phylogenetics and functional analyses 90](#_Toc430893470)

[8.1. Identification of SNP and indel variation 91](#_Toc430893471)

[8.2. Comparative sequence and SNP data from shallow cattle genome sequencing 92](#_Toc430893472)

[8.3. Assembly of BovineSNP50 SNP reference data set 92](#_Toc430893473)

[8.4. STRUCTURE analysis 93](#_Toc430893474)

[8.5. Phylogenetic tree construction 94](#_Toc430893475)

[8.6. Principal components analysis 96](#_Toc430893476)

[8.7. ABBA/BABA test for genomic admixture 96](#_Toc430893477)

[8.8. Variant effect predictor analyses applied to CPC98 and modern animal SNPs and indels 97](#_Toc430893478)

[8.9. HKA test for detection of selective sweeps 98](#_Toc430893479)

[8.10. Identification and ranking of miRNA targets in cattle and aurochs 99](#_Toc430893480)

[References 101](#_Toc430893481)

Table of Supporting Figures

[Figure S1. Schematic of the DNA extracts and Illumina® single read and paired-end sequencing libraries used for the generation of the CPC98 genome sequence. 5](#_Toc430893436)

[Figure S2. CPC98 aurochs sequence reads aligned to individual bovine chromosomes. 6](#_Toc430893437)

[Figure S3. Cumulative distribution of CPC98 insert sizes for read pairs aligning to the bovine reference genome (UMD3.1). 7](#_Toc430893438)

[Figure S4. mapDamage 2.0 analyses of CPC98 sequence reads. 8](#_Toc430893439)

[Figure S5. ML phylogeny constructed for a reduced panel of 278 individuals using 10,923 high-quality SNPs and Bayesian-based support for each branch. 9](#_Toc430893440)

[Figure S6. Unsupervised hierarchical clustering of 1,226 cattle using STRUCTURE. 10](#_Toc430893441)

[Figure S7. TreeMix ML phylogeny constructed using genotype data (15,498 SNPs) from 50 European and American taurine breeds, the single CPC98 aurochs specimen and the Brahman zebu breed. 11](#_Toc430893442)

[Figure S8. TreeMix ML phylogeny constructed using genotype data (15,498 SNPs) from eight European and American regional cattle population groups and the single CPC98 aurochs specimen. 12](#_Toc430893443)

[Figure S9 (pages 13–27). Detection of selective sweeps in the *B. taurus* lineage. 28](#_Toc430893444)

[Figure S10. Alignment of bta-mir-2893 and bpr-mir-2893 sequences. 29](#_Toc430893445)

[Figure S11. Pathway analysis results for bta-mir-2893 and bpr-mir-2893 targets. 30](#_Toc430893446)

[Figure S12. Agarose gel-based removal of Illumina® GA sequencing adaptor sequence from the C1 and C2 CPC98 single read libraries. 31](#_Toc430893447)

Table of Supporting Tables

[Table S1. Number of flow cell lanes used for six CPC98 Aurochs libraries sequenced at three different centres, using two Illumina® sequencing platforms. 32](#_Toc430893413)

[Table S2. Summary of sequencing lane data obtained for six CPC98 Aurochs libraries sequenced at three different centres, using two Illumina^®^ sequencing platforms. 33](#_Toc430893414)

[Table S3. Filtered CPC98 Aurochs sequence reads aligned to chromosomes in the *B. taurus* genome build UMD3.1 categorized by different Phred-scaled mapping quality (MAPQ) scores. 39](#_Toc430893415)

[Table S4. CPC98 Aurochs sequence reads aligned to *Btau* UMD3.1 reference genome chromosomes. 41](#_Toc430893416)

[Table S5. Estimating contamination from modern cattle mtDNA using mtDNA haplotype information and CPC98 Aurochs sequence reads. 43](#_Toc430893417)

[Table S6. Cattle breed and population information for 1,228 animals with Illumina^®^ BovineSNP50 SNP genotype data used for phylogenetic and population genomics analyses. 44](#_Toc430893418)

[Table S7. Mean ABBA/BABA test *D* statistics of admixture with British aurochs (recent outgroup O1) for individual European cattle breeds (P1) estimated using seven West African taurine populations (P2). 49](#_Toc430893419)

[Table S8. Mean jackknifed *D* statistics (lower triangular matrix) and standard errors (upper triangular matrix) for pairwise comparisons between cattle breeds from different geographical regions. 52](#_Toc430893420)

[Table S9. Ensembl VEP (build 78) predicted effects for 263 SNPs and indels potentially selected post-domestication in European *B. taurus* cattle. 53](#_Toc430893421)

[Table S10. Details of eight missense mutations potentially selected post-domestication in European *B.* *taurus* cattle. 54](#_Toc430893422)

[Table S11. Top 25-ranking IPA gene categories enriched for 166 out of 193 genes associated with post-domestic DNA sequence variants (VEP analysis) that mapped to molecules in the IPA Knowledge Base (see also Additional File 2). 55](#_Toc430893423)

[Table S12. The 16 top-ranking significant IPA canonical pathways (*P* ≤ 0.05) enriched for 166 out of 193 genes associated with post-domestic DNA sequence variants (VEP analysis) that mapped to molecules in the IPA Knowledge Base (see also Additional File 2). 58](#_Toc430893424)

[Table S13. Genes within genomic windows with evidence for positive selection in the *B. taurus* lineage identified using the HKA test (colour-coding highlights uncorrected *P*-value significance thresholds). 59](#_Toc430893425)

[Table S14. Top 25-ranking IPA gene categories enriched for 96 unique genes mapping to IPA Knowledge Base molecules and within genomic windows under positive selection in the *B. taurus* lineage according to the HKA analysis. 68](#_Toc430893426)

[Table S15. The top 25-ranking IPA canonical pathways enriched for 96 unique genes mapping to IPA Knowledge Base molecules and within genomic windows under positive selection in the *B. taurus* lineage according to the HKA analysis. 71](#_Toc430893427)

[Table S16. Number of genes targeted by the five miRNAs containing polymorphisms (in the mature miRNA sequence) between *B. primigenius* and *B. taurus.* 73](#_Toc430893428)

[Table S17. *B. taurus* genes selected for Agilent SureSelect target enrichment. 74](#_Toc430893429)

Supporting Figures


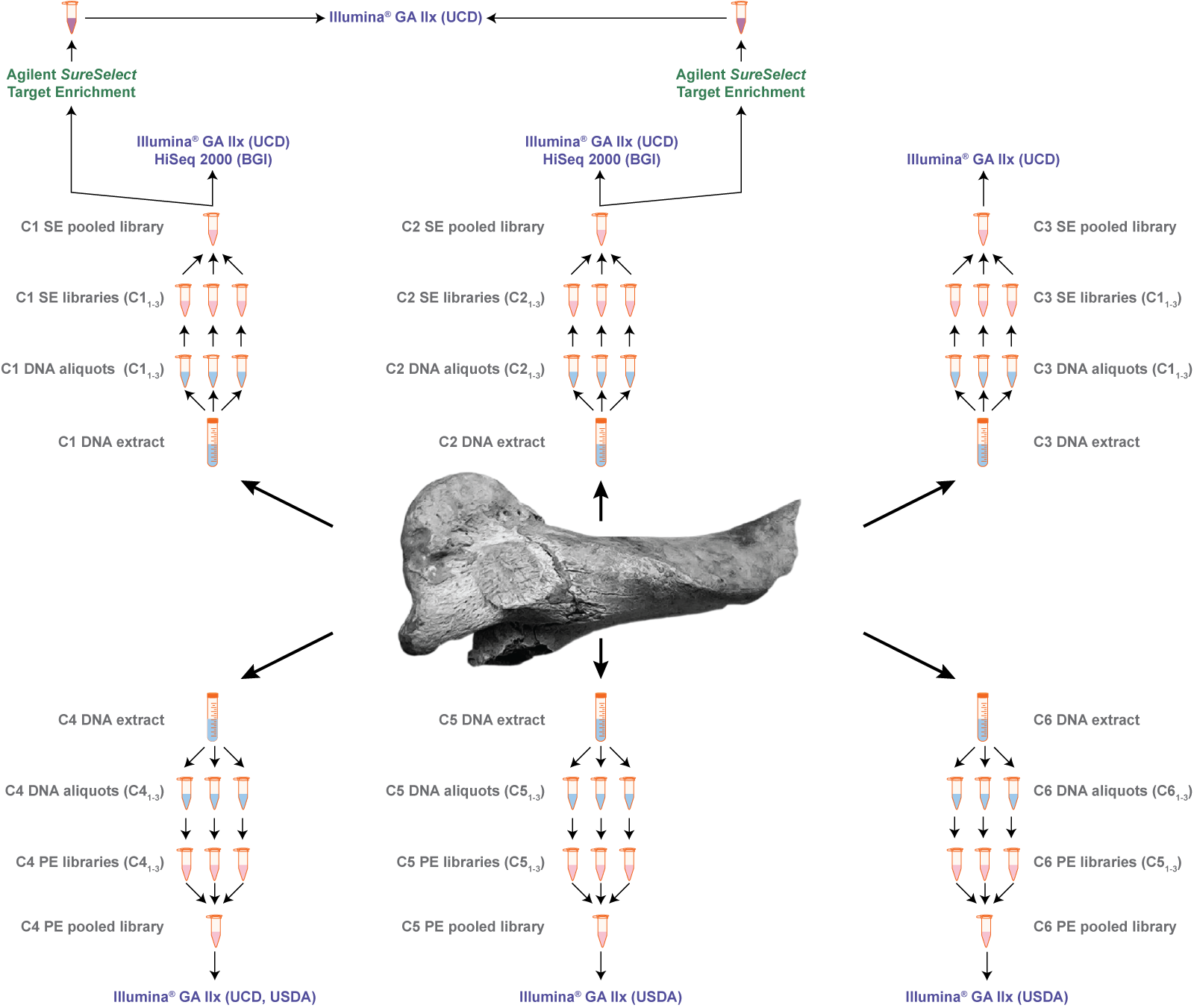


Figure S1. Schematic of the DNA extracts and Illumina® single read and paired-end sequencing libraries used for the generation of the CPC98 genome sequence.

Sequencing centres used for this study are indicated (BGI, UCD or USDA). The single-end libraries C1 and C2 were also used for Agilent SureSelect target enrichment followed by single read sequencing.


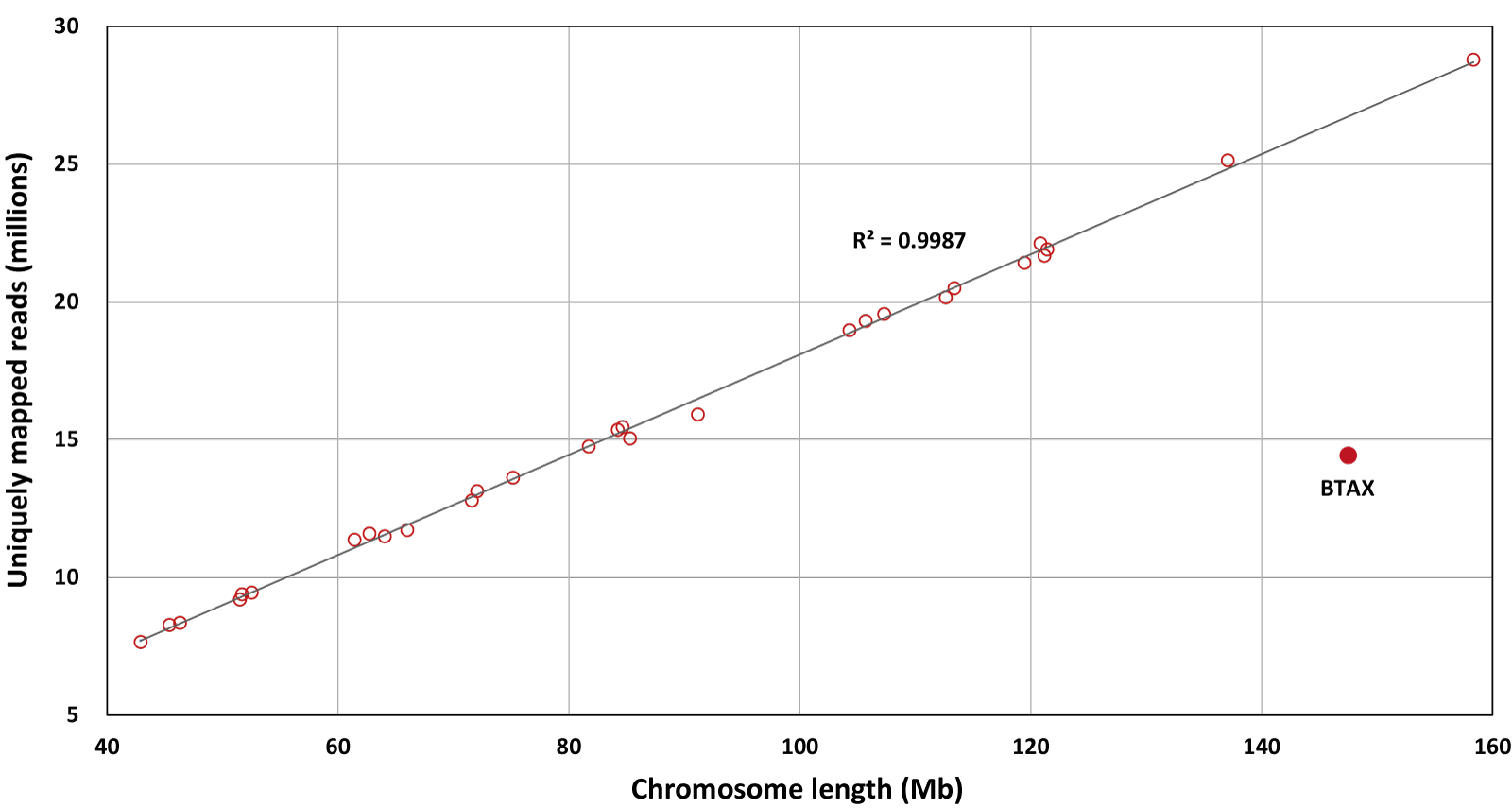


Figure S2. CPC98 aurochs sequence reads aligned to individual bovine chromosomes.

Uniform representation of autosomes by size is evident and the density of reads mapping to the X chromosome is approximately 50% of the autosomal read density, demonstrating that the CPC98 bone specimen is from a male animal.


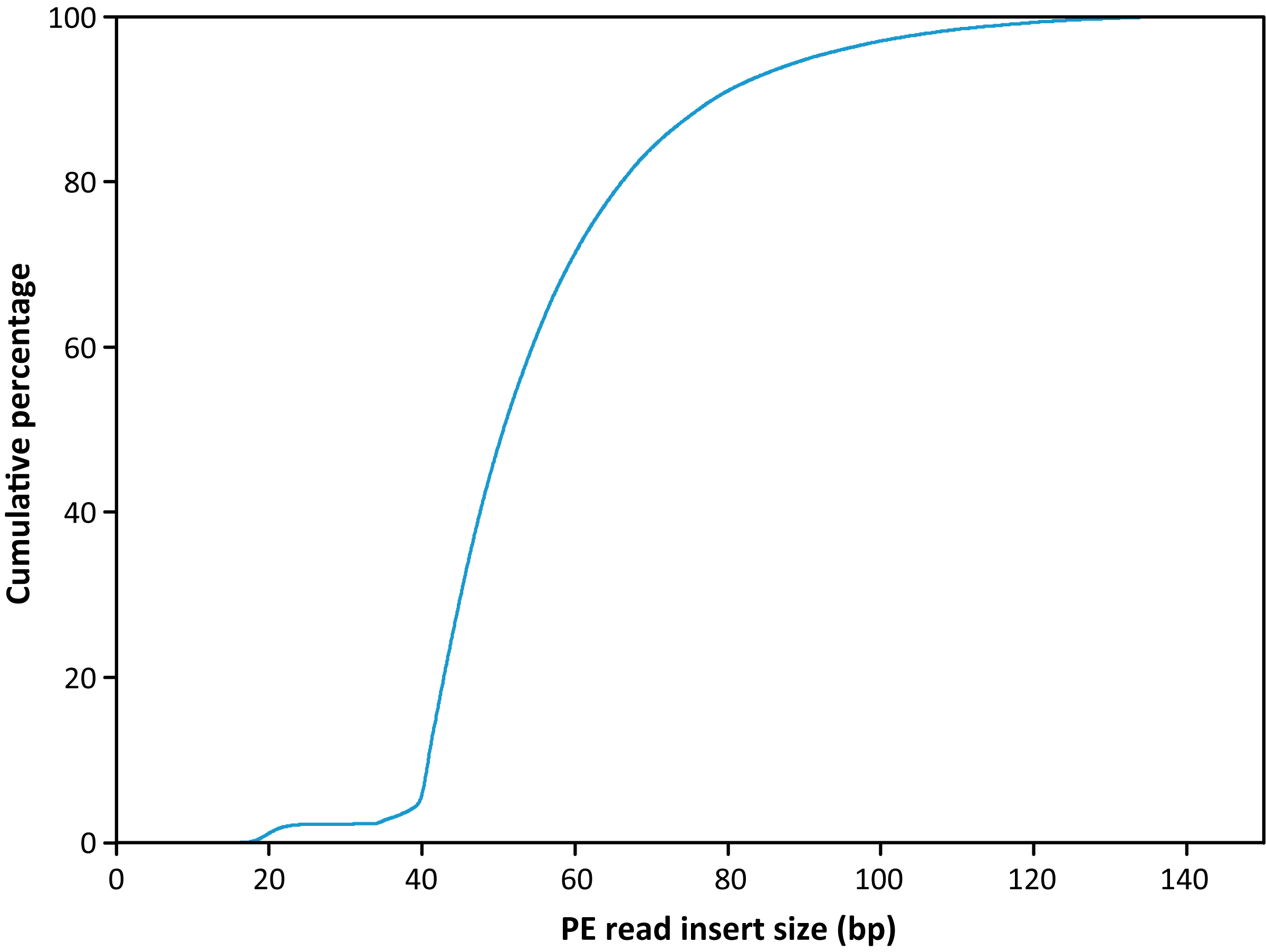


Figure S3. Cumulative distribution of CPC98 insert sizes for read pairs aligning to the bovine reference genome (UMD3.1).

Analysis was performed using 17.8 million high-quality (MAPQ scores ≥ 30) PE read pairs from the C4-C6 libraries. The median CPC98 DNA insert size was estimated at 50 bp, with 99.99% of read pairs having an insert size between 16-150 bp.


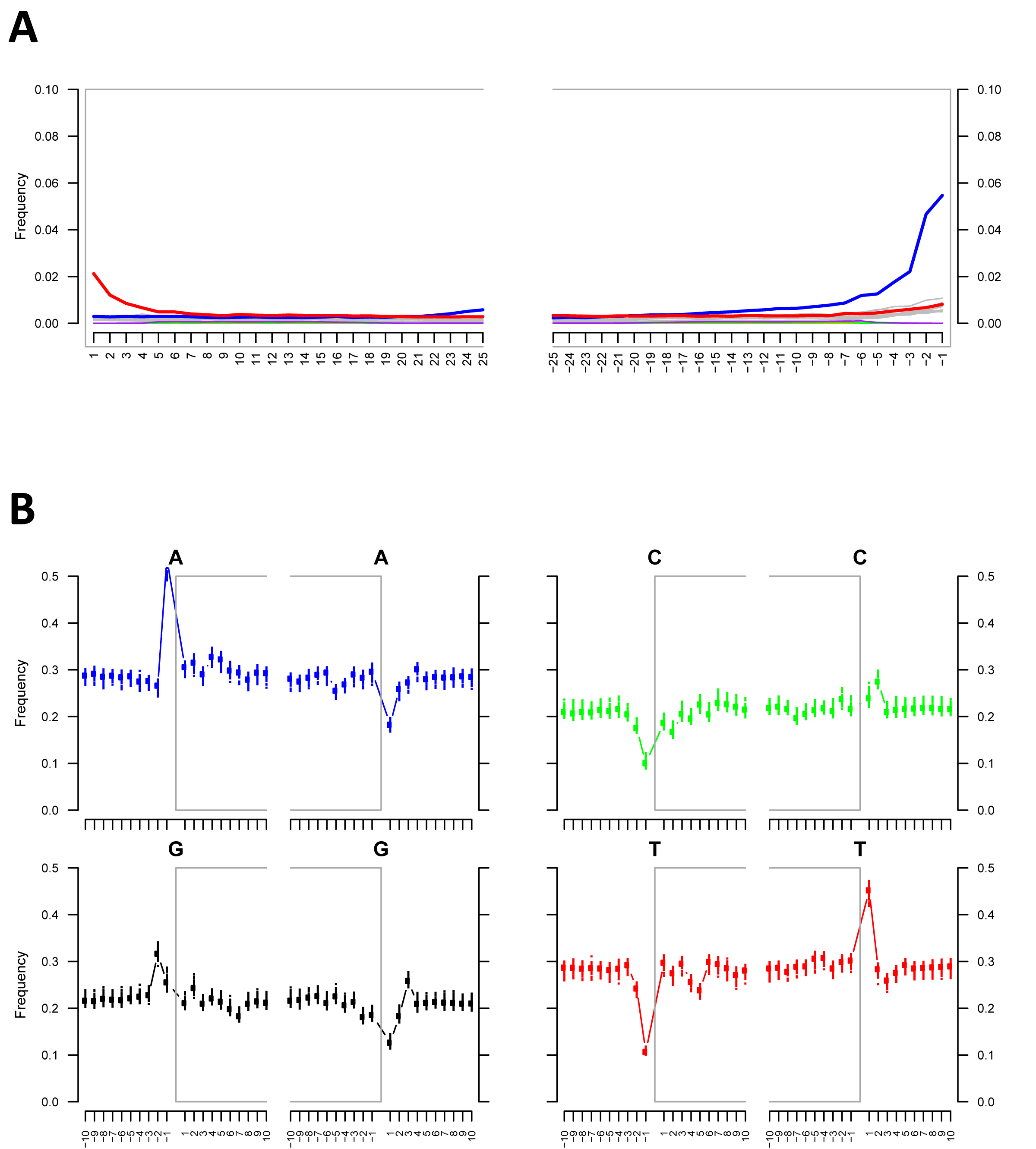


Figure S4. mapDamage 2.0 analyses of CPC98 sequence reads.

One percent (4.7 million) of the uniquely mapped reads were subsetted from the CPC98 aurochs genome alignment. A) Nucleotide misincorporation patterns: positions 1 to 25 from the 5' and 3' ends of reads are shown and an aDNA-characteristic increase in C→T (red) and G→A transitions (blue) was observed for the CPC98 sequence reads. B) Nucleotide frequencies of the aligned reference sequence: the aDNA characteristic enrichment in purines at genomic positions preceding read starts is evident for CPC98 sequence reads.


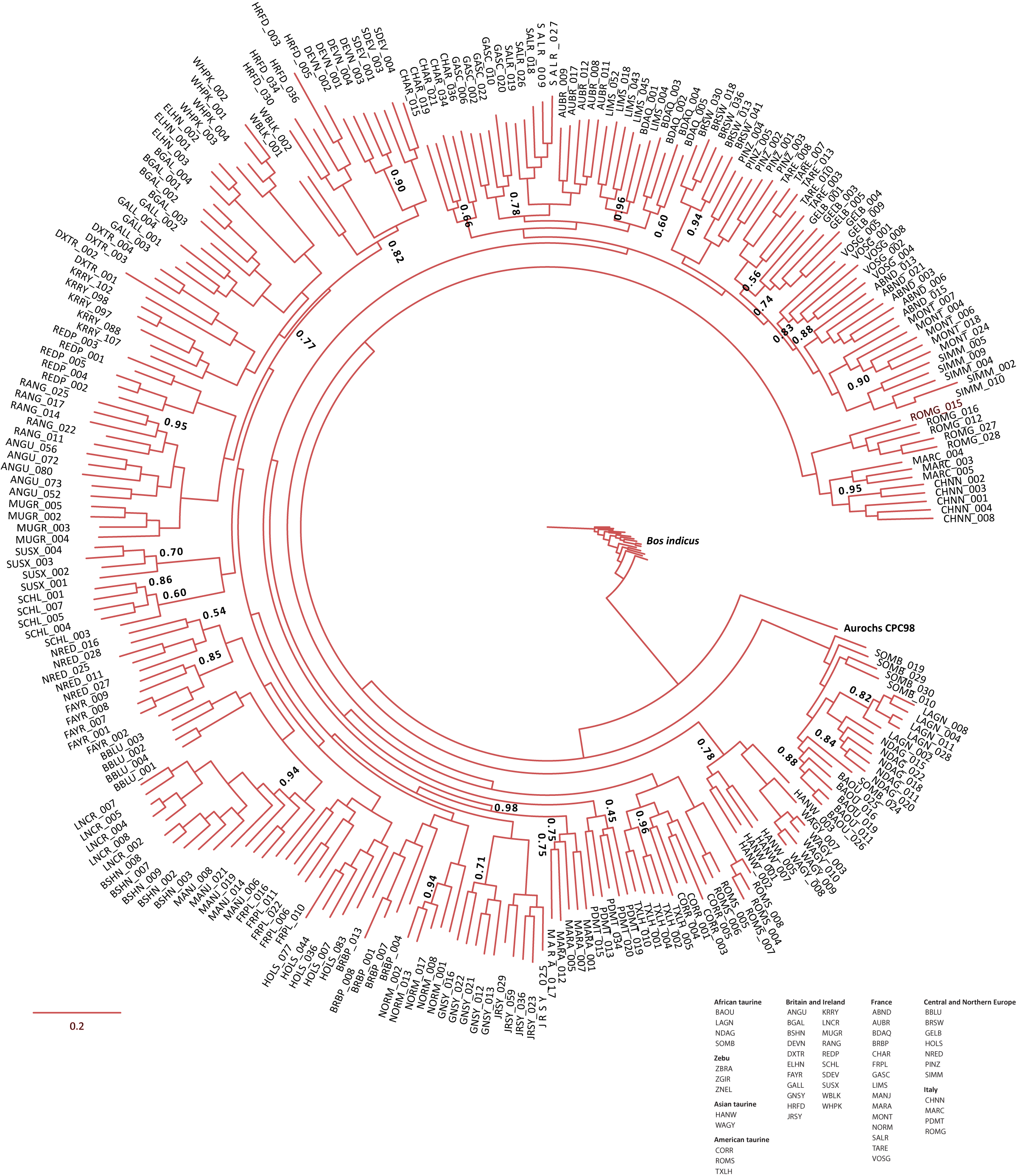


Figure S5. ML phylogeny constructed for a reduced panel of 278 individuals using 10,923 high-quality SNPs and Bayesian-based support for each branch.

Only those branches that gave a Bayesian-based support < 0.99 are labelled; all other branches yielded a Bayesian-based support ≥ 0.99. The branch separating *B. indicus* from all other cattle and the branch separating CPC98 aurochs and the *B. taurus* group both exhibited Bayesian-based supports of 1.00. Evolutionary distances for this tree are based on the model of DNA sequence evolution described previously by Gascuel [1].


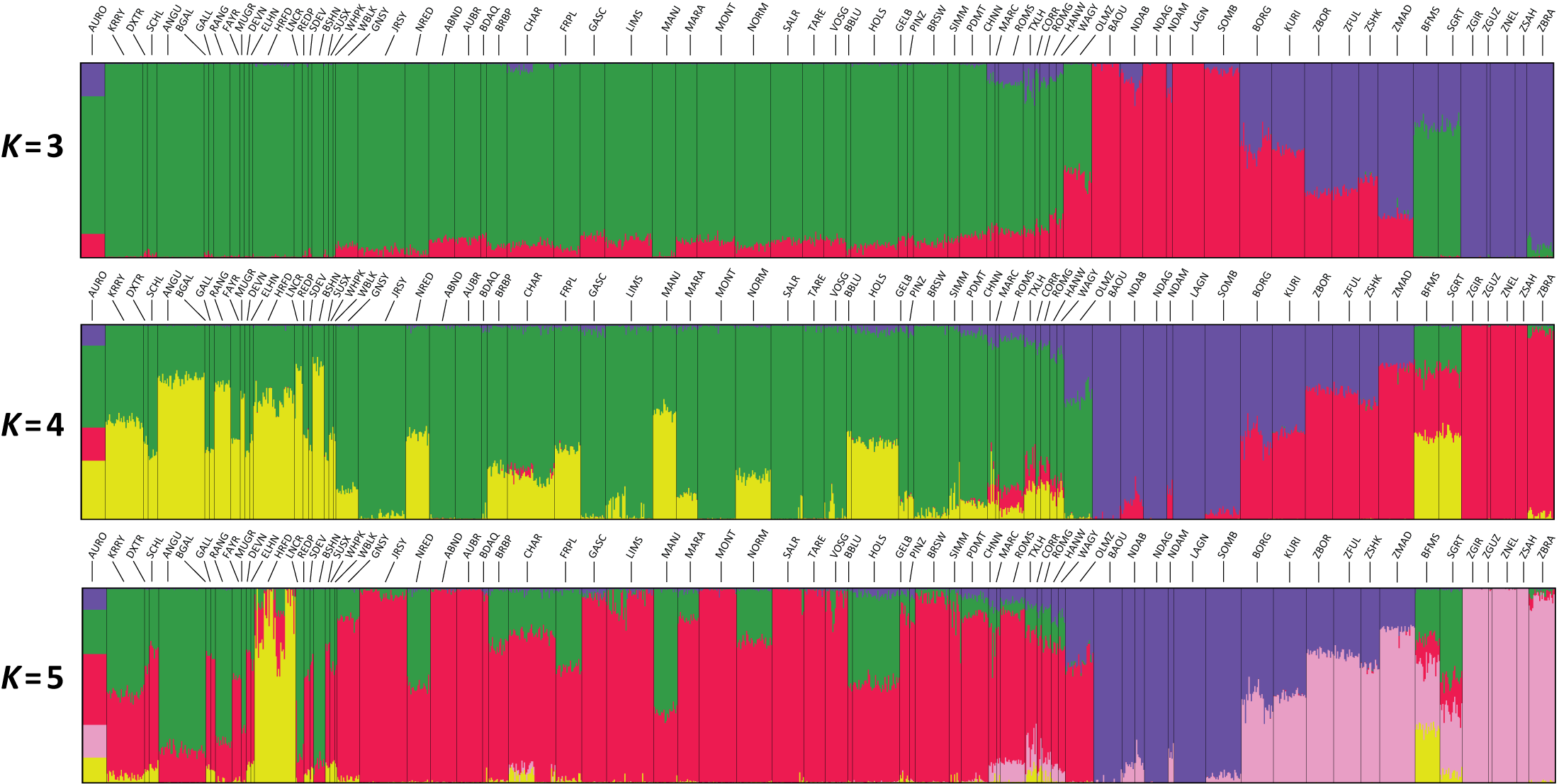


Figure S6. Unsupervised hierarchical clustering of 1,226 cattle using STRUCTURE.

The data set comprises 73 populations, where the African N’Dama taurine cattle from Burkina Faso were considered as two distinct populations. Genotype data for 7,749 SNPs was used and separate results for an inferred number of clusters *K* = 3 to 5 are shown.


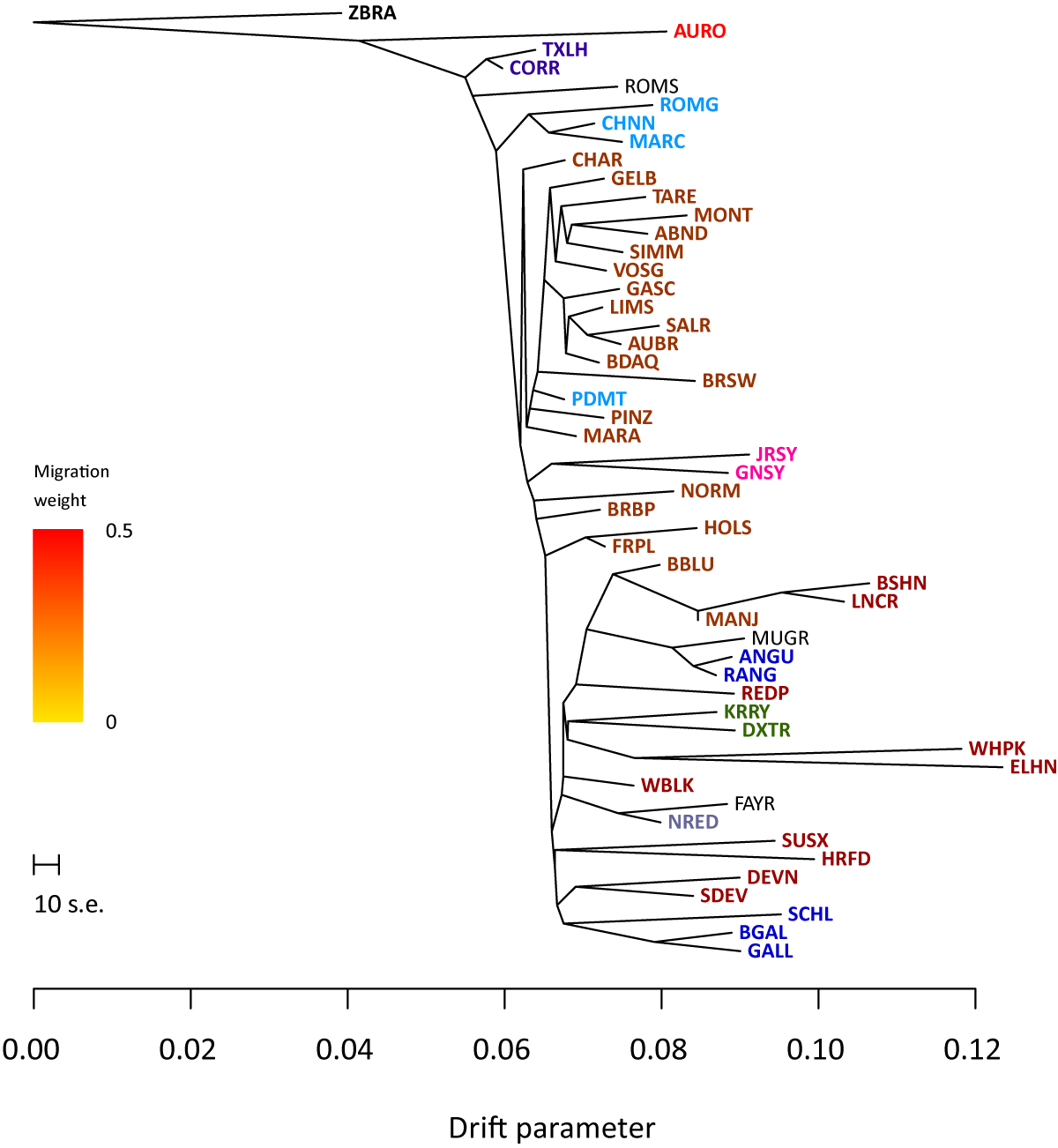


Figure S7. TreeMix ML phylogeny constructed using genotype data (15,498 SNPs) from 50 European and American taurine breeds, the single CPC98 aurochs specimen and the Brahman zebu breed.

This phylogeny was generated with no migration edges to illustrate the base phylogeny of European breeds and the CPC98 aurochs specimen using a *B. indicus* population as an outgroup. The colour coding corresponds to the regional breed grouping shown in **Figures S8** and **Figure 6**. The scale bar shows 10 times the mean standard error of the estimated entries in the sample covariance matrix.


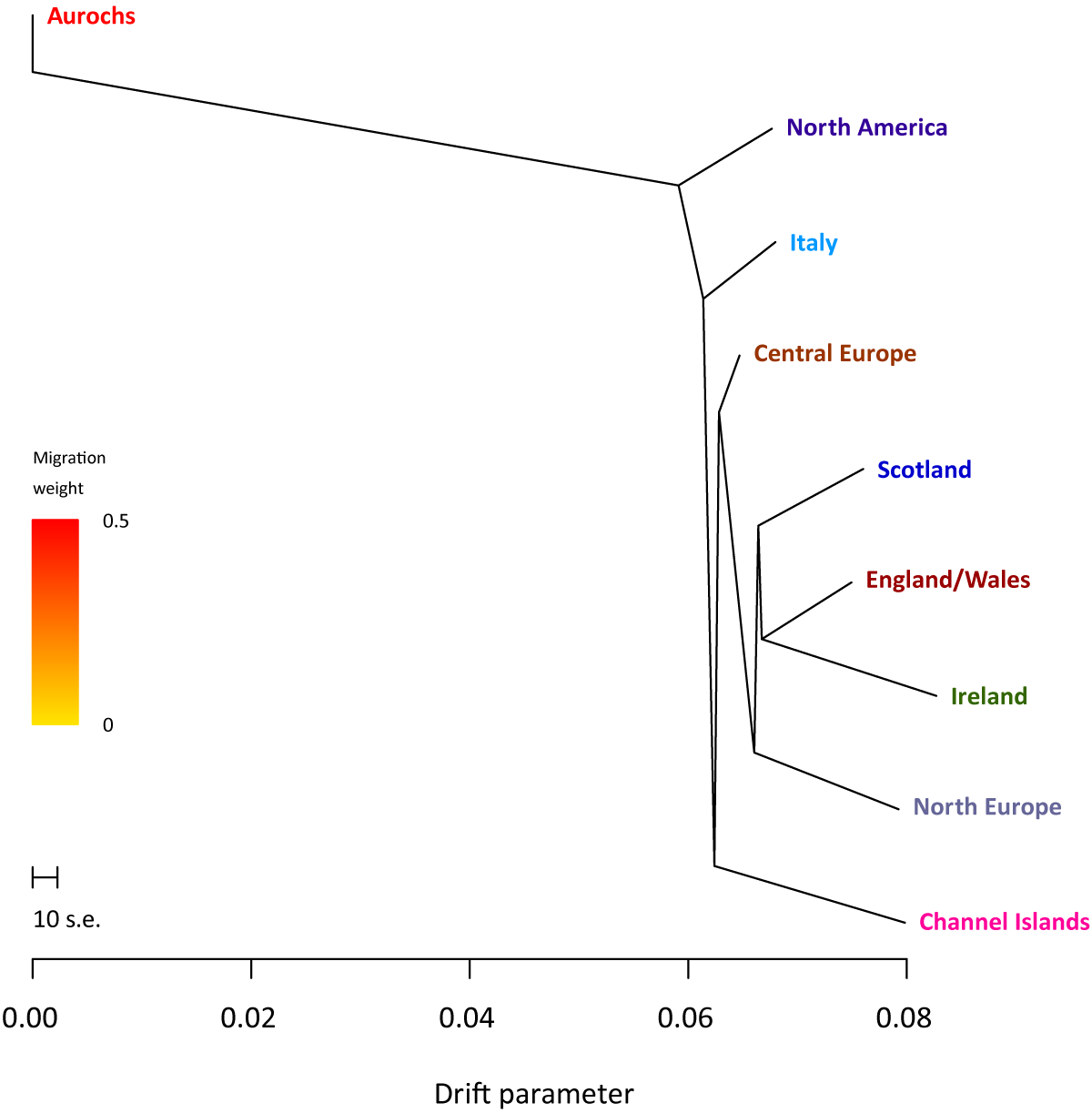


Figure S8. TreeMix ML phylogeny constructed using genotype data (15,498 SNPs) from eight European and American regional cattle population groups and the single CPC98 aurochs specimen.

This phylogeny was generated with no migration edges to demonstrate the base phylogeny of the regional taurine cattle groups with the CPC98 aurochs specimen as an outgroup. The scale bar shows 10 times the mean standard error of the estimated entries in the sample covariance matrix.


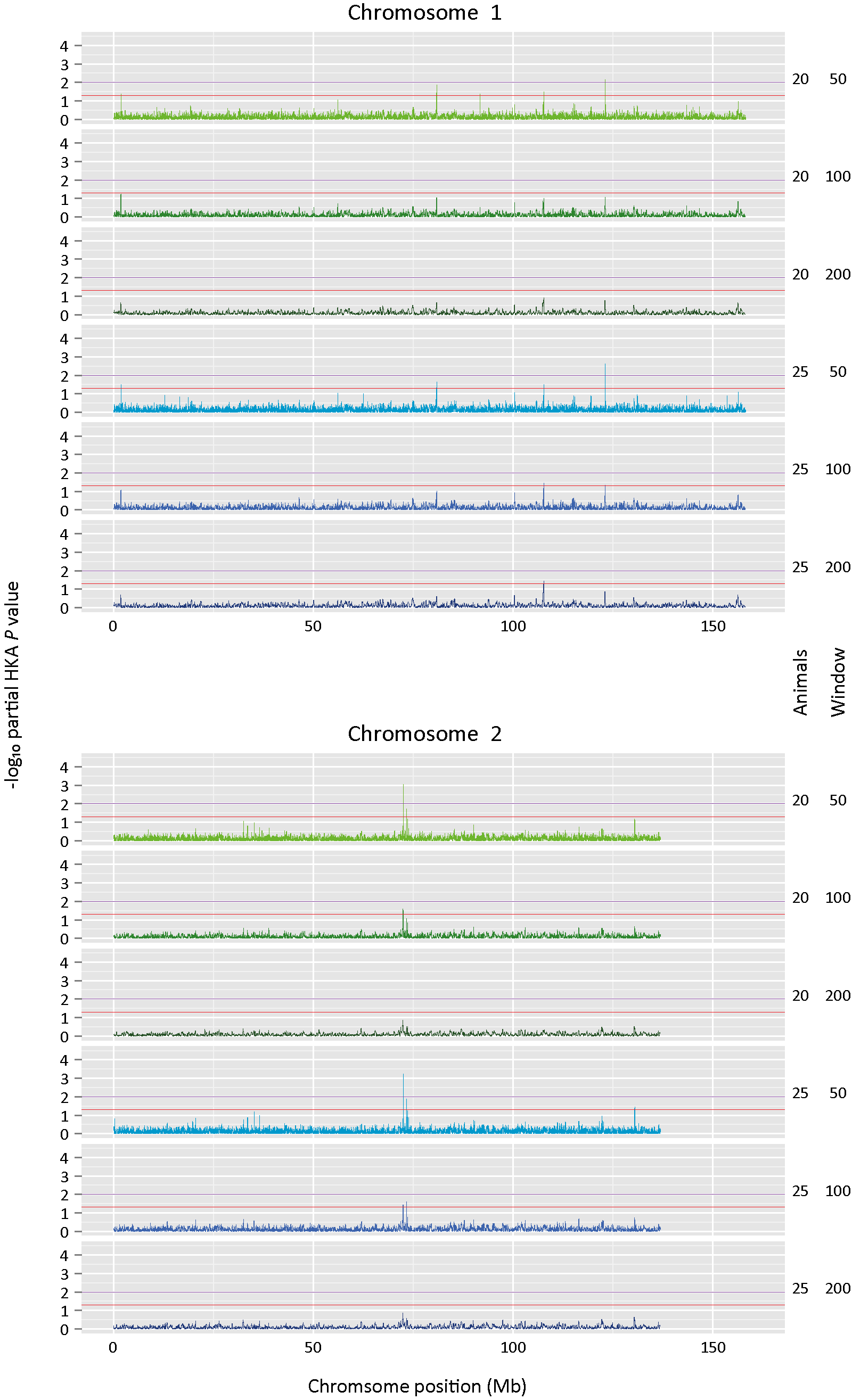

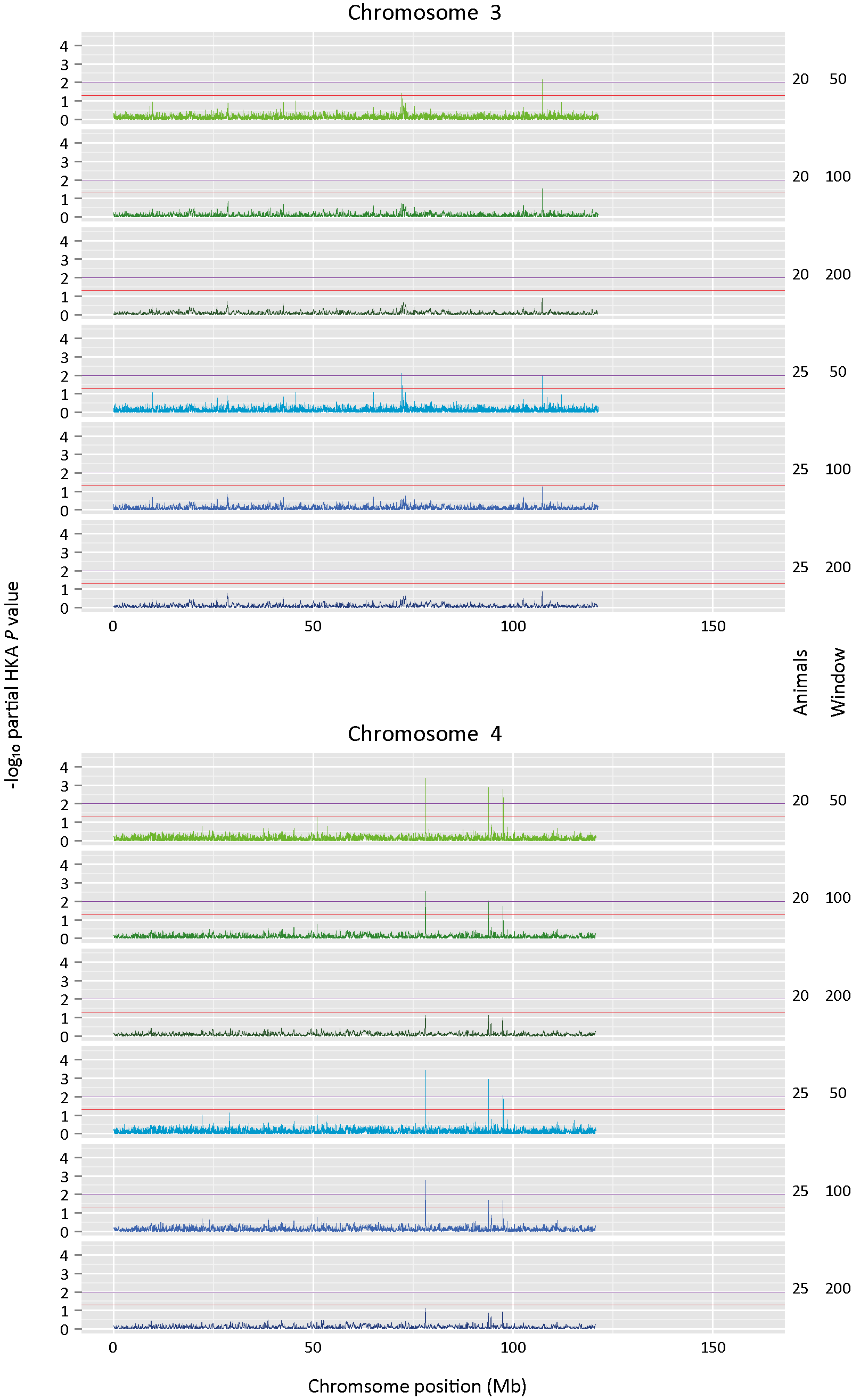

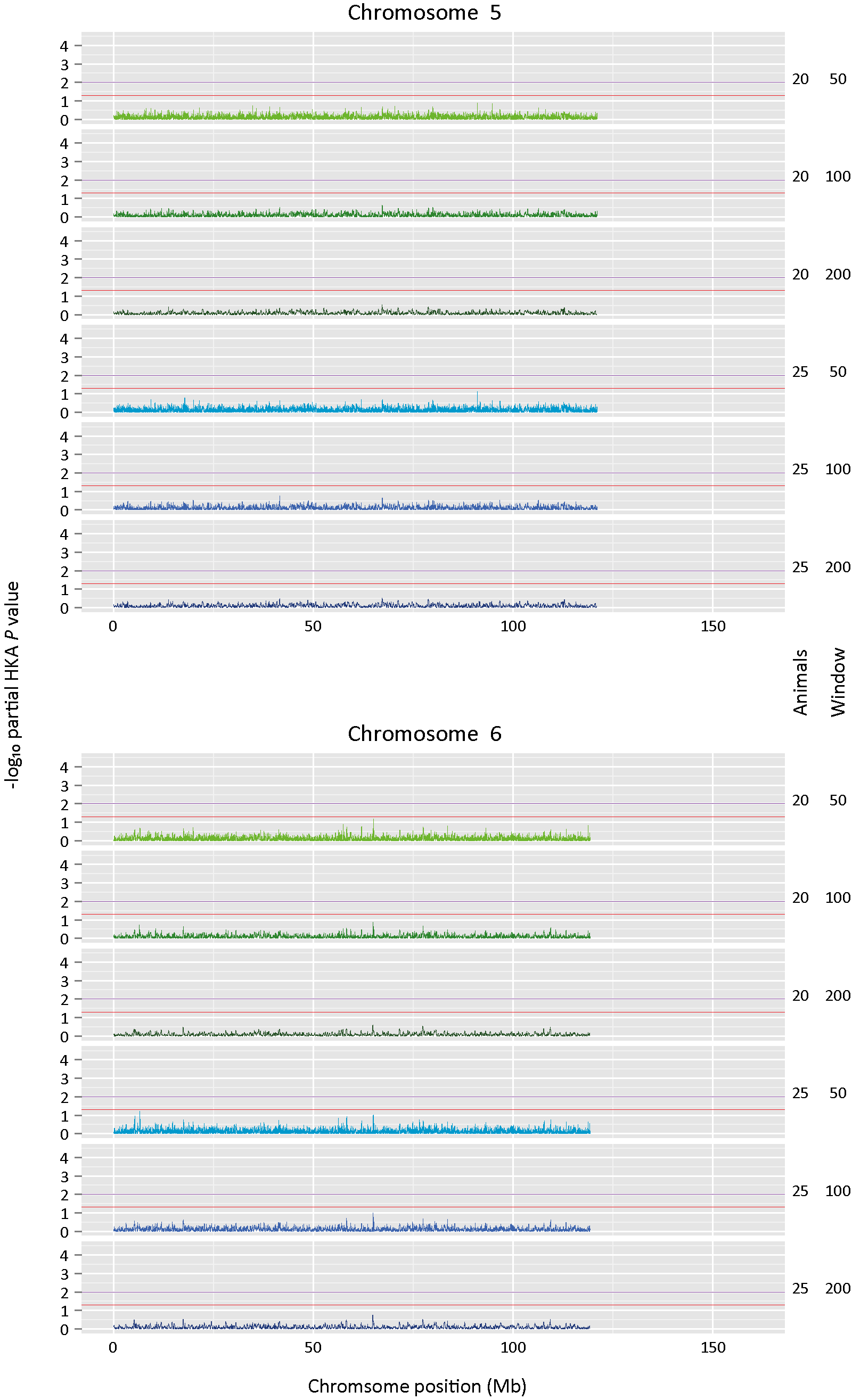

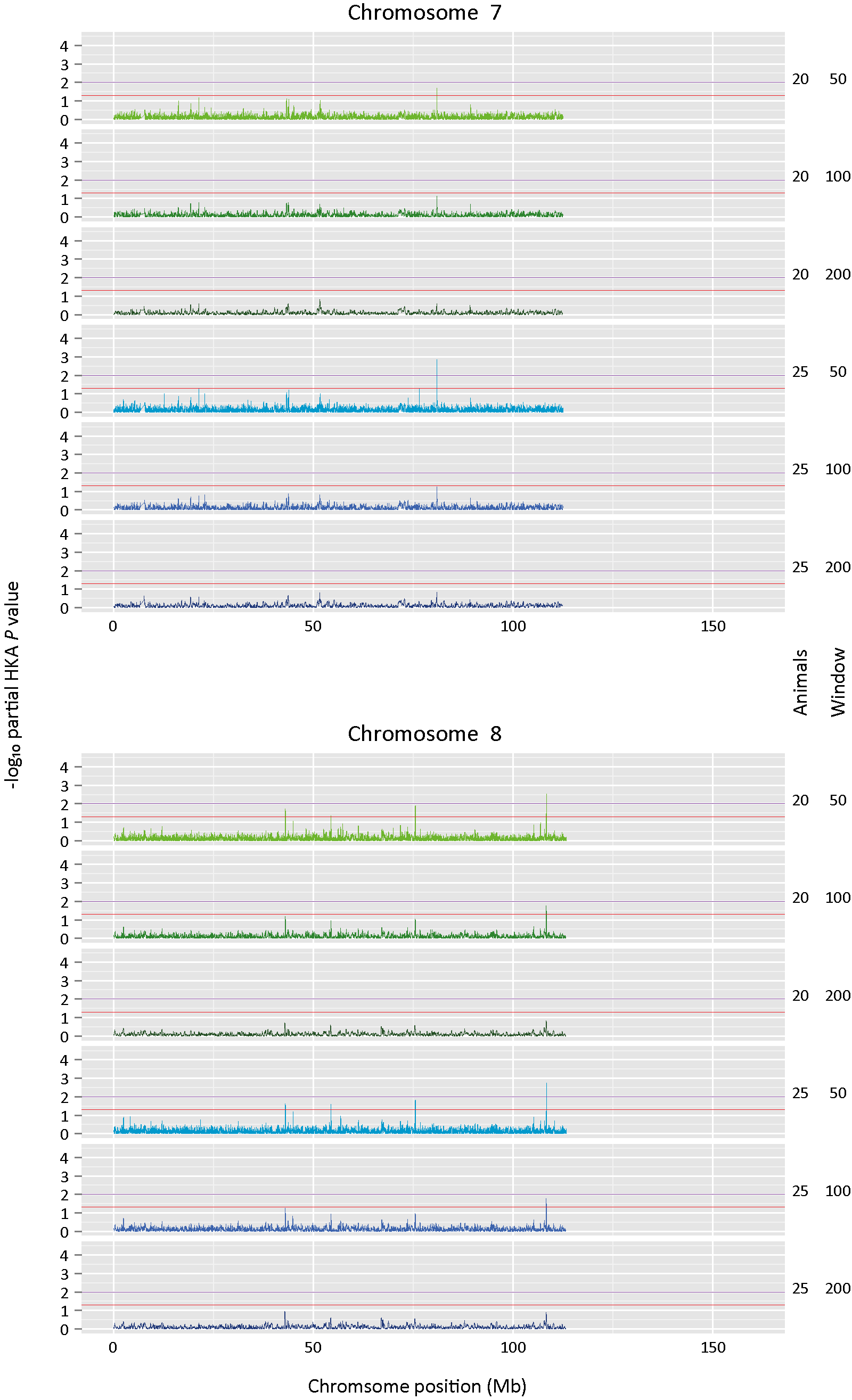

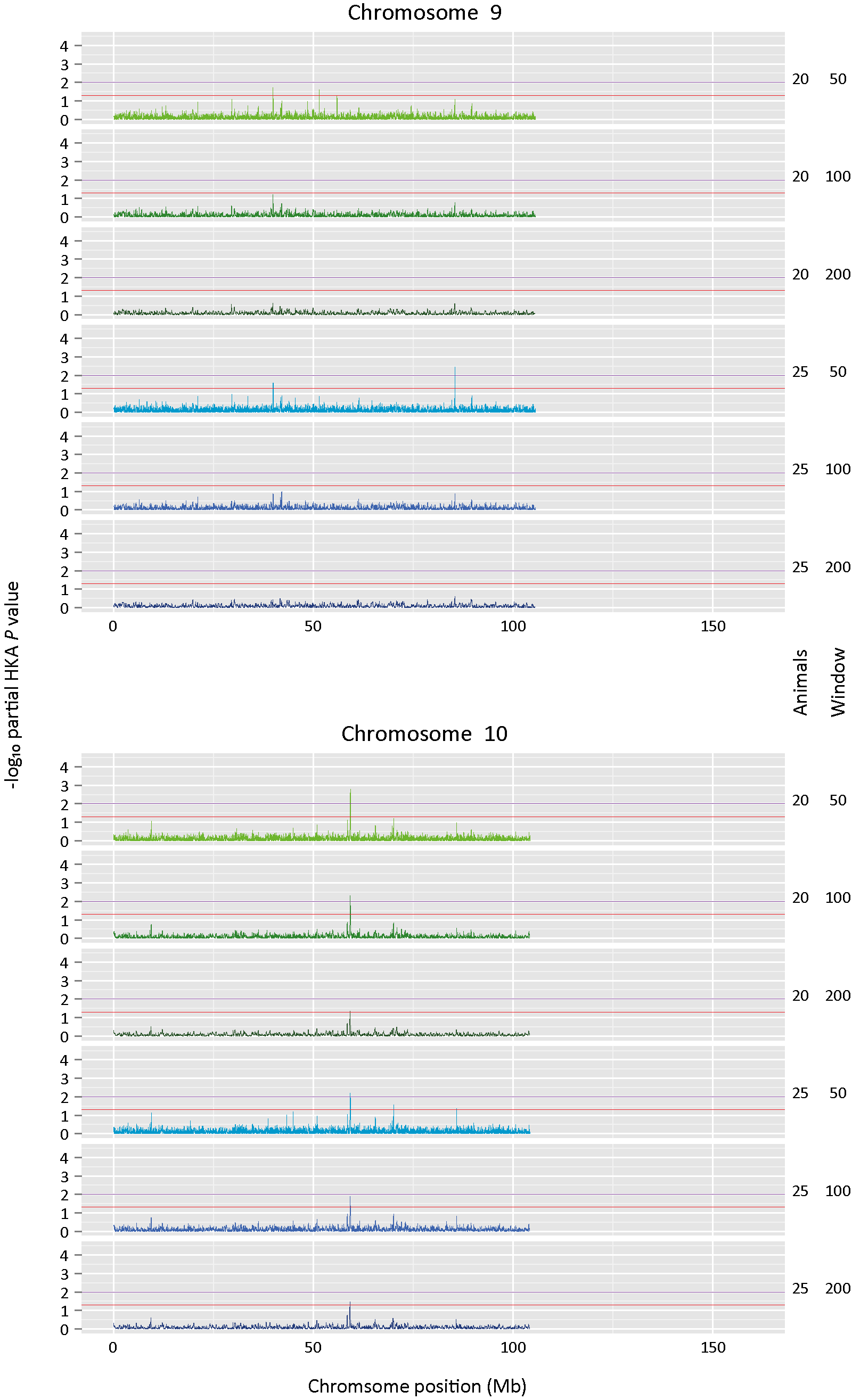

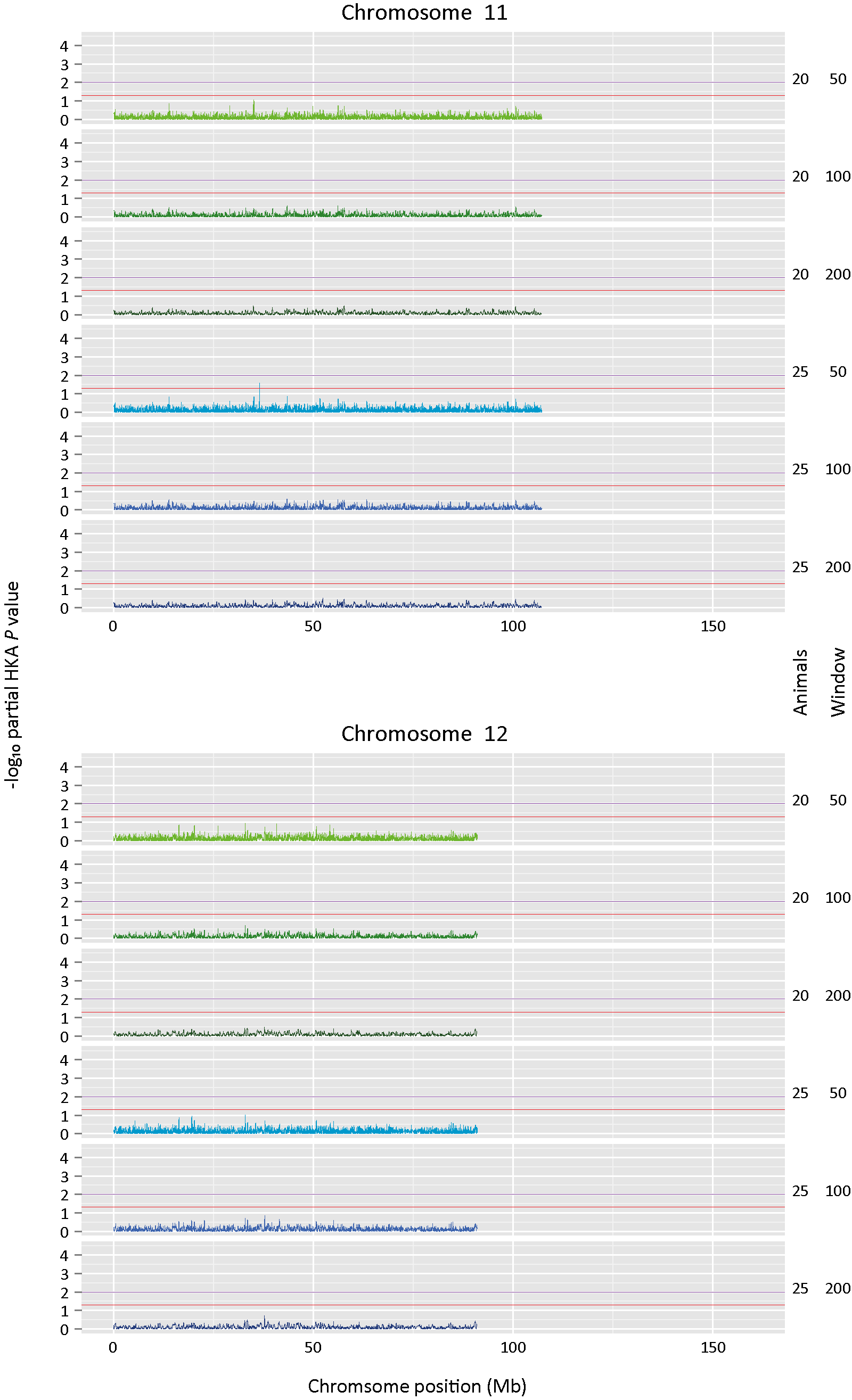

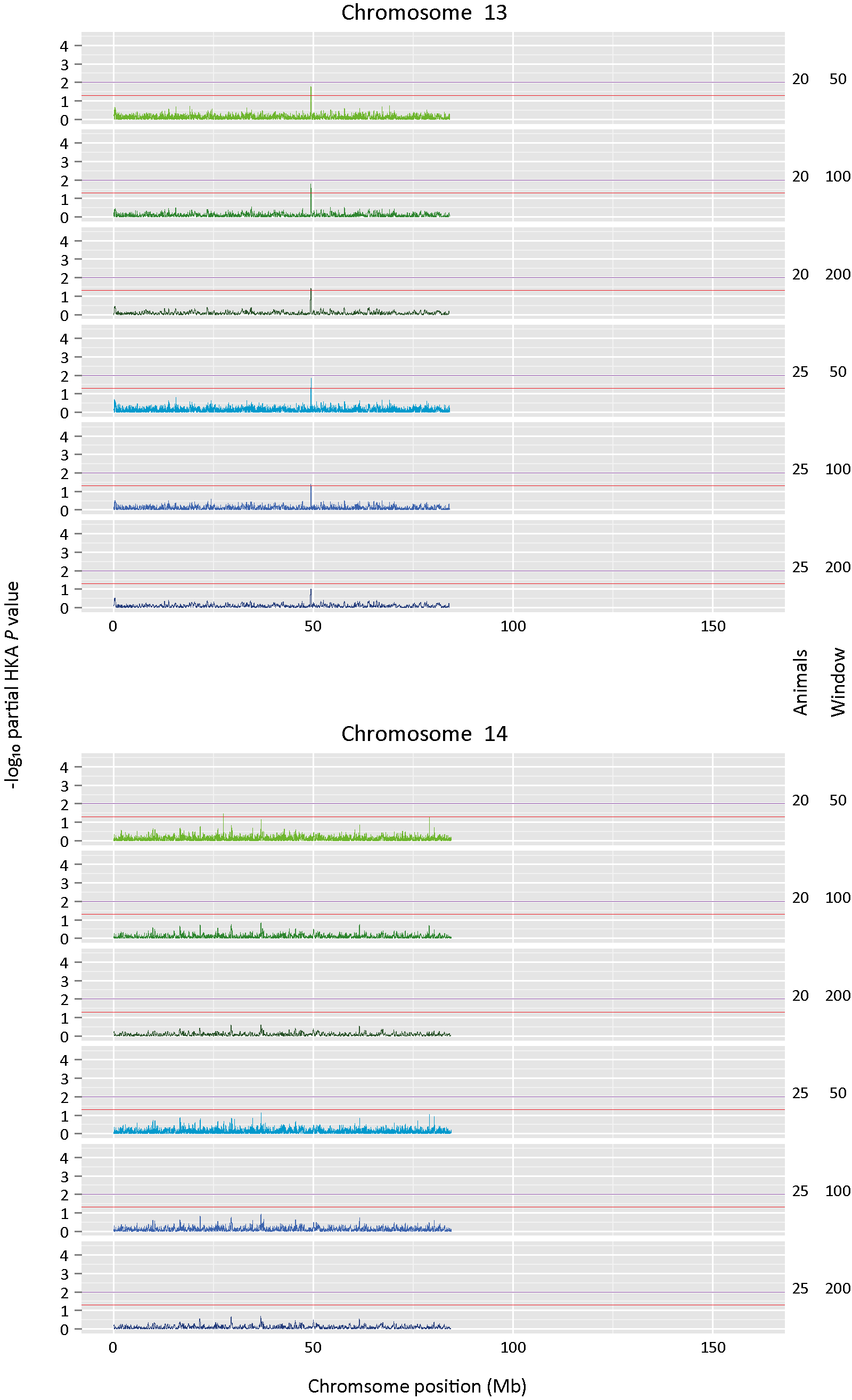

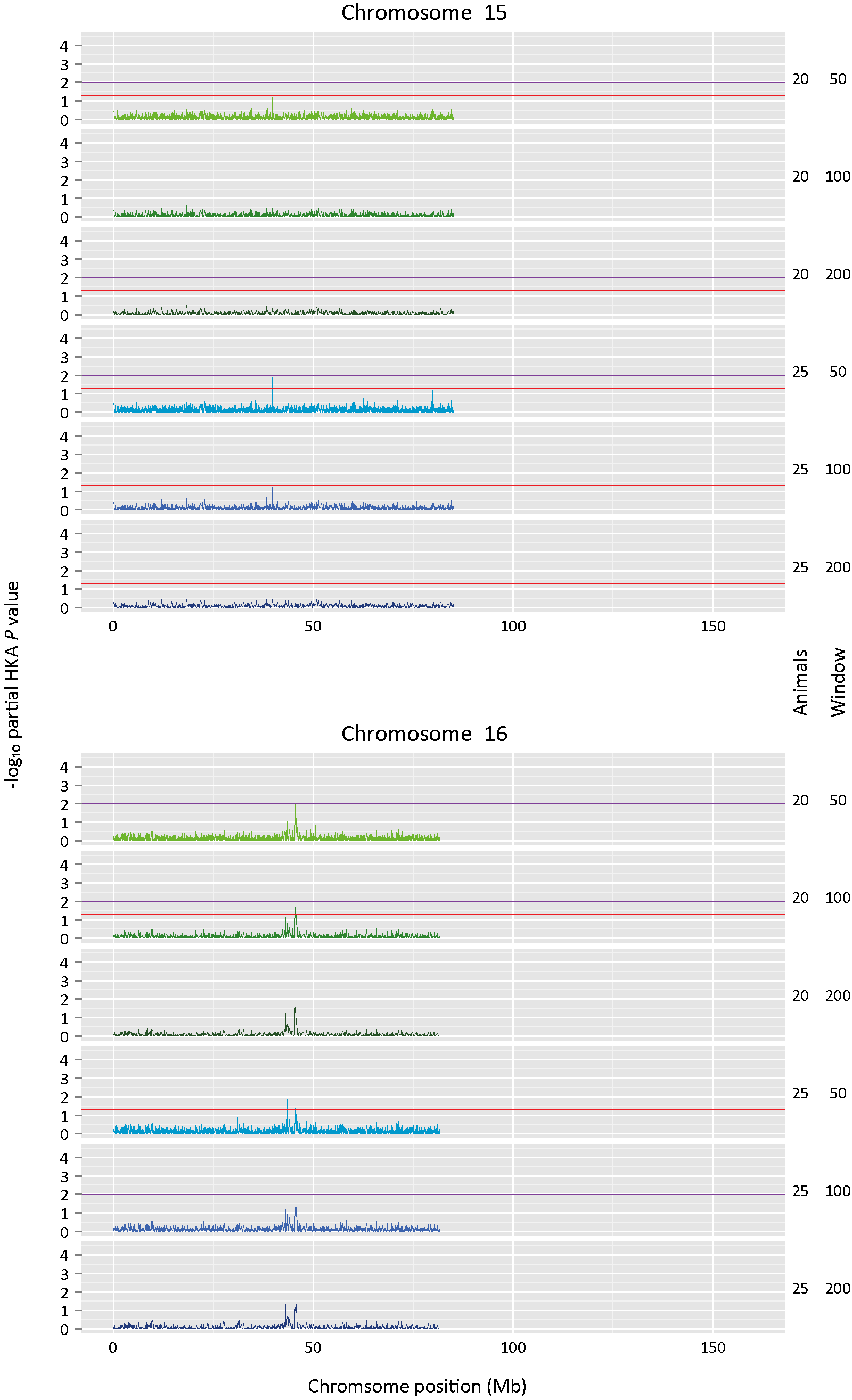

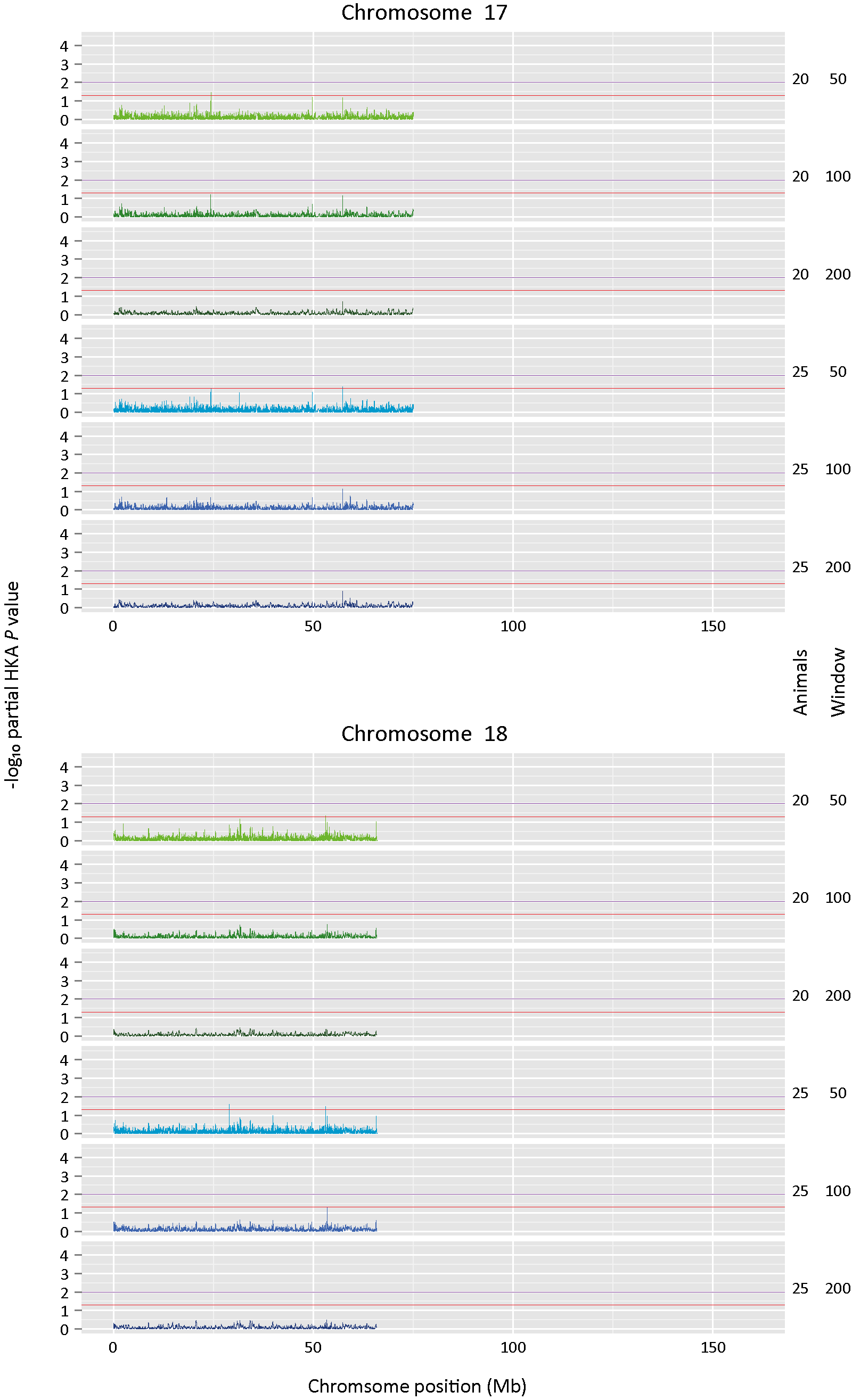

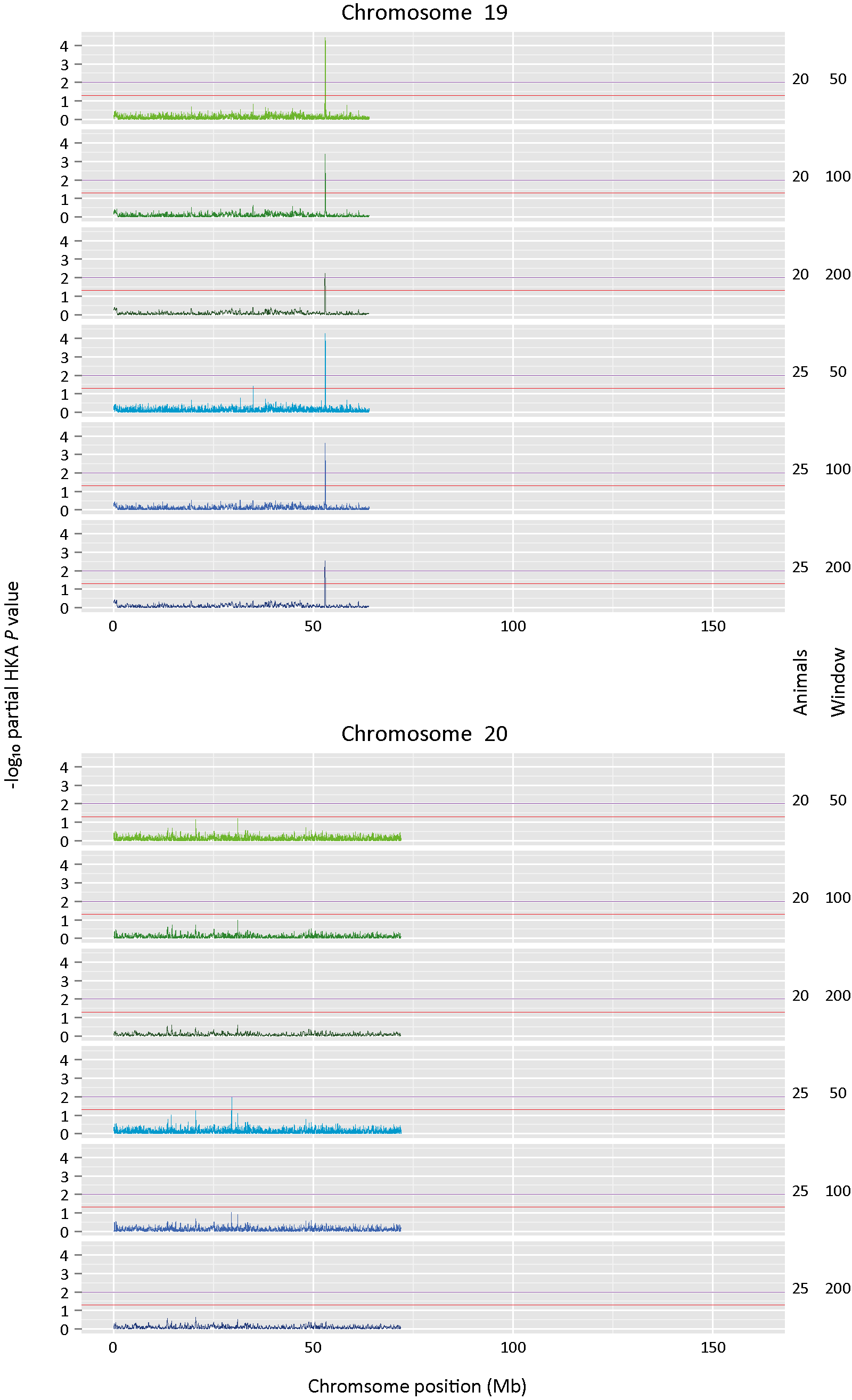

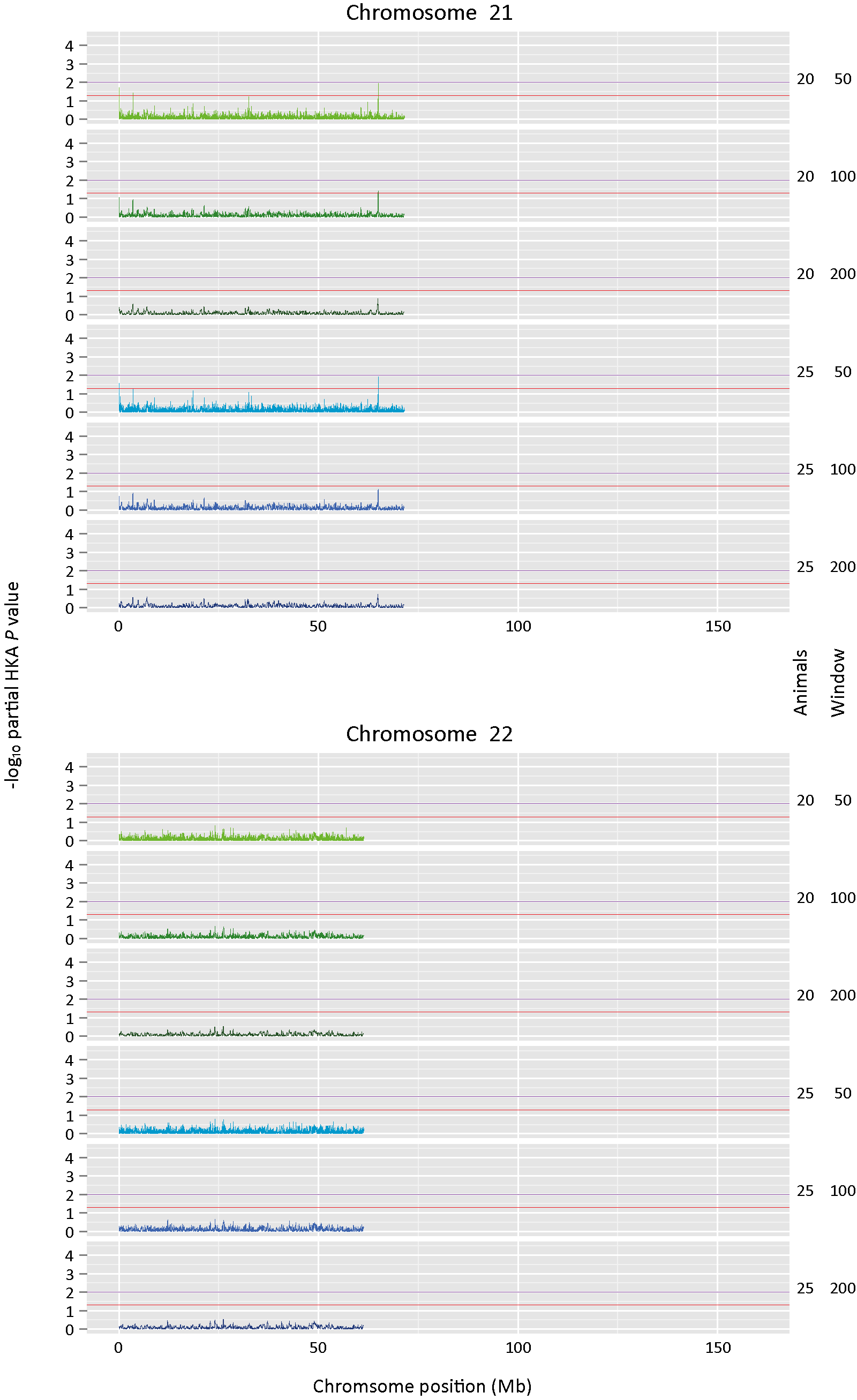

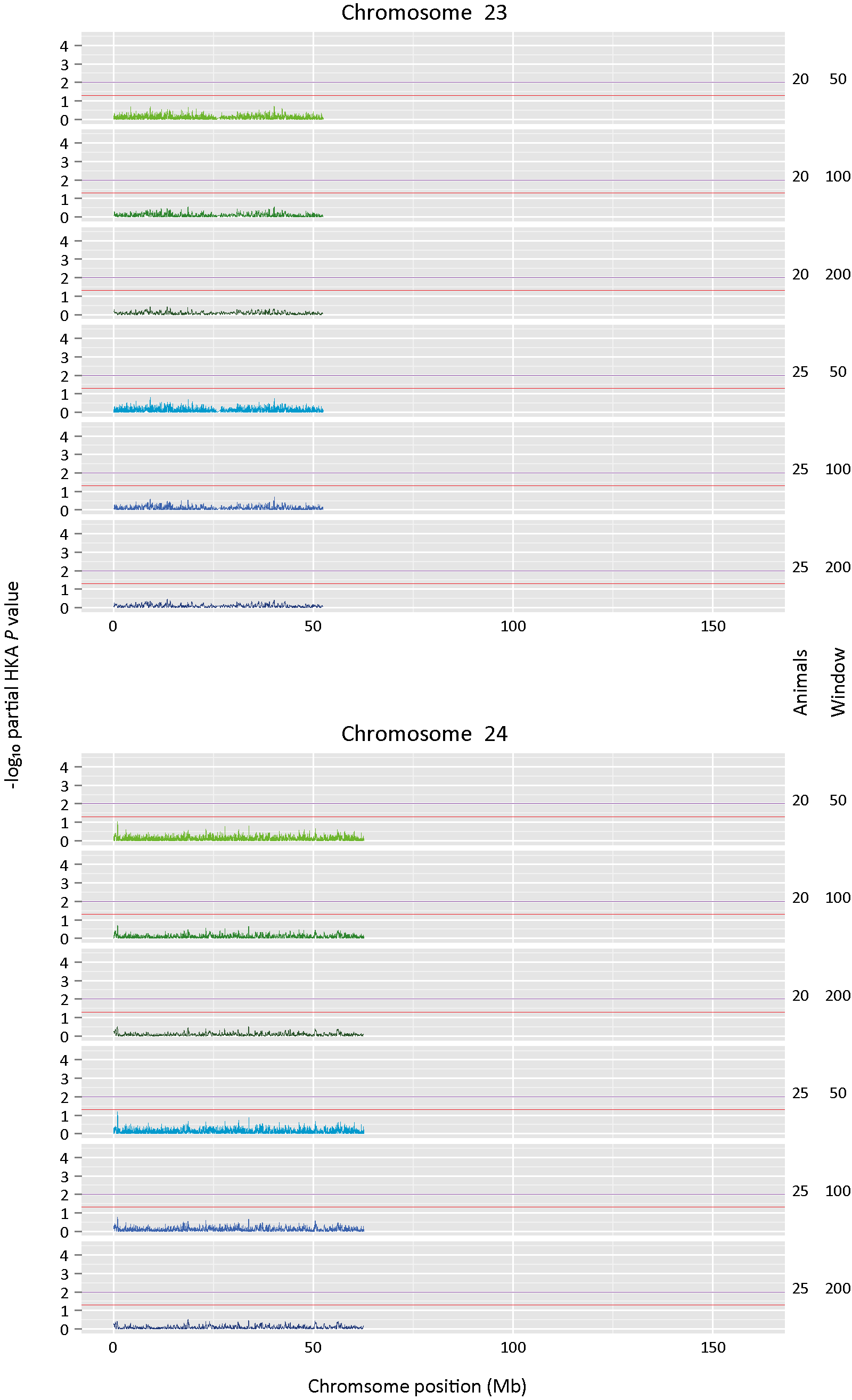

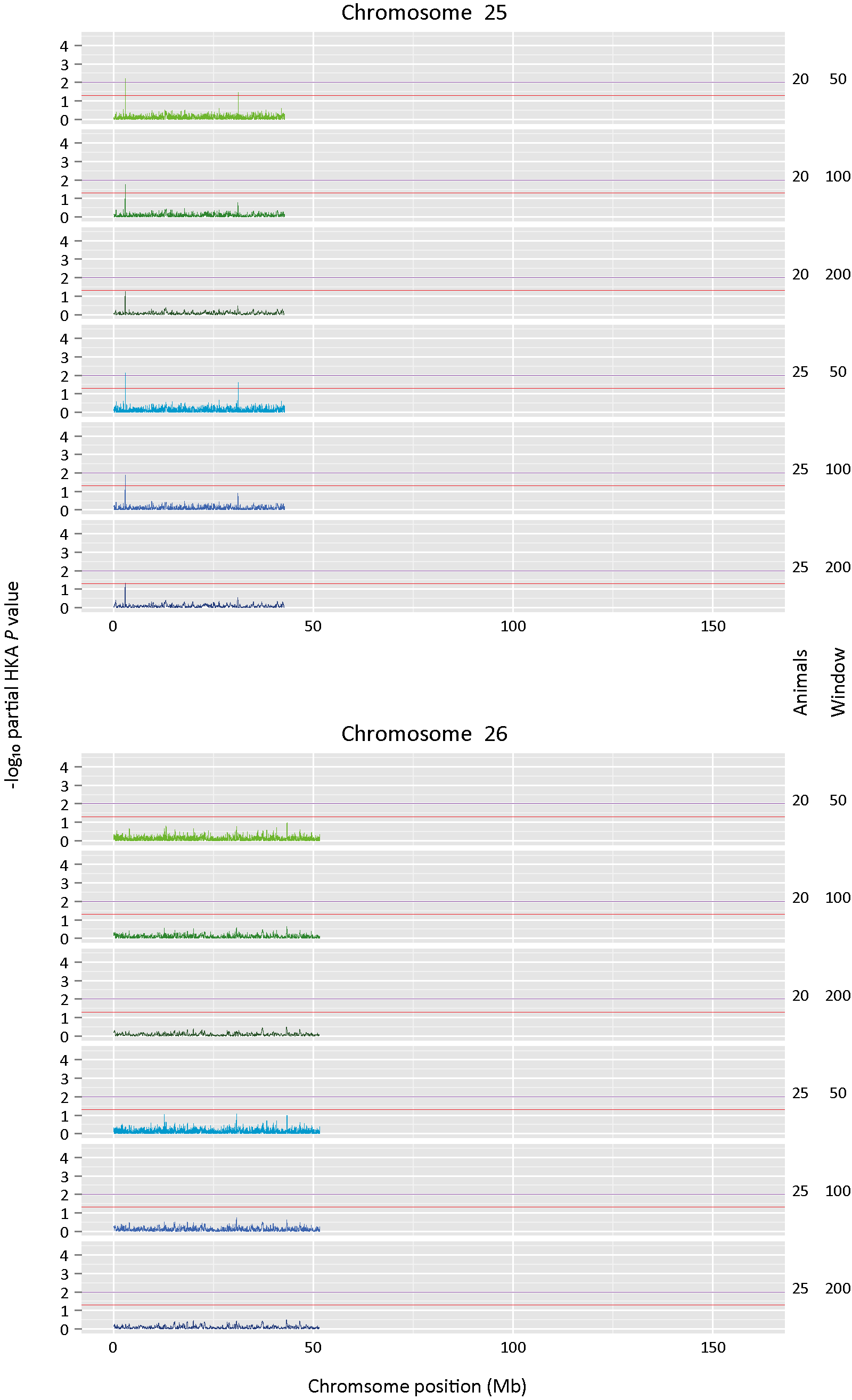

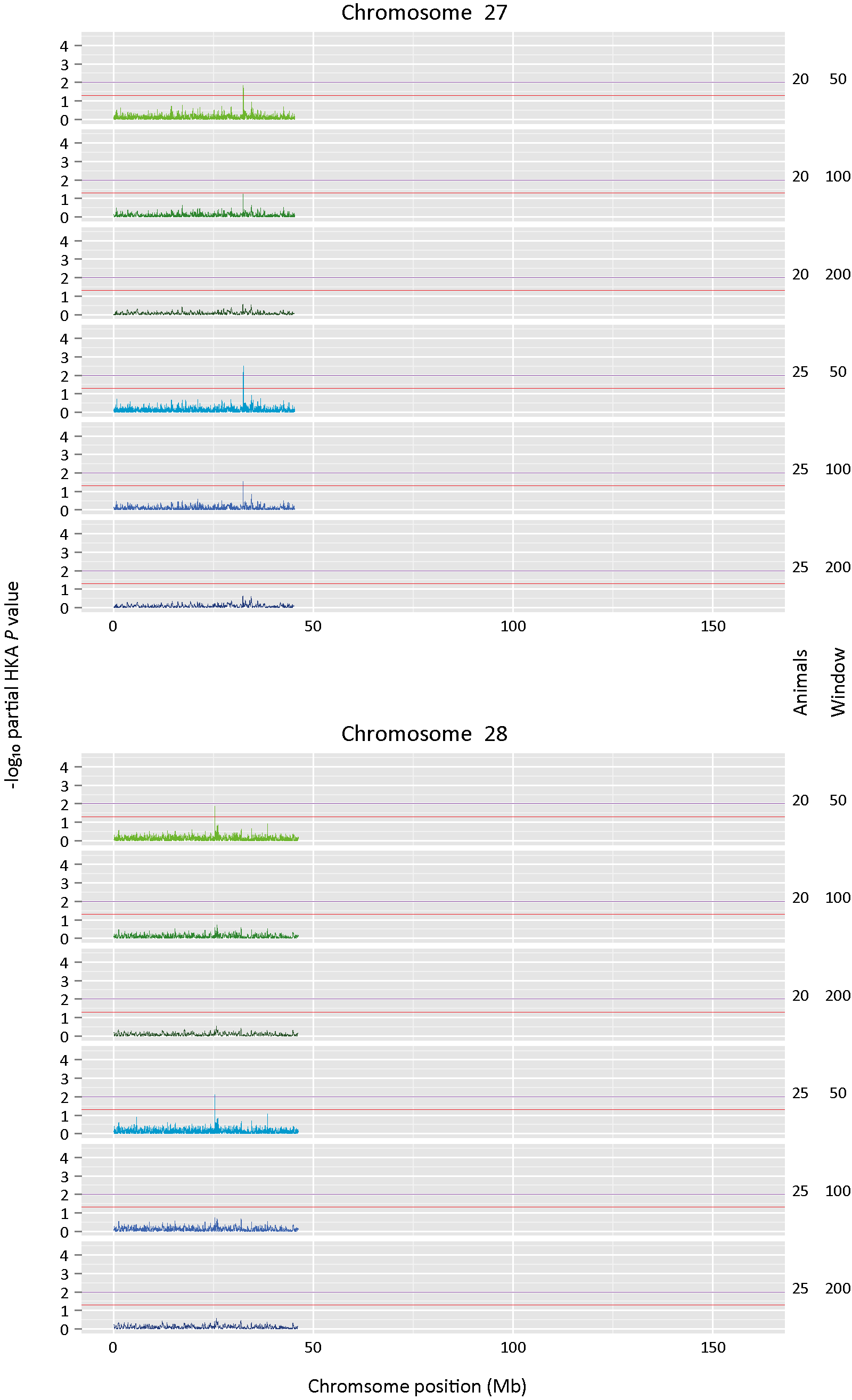

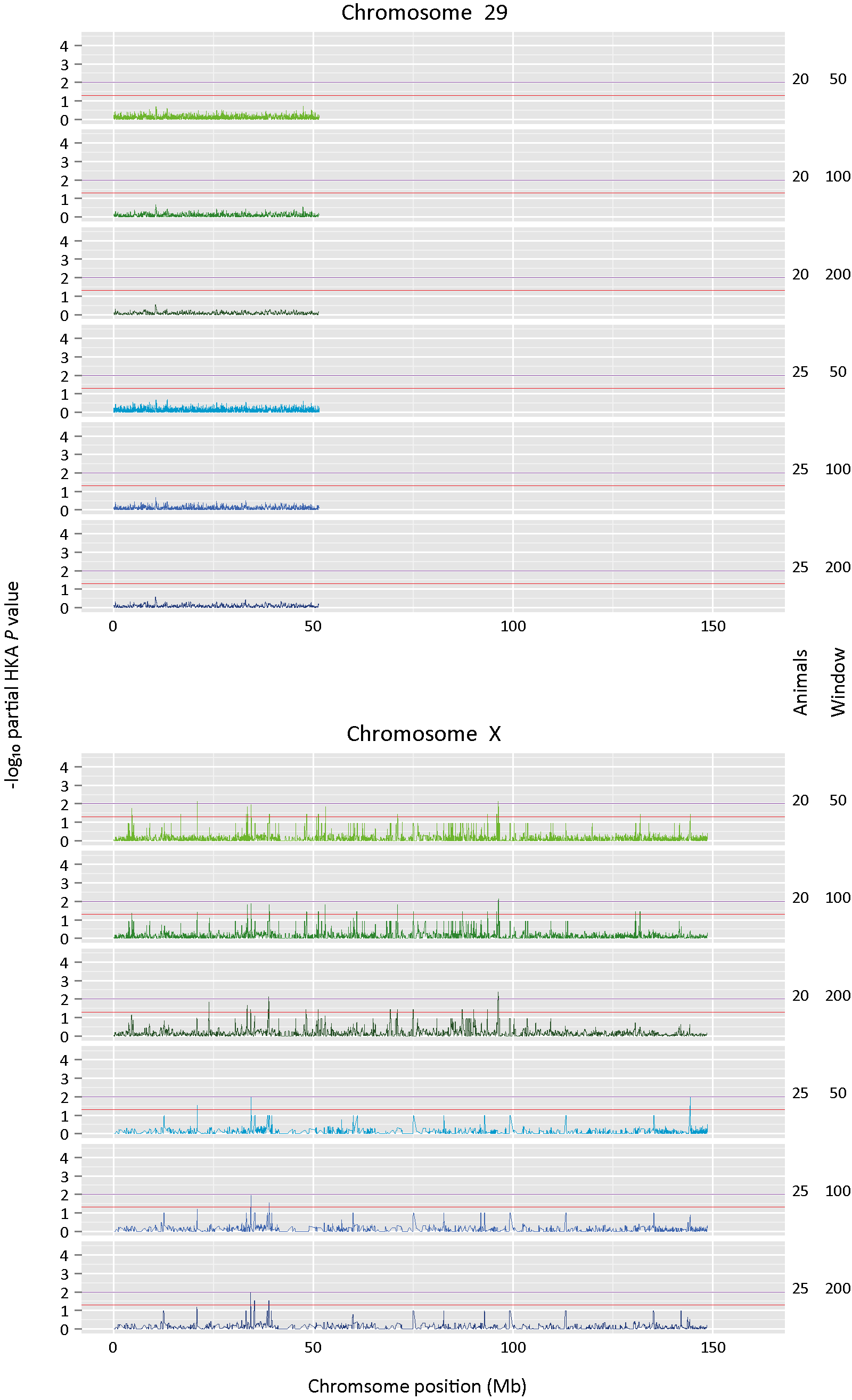


Figure S9 (pages 13–27). Detection of selective sweeps in the *B. taurus* lineage.

Bovine chromosomes showing the results of the genome-wide multilocus partial HKA tests for 50 kb, 100 kb or 200 kb overlapping windows that were moved by 10% of the window length every iteration. For the ingroup *n* = 20 or *n* = 25 resequenced European cattle were used and the CPC98 aurochs genome was used for the outgroup. Results are shown for bovine chromosomes 1–29 and the X chromosome. For each plot, the red horizontal line and the purple horizontal line indicate the uncorrected *P* ≤ 0.05 and *P* ≤ 0.01 significance thresholds, respectively. Individual chromosomal plots are shown on preceding pages.


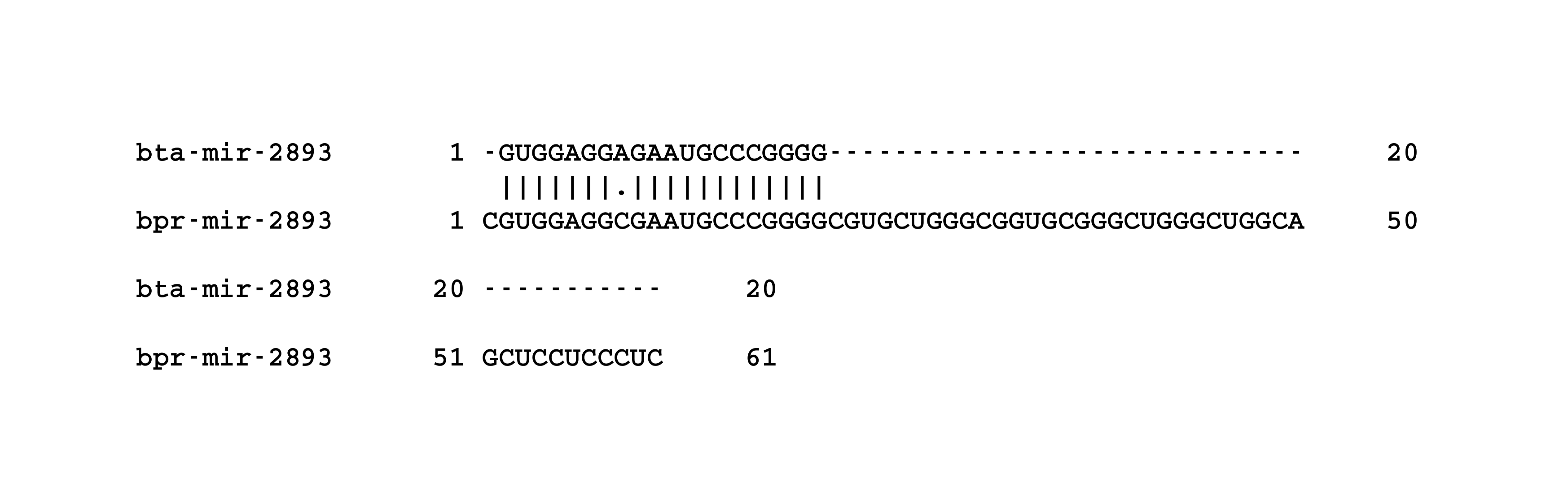


Figure S10. Alignment of bta-mir-2893 and bpr-mir-2893 sequences.

Alignment of the mature sequence of mir-2893 of *B. taurus* (bta-mir-2893) and *B. primigenius* (bpr-mir-2893) showing a transversion (A → C) in the seed region of the mature miRNA.


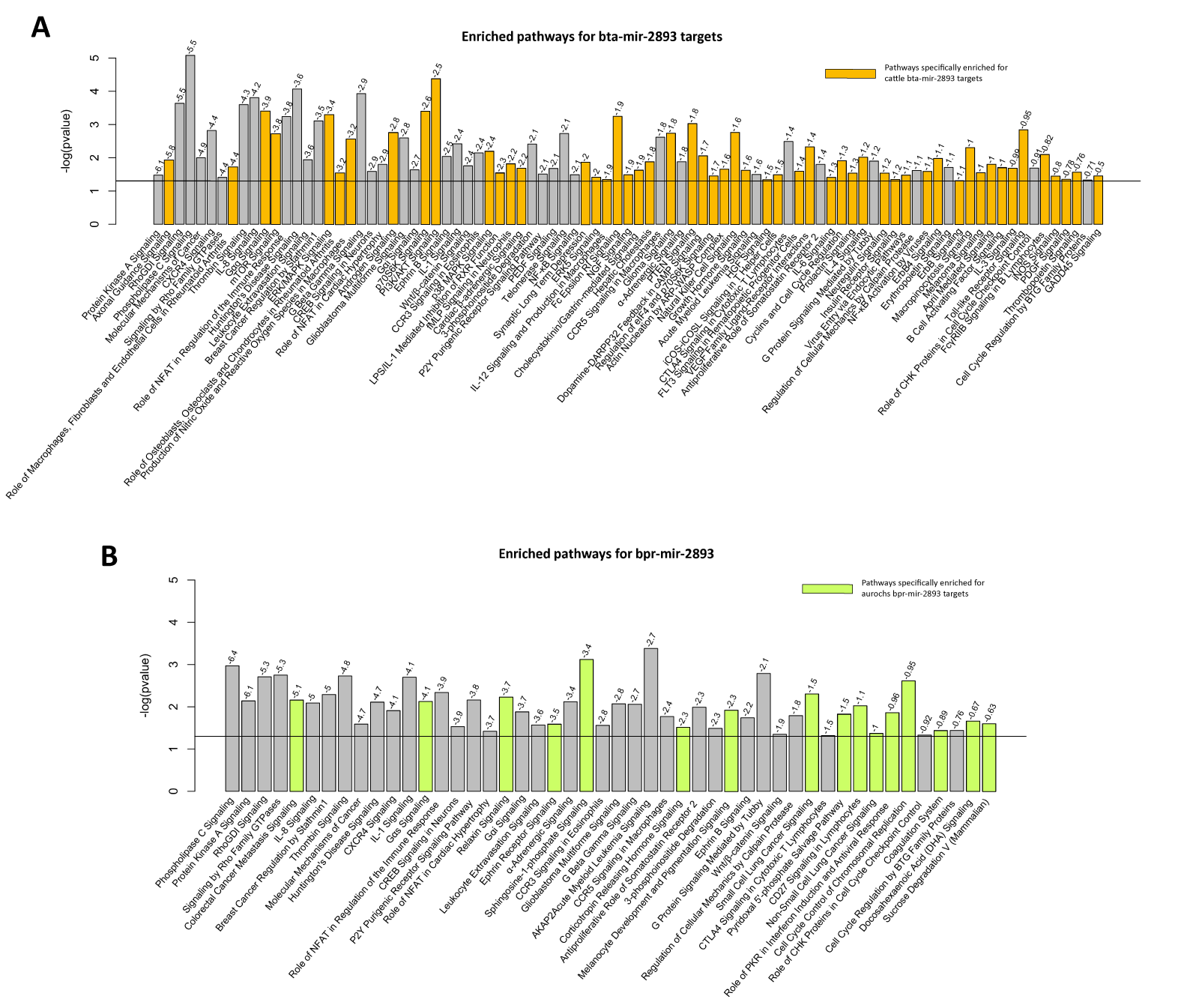


Figure S11. Pathway analysis results for bta-mir-2893 and bpr-mir-2893 targets.

Pathways significantly enriched for *B. taurus* mir-2893 (A) and *B. primigenius* (B) gene targets (*P* ≤ 0.05). Pathways are ranked by the sum of individual target *SSr* scores (shown above each bar) derived from Targetscan. The X axes show the names of the biological pathways and the Y axes shows the –log(*P*-value) of the Fisher’s exact test for enrichment of the miRNA target genes in the pathway.


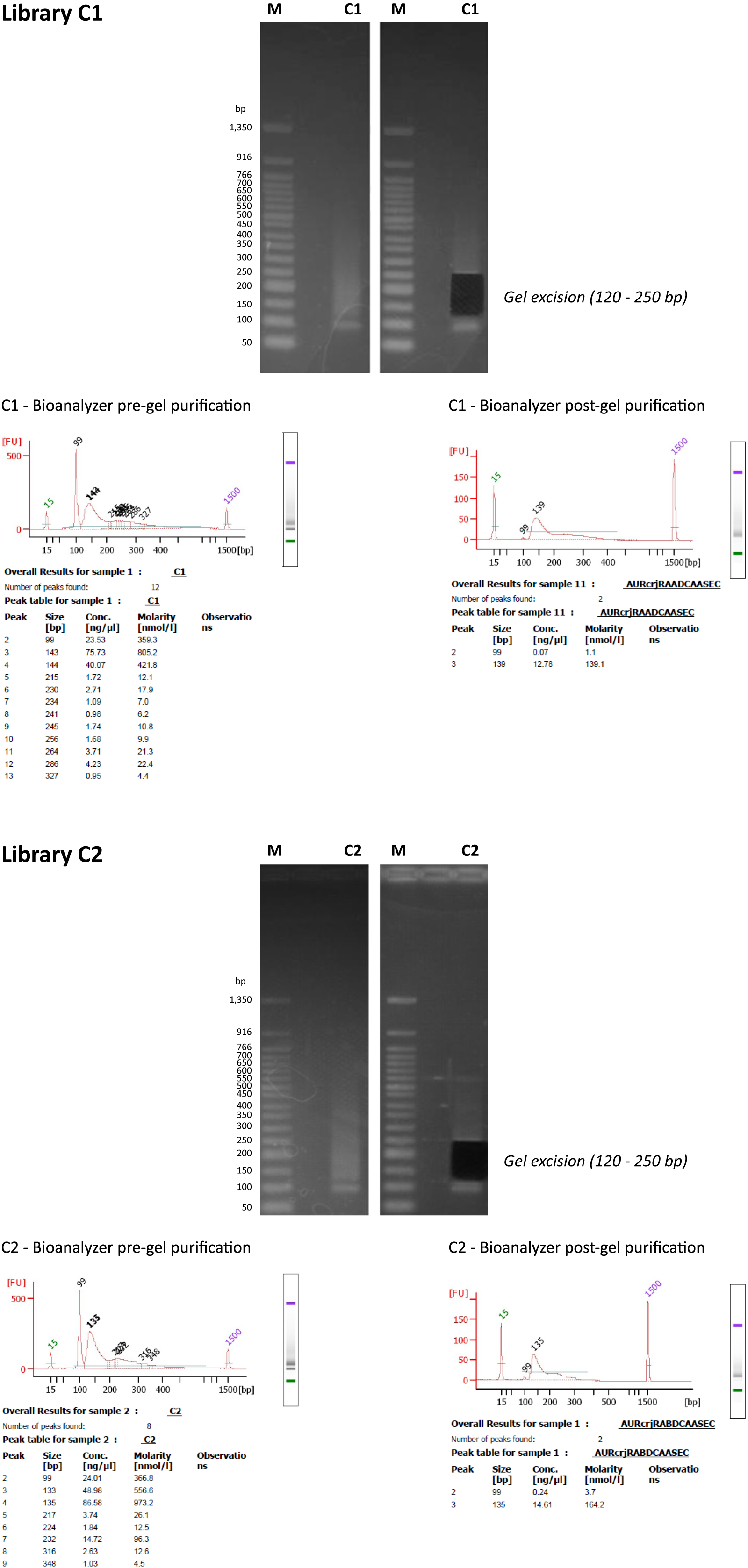


Figure S12. Agarose gel-based removal of Illumina® GA sequencing adaptor sequence from the C1 and C2 CPC98 single read libraries.

The C1 and C2 libraries were electrophoresed on 4% agarose gels and DNA fragments ranging between 120-250 bp were excised and purified. The C1 and C2 libraries were analysed pre- and post-gel purification on an Agilent Bioanalyzer using an Agilent DNA 7500 Labchip to confirm the removal/reduction of the 99 bp Illumina® GA sequencing adaptor dimers prior to sequencing. ‘M’ denotes gel lanes loaded with Quick-Load® 50 bp DNA Ladder (New England Biolabs Inc., Ipswich, MA, USA). See Supplementary Methods, Section 2 in **Additional File 1** for further details.

Supporting Tables

Table S1. Number of flow cell lanes used for six CPC98 Aurochs libraries sequenced at three different centres, using two Illumina® sequencing platforms.

|  |  | **Single-read (SR) libraries** | | | | | **Paired-end (PE) libraries** | | | | | |  |
| --- | --- | --- | --- | --- | --- | --- | --- | --- | --- | --- | --- | --- | --- |
|  |  | **C1** | **C1 (SureSelect)** | **C2** | **C2 (SureSelect)** | **C3** | **C4** | | **C5** | | **C6** | |  |
| **Centre, platform** | **Run code** | **SR run** | **SR run** | **SR run** | **SR run** | **SR run** | **SR run** | **PE-run** | **SR run** | **PE run** | **SR run** | **PE run** | **Total** |
| UCD-GA IIx | UCD-RUN-01 | 1 |  | 1 |  | 1 |  |  |  |  |  |  | 3 |
| UCD-GA IIx | UCD-RUN-02 | 3 |  | 3 |  | 1 |  |  |  |  |  |  | 7 |
| UCD-GA IIx | UCD-RUN-03 | 2 |  | 2 |  |  |  |  |  |  |  |  | 4 |
| UCD-GA IIx | UCD-RUN-04 |  |  |  |  |  | 7 |  |  |  |  |  | 7 |
| UCD-GA IIx | UCD-RUN-05 | 3 |  | 3 |  |  |  |  |  |  |  |  | 6 |
| UCD-GA IIx | UCD-RUN-06 |  | 1 |  | 1 |  |  |  |  |  |  |  | 2 |
| UCD-GA IIx | USDA-RUN-01 |  |  |  |  |  | 3 |  | 2 |  | 2 |  | 7 |
| UCD-GA IIx | USDA-RUN-02 |  |  |  |  |  |  | 14 |  |  |  |  | 14 |
| UCD-GA IIx | USDA-RUN-03 |  |  |  |  |  |  |  |  |  |  | 14 | 14 |
| UCD-GA IIx | USDA-RUN-04 |  |  |  |  |  |  |  |  | 14 |  |  | 14 |
| UCD-GA IIx | USDA-RUN-05 |  |  |  |  |  |  |  |  | 14 |  |  | 14 |
| UCD-GA IIx | USDA-RUN-06 |  |  |  |  |  |  |  | 7 |  |  |  | 7 |
| BGI-HiSeq | BGI-RUN-01 | 15 |  | 15 |  |  |  |  |  |  |  |  | 30 |
| **Total** | | 24 | 1 | 24 | 1 | 2 | 10 | 14 | 9 | 28 | 2 | 14 | **129** |

Table S2. Summary of sequencing lane data obtained for six CPC98 Aurochs libraries sequenced at three different centres, using two Illumina^®^ sequencing platforms.

| **Sequencing lane number** | **Aurochs**  **sequencing library** | **Sequencing centre** | **Sequencing**  **platform** | **Sequence read type**  **paired end: PE**  **single read: SR** | **Read length**  **(pre-trimming)** | **Mean read length**  **(post-trimming)** | **Median read length**  **(post-trimming)** |
| --- | --- | --- | --- | --- | --- | --- | --- |
| 1 | C1 | BGI | HiSeq 2000 | SR | 49 | 40.99 | 45 |
| 2 | C1 | BGI | HiSeq 2000 | SR | 49 | 40.97 | 45 |
| 3 | C1 | BGI | HiSeq 2000 | SR | 49 | 40.97 | 45 |
| 4 | C1 | BGI | HiSeq 2000 | SR | 49 | 40.99 | 45 |
| 5 | C1 | BGI | HiSeq 2000 | SR | 49 | 40.99 | 45 |
| 6 | C1 | BGI | HiSeq 2000 | SR | 49 | 40.99 | 45 |
| 7 | C1 | BGI | HiSeq 2000 | SR | 49 | 41.04 | 45 |
| 8 | C1 | BGI | HiSeq 2000 | SR | 49 | 41.02 | 45 |
| 9 | C1 | BGI | HiSeq 2000 | SR | 49 | 41.04 | 45 |
| 10 | C1 | BGI | HiSeq 2000 | SR | 49 | 41.04 | 45 |
| 11 | C1 | BGI | HiSeq 2000 | SR | 49 | 41.01 | 45 |
| 12 | C1 | BGI | HiSeq 2000 | SR | 49 | 41.03 | 45 |
| 13 | C1 | BGI | HiSeq 2000 | SR | 49 | 41.02 | 45 |
| 14 | C1 | BGI | HiSeq 2000 | SR | 49 | 40.97 | 45 |
| 15 | C1 | BGI | HiSeq 2000 | SR | 49 | 41.00 | 45 |
| 16 | C1 | UCD | GA IIx | SR | 36 | 31.91 | 33 |
| 17 | C1 | UCD | GA IIx | SR | 36 | 32.13 | 33 |
| 18 | C1 | UCD | GA IIx | SR | 36 | 32.34 | 33 |
| 19 | C1 | UCD | GA IIx | SR | 36 | 32.66 | 33 |
| 20 | C1 | UCD | GA IIx | SR | 36 | 31.92 | 33 |
| 21 | C1 | UCD | GA IIx | SR | 36 | 31.93 | 33 |
| 22 | C1 | UCD | GA IIx | SR | 42 | 36.71 | 38 |
| 23 | C1 | UCD | GA IIx | SR | 42 | 36.98 | 39 |
| 24 | C1 | UCD | GA IIx | SR | 42 | 37.06 | 39 |
| 25 | C1 (captured) | UCD | GA IIx | SR | 42 | 36.33 | 38 |
| 26 | C2 | BGI | HiSeq 2000 | SR | 49 | 38.39 | 41 |
| 27 | C2 | BGI | HiSeq 2000 | SR | 49 | 38.18 | 40 |
| 28 | C2 | BGI | HiSeq 2000 | SR | 49 | 38.18 | 40 |
| 29 | C2 | BGI | HiSeq 2000 | SR | 49 | 38.15 | 40 |
| 30 | C2 | BGI | HiSeq 2000 | SR | 49 | 38.17 | 40 |
| 31 | C2 | BGI | HiSeq 2000 | SR | 49 | 38.17 | 40 |
| 32 | C2 | BGI | HiSeq 2000 | SR | 49 | 38.16 | 40 |
| 33 | C2 | BGI | HiSeq 2000 | SR | 49 | 38.27 | 40 |
| 34 | C2 | BGI | HiSeq 2000 | SR | 49 | 38.31 | 40 |
| 35 | C2 | BGI | HiSeq 2000 | SR | 49 | 38.29 | 40 |
| 36 | C2 | BGI | HiSeq 2000 | SR | 49 | 38.28 | 40 |
| 37 | C2 | BGI | HiSeq 2000 | SR | 49 | 38.29 | 40 |
| 38 | C2 | BGI | HiSeq 2000 | SR | 49 | 38.27 | 40 |
| 39 | C2 | BGI | HiSeq 2000 | SR | 49 | 38.31 | 40 |
| 40 | C2 | BGI | HiSeq 2000 | SR | 49 | 38.20 | 40 |
| 41 | C2 | UCD | GA IIx | SR | 36 | 31.47 | 33 |
| 42 | C2 | UCD | GA IIx | SR | 36 | 32.73 | 33 |
| 43 | C2 | UCD | GA IIx | SR | 36 | 32.55 | 33 |
| 44 | C2 | UCD | GA IIx | SR | 36 | 31.90 | 33 |
| 45 | C2 | UCD | GA IIx | SR | 36 | 31.61 | 33 |
| 46 | C2 | UCD | GA IIx | SR | 36 | 31.65 | 33 |
| 47 | C2 | UCD | GA IIx | SR | 42 | 35.53 | 38 |
| 48 | C2 | UCD | GA IIx | SR | 42 | 35.71 | 38 |
| 49 | C2 | UCD | GA IIx | SR | 42 | 36.11 | 38 |
| 50 | C2 (captured) | UCD | GA IIx | SR | 42 | 35.57 | 38 |
| 51 | C3 | UCD | GA IIx | SR | 36 | 33.01 | 34 |
| 52 | C3 | UCD | GA IIx | SR | 36 | 33.18 | 34 |
| 53 | C4 | UCD | GA IIx | SR | 42 | 33.21 | 38 |
| 54 | C4 | UCD | GA IIx | SR | 42 | 31.83 | 37 |
| 55 | C4 | UCD | GA IIx | SR | 42 | 31.93 | 37 |
| 56 | C4 | UCD | GA IIx | SR | 42 | 33.77 | 39 |
| 57 | C4 | UCD | GA IIx | SR | 42 | 32.24 | 37 |
| 58 | C4 | UCD | GA IIx | SR | 42 | 33.52 | 39 |
| 59 | C4 | UCD | GA IIx | SR | 42 | 33.76 | 39 |
| 60 | C4 | USDA | GA IIx | SR | 70 | 45.33 | 44 |
| 61 | C4 | USDA | GA IIx | SR | 70 | 47.80 | 49 |
| 62 | C4 | USDA | GA IIx | SR | 70 | 47.64 | 49 |
| 63 | C4 | USDA | GA IIx | PE | 42 | 36.20 | 39 |
| 64 | C4 | USDA | GA IIx | PE | 42 | 36.36 | 40 |
| 65 | C4 | USDA | GA IIx | PE | 42 | 36.23 | 39 |
| 66 | C4 | USDA | GA IIx | PE | 42 | 36.46 | 40 |
| 67 | C4 | USDA | GA IIx | PE | 42 | 36.26 | 39 |
| 68 | C4 | USDA | GA IIx | PE | 42 | 36.57 | 40 |
| 69 | C4 | USDA | GA IIx | PE | 42 | 36.22 | 39 |
| 70 | C4 | USDA | GA IIx | PE | 42 | 36.38 | 40 |
| 71 | C4 | USDA | GA IIx | PE | 42 | 36.31 | 39 |
| 72 | C4 | USDA | GA IIx | PE | 42 | 36.49 | 40 |
| 73 | C4 | USDA | GA IIx | PE | 42 | 36.24 | 39 |
| 74 | C4 | USDA | GA IIx | PE | 42 | 36.51 | 40 |
| 75 | C4 | USDA | GA IIx | PE | 42 | 36.13 | 39 |
| 76 | C4 | USDA | GA IIx | PE | 42 | 36.28 | 39 |
| 77 | C5 | USDA | GA IIx | SR | 70 | 46.26 | 46 |
| 78 | C5 | USDA | GA IIx | SR | 70 | 48.42 | 51 |
| 79 | C5 | USDA | GA IIx | PE | 42 | 36.74 | 40 |
| 80 | C5 | USDA | GA IIx | PE | 42 | 36.78 | 40 |
| 81 | C5 | USDA | GA IIx | PE | 42 | 36.82 | 40 |
| 82 | C5 | USDA | GA IIx | PE | 42 | 36.92 | 40 |
| 83 | C5 | USDA | GA IIx | PE | 42 | 36.94 | 40 |
| 84 | C5 | USDA | GA IIx | PE | 42 | 37.00 | 40 |
| 85 | C5 | USDA | GA IIx | PE | 42 | 36.94 | 40 |
| 86 | C5 | USDA | GA IIx | PE | 42 | 37.01 | 40 |
| 87 | C5 | USDA | GA IIx | PE | 42 | 36.91 | 40 |
| 88 | C5 | USDA | GA IIx | PE | 42 | 37.06 | 40 |
| 89 | C5 | USDA | GA IIx | PE | 42 | 36.88 | 40 |
| 90 | C5 | USDA | GA IIx | PE | 42 | 37.07 | 40 |
| 91 | C5 | USDA | GA IIx | PE | 42 | 36.73 | 40 |
| 92 | C5 | USDA | GA IIx | PE | 42 | 36.81 | 40 |
| 93 | C5 | USDA | GA IIx | PE | 42 | 36.07 | 39 |
| 94 | C5 | USDA | GA IIx | PE | 42 | 38.62 | 40 |
| 95 | C5 | USDA | GA IIx | PE | 42 | 36.03 | 39 |
| 96 | C5 | USDA | GA IIx | PE | 42 | 38.36 | 40 |
| 97 | C5 | USDA | GA IIx | PE | 42 | 35.99 | 39 |
| 98 | C5 | USDA | GA IIx | PE | 42 | 37.85 | 40 |
| 99 | C5 | USDA | GA IIx | PE | 42 | 36.05 | 39 |
| 100 | C5 | USDA | GA IIx | PE | 42 | 38.17 | 40 |
| 101 | C5 | USDA | GA IIx | PE | 42 | 36.21 | 39 |
| 102 | C5 | USDA | GA IIx | PE | 42 | 38.50 | 40 |
| 103 | C5 | USDA | GA IIx | PE | 42 | 37.17 | 40 |
| 104 | C5 | USDA | GA IIx | PE | 42 | 38.41 | 40 |
| 105 | C5 | USDA | GA IIx | PE | 42 | 36.75 | 40 |
| 106 | C5 | USDA | GA IIx | PE | 42 | 39.35 | 41 |
| 107 | C5 | USDA | GA IIx | SR | 36 | 29.73 | 34 |
| 108 | C5 | USDA | GA IIx | SR | 36 | 29.83 | 34 |
| 109 | C5 | USDA | GA IIx | SR | 36 | 29.67 | 34 |
| 110 | C5 | USDA | GA IIx | SR | 36 | 29.66 | 34 |
| 111 | C5 | USDA | GA IIx | SR | 36 | 29.70 | 34 |
| 112 | C5 | USDA | GA IIx | SR | 36 | 29.74 | 34 |
| 113 | C5 | USDA | GA IIx | SR | 36 | 29.80 | 34 |
| 114 | C6 | USDA | GA IIx | SR | 70 | 50.02 | 57 |
| 115 | C6 | USDA | GA IIx | SR | 70 | 52.59 | 62 |
| 116 | C6 | USDA | GA IIx | PE | 42 | 36.48 | 39 |
| 117 | C6 | USDA | GA IIx | PE | 42 | 36.56 | 40 |
| 118 | C6 | USDA | GA IIx | PE | 42 | 36.51 | 39 |
| 119 | C6 | USDA | GA IIx | PE | 42 | 36.60 | 40 |
| 120 | C6 | USDA | GA IIx | PE | 42 | 36.52 | 39 |
| 121 | C6 | USDA | GA IIx | PE | 42 | 36.62 | 40 |
| 122 | C6 | USDA | GA IIx | PE | 42 | 36.56 | 40 |
| 123 | C6 | USDA | GA IIx | PE | 42 | 36.64 | 40 |
| 124 | C6 | USDA | GA IIx | PE | 42 | 36.58 | 40 |
| 125 | C6 | USDA | GA IIx | PE | 42 | 36.62 | 40 |
| 126 | C6 | USDA | GA IIx | PE | 42 | 36.52 | 39 |
| 127 | C6 | USDA | GA IIx | PE | 42 | 36.61 | 40 |
| 128 | C6 | USDA | GA IIx | PE | 42 | 36.55 | 40 |
| 129 | C6 | USDA | GA IIx | PE | 42 | 36.60 | 40 |

Table S3. Filtered CPC98 Aurochs sequence reads aligned to chromosomes in the *B. taurus* genome build UMD3.1 categorized by different Phred-scaled mapping quality (MAPQ) scores.

| **Chromosome** | **Chromosome length** | **All mapped reads including non-uniquely-mapped** | **Uniquely mapped**  **(MAPQ score ≥ 1)** | **Uniquely mapped**  **(MAPQ score ≥ 20)** | **Uniquely mapped**  **(MAPQ score ≥ 30)** |
| --- | --- | --- | --- | --- | --- |
| BTA01 | 158.33 Mb | 38,190,809 | 28,786,824 | 27,173,337 | 25,593,757 |
| BTA02 | 137.06 Mb | 31,601,520 | 25,130,411 | 23,920,760 | 22,560,453 |
| BTA03 | 121.43 Mb | 28,601,643 | 21,903,725 | 20,838,472 | 19,529,451 |
| BTA04 | 120.82 Mb | 29,109,160 | 22,117,997 | 21,041,927 | 19,783,647 |
| BTA05 | 121.19 Mb | 28,086,626 | 21,670,313 | 20,582,901 | 19,227,028 |
| BTA06 | 119.45 Mb | 29,424,778 | 21,413,778 | 20,269,103 | 19,054,295 |
| BTA07 | 112.63 Mb | 26,583,261 | 20,157,917 | 19,156,378 | 17,898,121 |
| BTA08 | 113.38 Mb | 28,683,951 | 20,491,927 | 19,422,303 | 18,223,050 |
| BTA09 | 105.70 Mb | 25,004,443 | 19,304,031 | 18,327,818 | 17,243,437 |
| BTA10 | 104.30 Mb | 23,854,488 | 18,968,590 | 18,125,138 | 17,033,379 |
| BTA11 | 107.31 Mb | 24,659,967 | 19,553,501 | 18,671,698 | 17,523,416 |
| BTA12 | 91.16 Mb | 20,375,122 | 15,914,592 | 15,080,323 | 14,081,001 |
| BTA13 | 84.24 Mb | 19,157,841 | 15,352,547 | 14,681,645 | 13,746,167 |
| BTA14 | 84.64 Mb | 20,122,971 | 15,455,617 | 14,707,022 | 13,793,357 |
| BTA15 | 85.29 Mb | 19,286,678 | 15,040,383 | 14,269,356 | 13,251,793 |
| BTA16 | 81.72 Mb | 18,676,014 | 14,746,041 | 14,032,195 | 13,099,480 |
| BTA17 | 75.15 Mb | 17,489,844 | 13,621,906 | 12,933,288 | 12,062,934 |
| BTA18 | 66.00 Mb | 14,679,440 | 11,718,587 | 11,214,338 | 10,372,057 |
| BTA19 | 64.05 Mb | 14,278,293 | 11,489,562 | 11,015,037 | 10,249,359 |
| BTA20 | 72.04 Mb | 16,481,521 | 13,131,840 | 12,500,953 | 11,774,414 |
| BTA21 | 71.59 Mb | 16,464,691 | 12,786,072 | 12,145,619 | 11,315,233 |
| BTA22 | 61.43 Mb | 14,194,546 | 11,362,504 | 10,916,007 | 10,276,071 |
| BTA23 | 52.53 Mb | 11,777,514 | 9,448,723 | 9,030,833 | 8,391,583 |
| BTA24 | 62.71 Mb | 14,299,827 | 11,582,962 | 11,078,083 | 10,424,140 |
| BTA25 | 42.90 Mb | 10,001,942 | 7,652,900 | 7,331,160 | 6,800,617 |
| BTA26 | 51.68 Mb | 11,743,248 | 9,384,346 | 8,961,813 | 8,411,212 |
| BTA27 | 45.40 Mb | 13,008,299 | 8,268,681 | 7,814,584 | 7,324,697 |
| BTA28 | 46.31 Mb | 10,920,014 | 8,344,172 | 7,926,999 | 7,423,973 |
| BTA29 | 51.50 Mb | 12,894,696 | 9,198,277 | 8,564,209 | 7,886,844 |
| BTAX | 148.82 Mb | 23,402,928 | 14,041,016 | 12,928,204 | 11,789,309 |
| Mitochondrion | 16,340 bp | 64,647 | 63,174 | 62,685 | 56,050 |
| Unassigned | --- | 6,683,364 | 1,811,955 | 11,22981 | 772,938 |

Table S4. CPC98 Aurochs sequence reads aligned to *Btau* UMD3.1 reference genome chromosomes.

|  |  | **All mapped reads (including reads mapped to multiple locations)** | | | | | | **Uniquely-mapped reads** | | | | |
| --- | --- | --- | --- | --- | --- | --- | --- | --- | --- | --- | --- | --- |
| **Chromosome** | **Number of bases** | **Number of reads** | **No. bases covered** | **% bases covered** | **Base pairs sequenced** | **Contigs** | **Mean read depth/**  **base** | **Reads** | **No. bases covered** | **% bases covered** | **Base pairs sequenced** | **Mean read depth/**  **base** |
| BTA01 | 158,337,067 | 38,190,809 | 156,470,470 | 98.8% | 1,363,380,590 | 1 | 8.61 | 28,786,824 | 140,920,937 | 89.0% | 1,033,195,034 | 6.53 |
| BTA02 | 137,060,424 | 31,601,520 | 135,548,547 | 98.9% | 1,119,168,897 | 1 | 8.17 | 25,130,411 | 123,193,733 | 89.9% | 896,963,649 | 6.54 |
| BTA03 | 121,430,405 | 28,601,643 | 119,951,402 | 98.8% | 1,007,693,343 | 1 | 8.30 | 21,903,725 | 108,404,429 | 89.3% | 776,472,548 | 6.39 |
| BTA04 | 120,829,699 | 29,109,160 | 119,476,093 | 98.9% | 1,032,866,685 | 1 | 8.55 | 22,117,997 | 108,100,376 | 89.5% | 790,016,368 | 6.54 |
| BTA05 | 121,191,424 | 28,086,626 | 119,558,042 | 98.7% | 990,716,306 | 1 | 8.17 | 21,670,313 | 107,794,219 | 88.9% | 768,208,602 | 6.34 |
| BTA06 | 119,458,736 | 29,424,778 | 117,923,728 | 98.7% | 1,052,587,965 | 1 | 8.81 | 21,413,778 | 104,881,524 | 87.8% | 769,604,915 | 6.44 |
| BTA07 | 112,638,659 | 26,583,261 | 110,172,685 | 97.8% | 936,103,374 | 1 | 8.31 | 20,157,917 | 99,727,629 | 88.5% | 713,298,191 | 6.33 |
| BTA08 | 113,384,836 | 28,683,951 | 112,023,349 | 98.8% | 1,018,212,351 | 1 | 8.98 | 20,491,927 | 100,601,929 | 88.7% | 730,197,360 | 6.44 |
| BTA09 | 105,708,250 | 25,004,443 | 104,474,304 | 98.8% | 890,417,408 | 1 | 8.42 | 19,304,031 | 94,163,028 | 89.1% | 692,080,408 | 6.55 |
| BTA10 | 104,305,016 | 23,854,488 | 103,016,365 | 98.8% | 841,631,614 | 1 | 8.07 | 18,968,590 | 93,089,139 | 89.2% | 673,352,097 | 6.46 |
| BTA11 | 107,310,763 | 24,659,967 | 105,899,918 | 98.7% | 860,156,513 | 1 | 8.02 | 19,553,501 | 97,066,568 | 90.5% | 686,009,161 | 6.39 |
| BTA12 | 91,163,125 | 20,375,122 | 89,086,470 | 97.7% | 722,995,322 | 1 | 7.93 | 15,914,592 | 79,148,531 | 86.8% | 569,136,495 | 6.24 |
| BTA13 | 84,240,350 | 19,157,841 | 83,198,228 | 98.8% | 664,872,323 | 1 | 7.89 | 15,352,547 | 76,410,950 | 90.7% | 535,683,653 | 6.36 |
| BTA14 | 84,648,390 | 20,122,971 | 83,564,508 | 98.7% | 711,175,747 | 1 | 8.40 | 15,455,617 | 75,173,078 | 88.8% | 549,027,574 | 6.49 |
| BTA15 | 85,296,676 | 19,286,678 | 83,941,092 | 98.4% | 679,762,562 | 1 | 7.97 | 15,040,383 | 75,370,742 | 88.4% | 533,602,823 | 6.26 |
| BTA16 | 81,724,687 | 18,676,014 | 80,573,415 | 98.6% | 653,188,435 | 1 | 7.99 | 14,746,041 | 73,423,781 | 89.8% | 519,458,928 | 6.36 |
| BTA17 | 75,158,596 | 17,489,844 | 74,112,976 | 98.6% | 612,427,078 | 1 | 8.15 | 13,621,906 | 67,235,245 | 89.5% | 479,583,666 | 6.38 |
| BTA18 | 66,004,023 | 14,679,440 | 64,847,450 | 98.2% | 502,102,902 | 1 | 7.61 | 11,718,587 | 59,158,640 | 89.6% | 403,007,780 | 6.11 |
| BTA19 | 64,057,457 | 14,278,293 | 63,075,424 | 98.5% | 485,584,811 | 1 | 7.58 | 11,489,562 | 58,440,604 | 91.2% | 392,995,027 | 6.14 |
| BTA20 | 72,042,655 | 16,481,521 | 71,164,164 | 98.8% | 582,933,082 | 1 | 8.09 | 13,131,840 | 64,722,261 | 89.8% | 468,176,824 | 6.50 |
| BTA21 | 71,599,096 | 16,464,691 | 70,594,007 | 98.6% | 574,135,313 | 1 | 8.02 | 12,786,072 | 64,221,615 | 89.7% | 448,712,770 | 6.27 |
| BTA22 | 61,435,874 | 14,194,546 | 60,714,702 | 98.8% | 492,858,539 | 1 | 8.02 | 11,362,504 | 56,079,343 | 91.3% | 396,393,782 | 6.45 |
| BTA23 | 52,530,062 | 11,777,514 | 51,716,907 | 98.5% | 407,259,144 | 1 | 7.75 | 9,448,723 | 47,092,471 | 89.6% | 329,589,031 | 6.27 |
| BTA24 | 62,714,930 | 14,299,827 | 62,030,456 | 98.9% | 502,480,562 | 1 | 8.01 | 11,582,962 | 56,804,881 | 90.6% | 409,782,844 | 6.53 |
| BTA25 | 42,904,170 | 10,001,942 | 42,268,790 | 98.5% | 337,162,681 | 1 | 7.86 | 7,652,900 | 39,081,785 | 91.1% | 259,221,137 | 6.04 |
| BTA26 | 51,681,464 | 11,743,248 | 51,009,161 | 98.7% | 409,844,191 | 1 | 7.93 | 9,384,346 | 46,521,850 | 90.0% | 329,340,008 | 6.37 |
| BTA27 | 45,407,902 | 13,008,299 | 44,707,000 | 98.5% | 462,605,739 | 1 | 10.19 | 8,268,681 | 40,265,169 | 88.7% | 292,684,783 | 6.45 |
| BTA28 | 46,312,546 | 10,920,014 | 45,737,771 | 98.8% | 384,202,700 | 1 | 8.30 | 8,344,172 | 41,532,898 | 89.7% | 294,875,956 | 6.37 |
| BTA29 | 51,505,224 | 12,894,696 | 50,638,413 | 98.3% | 448,669,942 | 1 | 8.71 | 9,198,277 | 45,913,003 | 89.1% | 319,973,454 | 6.21 |
| BTAX | 148,823,899 | 23,402,928 | 142,864,426 | 96.0% | 829,236,211 | 1 | 5.57 | 14,041,016 | 118,220,827 | 79.4% | 501,187,184 | 3.37 |
| Mitochondrion | 16,340 | 64,647 | 16,340 | 100.0% | 2,637,455 | 1 | 161.41 | 63,174 | 16,340 | 100.0% | 2,582,767 | 158.06 |
| Unassigned | 9,499,556 | 6,539,006 | 8,936,691 | 94.1% | 234,758,994 | 3,286 | 24.71 | 1,811,955 | 4,706,826 | 49.5% | 59,640,213 | 6.28 |
| **Whole genome** | **2,670,422,301** | **619,659,728** | **2,629,313,294** | **98.5%** | **21,813,828,779** | **3,317** | **8.17** | **469,914,871** | **2,367,484,351** | **88.7%** | **16,624,055,032** | **6.23** |

|  | **Allele counts for 236 haplogroup P, Q, R, T and I complete mtDNA genome sequences from GenBank** | | | | | | | | | | | | | | | | | | | |  |  |  |  |
| --- | --- | --- | --- | --- | --- | --- | --- | --- | --- | --- | --- | --- | --- | --- | --- | --- | --- | --- | --- | --- | --- | --- | --- | --- |
|  | **A** | | | | | **C** | | | | | **G** | | | | | **T** | | | | | **Aurochs CPC98 sequence reads** | | | |
| **Bovine mtDNA reference position** | **P** | **Q** | **R** | **T** | **I** | **P** | **Q** | **R** | **T** | **I** | **P** | **Q** | **R** | **T** | **I** | **P** | **Q** | **R** | **T** | **I** | **Total no. reads** | **P allele reads** | **T, Q, R or I allele reads** | **Other allele reads** |
| **301** | --- | --- | --- | --- | --- | --- | 16 | 10 | 199 | 8 | --- | --- | --- | --- | --- | **3** | --- | --- | --- | --- | 120 | 120 | --- | --- |
| **1,128** | --- | 16 | 10 | 199 | 8 | --- | --- | --- | --- | --- | **3** | --- | --- | --- | --- | --- | --- | --- | --- | --- | 152 | 148 | 4 | --- |
| **2,585** | --- | --- | --- | --- | --- | **3** | --- | --- | --- | --- | --- | --- | --- | --- | --- | --- | 16 | 10 | 199 | 8 | 120 | 120 | --- | --- |
| **4,676** | --- | 16 | 10 | 199 | 8 | --- | --- | --- | --- | --- | **3** | --- | --- | --- | --- | --- | --- | --- | --- | --- | 136 | 136 | --- | --- |
| **5,899** | --- | 16 | 10 | 199 | 8 | --- | --- | --- | --- | --- | **3** | --- | --- | --- | --- | --- | --- | --- | --- | --- | 137 | 136 | 1 | --- |
| **7,952** | --- | --- | --- | --- | --- |  | 16 | 10 | 199 | 8 | --- | --- | --- | --- | --- | **3** | --- | --- | --- | --- | 149 | 148 | 1 | --- |
| **8,236** | --- | --- | --- | --- | --- | **3** | --- | --- | --- | --- | --- | --- | --- | --- | --- | --- | 16 | 10 | 199 | 8 | 116 | 114 | 2 | --- |
| **8,358** | --- | --- | --- | --- | --- | --- | 16 | 10 | 199 | 8 | --- | --- | --- | --- | --- | **3** | --- | --- | --- | --- | 118 | 118 | --- | --- |
| **10,126** | --- | --- | --- | --- | --- | --- | 16 | 10 | 199 | 8 | --- | --- | --- | --- | --- | **3** | --- | --- | --- | --- | 125 | 125 | --- | --- |
| **11,140** | --- | 16 | 10 | 199 | 8 | --- | --- | --- | --- | --- | **3** | --- | --- | --- | --- | --- | --- | --- | --- | --- | 145 | 145 | --- | --- |
| **12,016** | --- | --- | --- | --- | --- | **3** | --- | --- | --- | --- | --- | --- | --- | --- | --- | --- | 16 | 10 | 199 | 8 | 147 | 147 | --- | --- |
| **13,821** | --- | 16 | 10 | 199 | 8 | --- | --- | --- | --- | --- | **3** | --- | --- | --- | --- | --- | --- | --- | --- | --- | 152 | 152 | --- | --- |
| **14,129** | --- | --- | --- | --- | 8 | --- | --- | --- | --- | --- | --- | 16 | 10 | 199 | --- | **3** | --- | --- | --- | --- | 97 | 97 | --- | --- |
| **14,873** | **3** | --- | --- | --- | --- | --- | --- | --- | --- | --- | --- | 16 | 10 | 199 | 8 | --- | --- | --- | --- | --- | 124 | 124 | --- | --- |
| **15,673** | --- | --- | --- | --- | --- | **3** | --- | --- | --- | --- | --- | --- | --- | --- | --- | --- | 16 | 10 | 199 | 8 | 121 | 118 | 2 | 1 |
| **Total** | **3** | 80 | 50 | 995 | 48 | **12** | 64 | 40 | 796 | 32 | **15** | 32 | 20 | 398 | 8 | **15** | 64 | 40 | 796 | 32 | **1,959** | **1,948** | **10** | **1** |

Table S5. Estimating contamination from modern cattle mtDNA using mtDNA haplotype information and CPC98 Aurochs sequence reads.

Aggregate estimate of modern cattle mtDNA contamination: $\frac{10 modern mtDNA reads}{1,959 modern mtDNA reads}=0.51\%$

Table S6. Cattle breed and population information for 1,228 animals with Illumina^®^ BovineSNP50 SNP genotype data used for phylogenetic and population genomics analyses.

| **Population**  **ID** | **Breed/**  **population** | **Country of origin** | **Country sampled** | **Breed group** | **Estimated latitude^1^** | **Estimated**  **Longitude^1^** | **Sample size (n)** | **Observed Hz ± SD** | **Included in phylogenetic tree** | **Sample source**  **citation(s)** |
| --- | --- | --- | --- | --- | --- | --- | --- | --- | --- | --- |
| ABND | Abondance | France | France | Continental Europe taurine | 46° 12' N | 6° 46' E | 22 | 0.307 ± 0.005 | Y | [2] |
| ANGU | Angus | Scotland | New Zealand, United States | Britain and Ireland taurine | 57° 0' N | 2° 50' W | 40 | 0.296 ± 0.019 | Y | [3, 4] |
| AUBR | Aubrac | France | France | Continental Europe taurine | 44° 34' N | 3° 5' E | 22 | 0.300 ± 0.011 | Y | [2] |
| AURO | Aurochs | England | United Kingdom | Aurochs | --- | --- | 1 | 0.25 | Y | Current study |
| BAOU | Baoule | Burkina Faso | Burkina Faso | West Africa taurine | --- | --- | 24 | 0.217 ± 0.010 | Y | [5] |
| BBLU | Belgian Blue | Belgium | United States | Continental Europe taurine | 50° 30' N | 5° 0' E | 4 | 0.323 ± 0.004 | Y | [4] |
| BDAQ | Blonde d'Aquitaine | France | United States | Continental Europe taurine | 43° 46' N | 0° 18' E | 5 | 0.308 ± 0.009 | Y | [4] |
| BFMS | Beefmaster | United States | United States | North America taurine-zebu hybrid | --- | --- | 21 | 0.326 ± 0.011 | N | [3] |
| BGAL | Belted Galloway | Scotland | Scotland | Britain and Ireland taurine | 55° 6' N | 4° 0' W | 4 | 0.283 ± 0.009 | Y | [4] |
| BGRN | *Bos grunniens* (Yak) | Central Asia/  Himalayas | United States | Outgroup species | --- | --- | 2 | 0.009 ± 0.001 | N | [4] |
| BORG | Borgou | Benin | Benin | West Africa taurine | --- | --- | 27 | 0.266 ± 0.014 | N | [5] |
| BRBP | Bretonne Black Pied | France | France | Continental Europe taurine | 48° 12' N | 3° 14' 6W | 17 | 0.315 ± 0.019 | Y | [2] |
| BRSW | Brown Swiss | Switzerland | France, United States | Continental Europe taurine | 47° 5' N | 8° 41' 6E | 29 | 0.289 ± 0.015 | Y | [2-4] |
| BSHN | British Shorthorn | England | United States | Britain and Ireland taurine | 54° 38' N | 1° 45' W | 10 | 0.246 ± 0.012 | Y | [4] |
| CHAR | Charolais | France | France, United Kingdom, United States | Continental Europe taurine | 46° 26' N | 4° 17' E | 40 | 0.318 ± 0.008 | Y | [2-4] |
| CHNN | Chianina | Italy | United States | Continental Europe taurine | 43° 14' N | 11° 49' 6E | 7 | 0.304 ± 0.015 | Y | [4] |
| CORR | Corriente | United States/Mexico | United States | North America taurine | --- | --- | 4 | 0.310 ± 0.019 | Y | [4] |
| DEVN | Devon | England | United Kingdom | Britain and Ireland taurine | 51° 0' N | 3° 42' W | 4 | 0.277 ± 0.013 | Y | [4] |
| DXTR | Dexter | Ireland | United Kingdom | Britain and Ireland taurine | 52° 10' N | 8° 40' W | 4 | 0.236 ± 0.044 | Y | [4] |
| ELHN | English Longhorn | England | United Kingdom | Britain and Ireland taurine | 53° 57' 3N | 2° 0' 6W | 3 | 0.217 ± 0.012 | Y | [4] |
| FAYR | Finnish Ayrshire | Scotland | Finland | Britain and Ireland taurine | 55° 30' N | 4° 22' W | 8 | 0.303 ± 0.005 | Y | [4] |
| FRPL | French Red Pied Lowland | France | France | Continental Europe taurine | --- | --- | 22 | 0.324 ± 0.010 | Y | [2] |
| GALL | Galloway | Scotland | United Kingdom | Britain and Ireland taurine | 55° 6' N | 4° 0' W | 4 | 0.286 ± 0.012 | Y | [4] |
| GASC | Gascon | France | France | Continental Europe taurine | 43° 15' N | 0° 20' E | 21 | 0.303 ± 0.007 | Y | [2] |
| GELB | Gelbvieh | Germany | United States | Continental Europe taurine | 50° 0' N | 11° 30' E | 8 | 0.308 ± 0.008 | Y | [4, 6] |
| GNSY | Guernsey | Channel Islands | United Kingdom, United States | Britain and Ireland taurine | 49° 27' N | 2° 34' 4W | 19 | 0.274 ± 0.014 | Y | [3, 4] |
| HANW | Hanwoo | Korea | South Korea | East Asia taurine | --- | --- | 6 | 0.293 ± 0.005 | Y | [4] |
| HOLS | Holstein | The Netherlands | France, New Zealand, United States | Continental Europe taurine | 53° 20' N | 7° 17' 3E | 40 | 0.314 ± 0.012 | Y | [3, 4, 7] |
| HRFD | Hereford | England | New Zealand, United States | Britain and Ireland taurine | 52° 6' N | 2° 44' W | 35 | 0.300 ± 0.036 | Y | [3, 4] |
| JRSY | Jersey | Channel Islands | France, New Zealand, United States | Britain and Ireland taurine | 49° 12' N | 2° 7' W | 40 | 0.269 ± 0.016 | Y | [2-4] |
| KRRY | Kerry | Ireland | Ireland, United Kingdom | Britain and Ireland taurine | 52° 4' N | 9° 20' W | 32 | 0.300 ± 0.013 | Y | [Current study, 4] |
| KURI | Kuri | Chad | Chad | West Africa taurine | --- | --- | 28 | 0.262 ± 0.007 | N | [5] |
| LAGN | Lagune | Benin | Benin | West Africa taurine | --- | --- | 27 | 0.185 ± 0.013 | Y | [5] |
| LIMS | Limousin | France | France, United States | Continental Europe taurine | 45° 47' 6N | 1° 38' E | 40 | 0.309 ± 0.010 | Y | [3, 4] |
| LNCR | Lincoln Red | England | United States | Britain and Ireland taurine | 53° 9' N | 0° 12' W | 7 | 0.268 ± 0.020 | Y | [4] |
| MANJ | Maine-Anjou | France | France, United States | Continental Europe taurine | (47° 42' N) | (0° 40' W | 20 | 0.302 ± 0.006 | Y | [2, 4] |
| MARA | Maraichine | France | France | Continental Europe taurine | 46° 30' N | 1° 11' 6W | 18 | 0.317 ± 0.011 | Y | [2] |
| MARC | Marchigiana | Italy | United States | Continental Europe taurine | 43° 29' N | 13° 1' E | 3 | 0.313 ± 0.013 | Y | [4] |
| MONT | Montbeliarde | France | France, United States | Continental Europe taurine | 47° 31' N | 6° 48' E | 32 | 0.297 ± 0.010 | Y | [4, 7] |
| MUGR | Murray Grey | Australia | United States | Britain and Ireland taurine | --- | --- | 4 | 0.304 ± 0.007 | Y | [4] |
| NDAB | N'Dama | Burkina Faso | Burkina Faso | West Africa taurine | --- | --- | 19 | 0.232 ± 0.010 | N | [5] |
| NDAG | N'Dama | Guinea | Guinea | West Africa taurine | --- | --- | 20 | 0.211 ± 0.008 | Y | [3] |
| NDAM | N'Dama | Gambia | Gambia | West Africa taurine | --- | --- | 5 | 0.245 ± 0.006 | N | [4] |
| NORM | Normande | France | France | Continental Europe taurine | 48° 54' N | 0° 18' E | 30 | 0.304 ± 0.006 | Y | [7] |
| NRED | Norwegian Red | Norway | Norway | Continental Europe taurine | 60° 15' N | 10° 3' E | 20 | 0.313 ± 0.009 | Y | [3] |
| OLMZ | Oulmès Zaer | Morocco | Morocco | North Africa taurine | --- | --- | 24 | 0.289 ± 0.012 | N | [5] |
| PDMT | Piedmontese | Italy | Italy, United States | Continental Europe taurine | 45° 2' 6N | 7° 31' 6E | 23 | 0.321 ± 0.005 | Y | [3, 4] |
| PINZ | Pinzgauer | Austria | United States | Continental Europe taurine | 47° 19' N | 12° 48' E | 5 | 0.310 ± 0.006 | Y | [4] |
| RANG | Red Angus | Scotland | United States, Canada | Britain and Ireland taurine | 57° 0' N | 2° 50' W | 14 | 0.303 ± 0.010 | Y | [3] |
| REDP | Red Poll | England | United States | Britain and Ireland taurine | 52° 28' N | 1° 2' E | 5 | 0.280 ± 0.009 | Y | [4] |
| ROMG | Romagnola | Italy | Italy | Continental Europe taurine | 44° 32' N | 11° 3' E | 21 | 0.292 ± 0.009 | Y | [3] |
| ROMS | Romosinuano | Colombia | United States | South America taurine | --- | --- | 8 | 0.294 ± 0.015 | Y | [4] |
| SALR | Salers | France | France, United States | Continental Europe taurine | 45° 6' N | 2° 42' E | 27 | 0.284 ± 0.021 | Y | [2, 4] |
| SCHL | Scottish Highland | Scotland | United Kingdom, United States | Britain and Ireland taurine | 57° 8' N | 4° 45' W | 8 | 0.273 ± 0.009 | Y | [4] |
| SDEV | South Devon | England | United Kingdom | Britain and Ireland taurine | 50° 22' N | 3° 45' W | 3 | 0.297 ± 0.005 | Y | [4] |
| SGRT | Santa Gertrudis | United States | United States | North America taurine-zebu hybrid | --- | --- | 19 | 0.315 ± 0.008 | N | [3] |
| SIMM | Simmental | Switzerland | United States | Continental Europe taurine | 46° 56' 3N | 7° 26' E | 10 | 0.309 ± 0.013 | Y | [4, 6] |
| SOMB | Somba | Togo | Togo | West Africa taurine | --- | --- | 30 | 0.220 ± 0.017 | Y | [5] |
| SUSX | Sussex | England | United Kingdom | Britain and Ireland taurine | 51° 6' N | 0° 7' E | 4 | 0.271 ± 0.017 | Y | [4] |
| TARE | Tarentaise/Tarine | France | France | Continental Europe taurine | 45° 21' 3N | 4° 30' E | 18 | 0.299 ± 0.011 | Y | [2] |
| TXLH | Texas Longhorn | United States | United States | North America taurine | --- | --- | 10 | 0.314 ± 0.019 | Y | [4] |
| VOSG | Vosgienne | France | France | Continental Europe taurine | 48° 15' N | 7° 16' E | 19 | 0.309 ± 0.010 | Y | [2] |
| WAGY | Wagyu | Japan | United States | East Asia taurine | --- | --- | 6 | 0.251 ± 0.028 | **Y** | [4] |
| WBLK | Welsh Black | Wales | United Kingdom | Britain and Ireland taurine | 52° 20' N | 3° 30' W | 2 | 0.308 ± 0.008 | **Y** | [4] |
| WHPK | White Park | England | United Kingdom | Britain and Ireland taurine | 55° 30' 3N | 1° 53' W | 4 | 0.219 ± 0.020 | Y | [4] |
| ZBOR | Zebu Bororo | West Africa Various | Chad | West Africa taurine-zebu hybrid | --- | --- | 23 | 0.243 ± 0.009 | N | [5] |
| ZBRA | Brahman | United States | Australia, United States | North America zebu | --- | --- | 22 | 0.190 ± 0.015 | Y | [3] |
| ZFUL | Zebu Fulani | West Africa Various | Benin | West Africa taurine-zebu hybrid | --- | --- | 23 | 0.245 ± 0.011 | N | [5] |
| ZGIR | Gir | India | Brazil | South Asia zebu | --- | --- | 22 | 0.161 ± 0.007 | Y | [3, 4] |
| ZGUZ | Guzerat | Brazil | Brazil | South America zebu | --- | --- | 3 | 0.164 ± 0.009 | N | [4] |
| ZMAD | Madagascar Zebu | Madagascar | Madagascar | East Africa zebu | --- | --- | 30 | 0.196 ± 0.010 | N | [5] |
| ZNEL | Nelore | Brazil | Brazil | South America zebu | --- | --- | 21 | 0.164 ± 0.008 | Y | [3, 4] |
| ZSAH | Sahiwal | Pakistan | Pakistan | South Asia zebu | --- | --- | 10 | 0.162 ± 0.009 | N | [4] |
| ZSHK | Sheko | Ethiopia | Ethiopia | East Africa taurine-zebu hybrid | --- | --- | 16 | 0.253 ± 0.004 | N | [3] |
| Totals | 74 breeds/populations including the aurochs | | --- | --- | --- | --- | 1,228 | 0.275 ± 0.048 | --- | --- |

^1^ Estimated latitude and longitude used for geographical coordinates on **Figure 5**.

Table S7. Mean ABBA/BABA test *D* statistics of admixture with British aurochs (recent outgroup O1) for individual European cattle breeds (P1) estimated using seven West African taurine populations (P2).

Populations are ranked by *D* statistic and the heat map colour range corresponds to these values.

| **Population**  **ID** | **Breed name** | **Country of origin** | **Estimated**  **Latitude^1^** | **Estimate**  **Longitude^1^** | **Mean *D* estimated from West African**  **taurine populations** |
| --- | --- | --- | --- | --- | --- |
| SCHL | Scottish Highland | Scotland | 57° 8' N | 4° 45' W | 0.1604 |
| DXTR | Dexter | Ireland | 52° 10' N | 8° 40' W | 0.1560 |
| KRRY | Kerry | Ireland | 52° 4' N | 9° 20' W | 0.1543 |
| WBLK | Welsh Black | Wales | 52° 20' N | 3° 30' W | 0.1502 |
| WHPK | White Park | England | 55° 30' 30" N | 1° 53' W | 0.1500 |
| ANGU | Angus | Scotland | 57° 0' N | 2° 50' W | 0.1475 |
| GNSY | Guernsey | Channel Islands | 49° 27' N | 2° 34' 40" W | 0.1474 |
| FAYR | Finnish Ayrshire | Scotland | 55° 30' N | 4° 22' W | 0.1464 |
| ELHN | English Longhorn | England | 53° 57' 30" N | 2° 1' W | 0.1460 |
| DEVN | Devon | England | 51° 0' N | 3° 42' W | 0.1453 |
| HRFD | Hereford | England | 52° 6' N | 2° 44' W | 0.1449 |
| LNCR | Lincoln Red | England | 53° 9' N | 0° 12' W | 0.1433 |
| RANG | Red Angus | Scotland | 57° 0' N | 2° 50' W | 0.1417 |
| BGAL | Belted Galloway | Scotland | 55° 6' N | 4° 0' W | 0.1406 |
| NORM | Normande | France | 48° 54' N | 0° 18' E | 0.1394 |
| SUSX | Sussex | England | 51° 6' N | 0° 7' E | 0.1388 |
| BSHN | British Shorthorn | England | 54° 38' N | 1° 45' W | 0.1387 |
| REDP | Red Poll | England | 52° 28' N | 1° 2' E | 0.1384 |
| MARA | Maraichine | France | 46° 30' N | 1° 12' W | 0.1381 |
| NRED | Norwegian Red | Norway | 60° 15' N | 10° 3' E | 0.1379 |
| SDEV | South Devon | England | 50° 22' N | 3° 45' W | 0.1374 |
| BBLU | Belgian Blue | Belgium | 50° 30' N | 5° 0' E | 0.1370 |
| LIMS | Limousin | France | 45° 48' N | 1° 38' E | 0.1354 |
| BRBP | Bretonne Black Pied | France | 48° 12' N | 3° 15' W | 0.1353 |
| SIMM | Simmental | Switzerland | 46° 56' 30" N | 7° 26' E | 0.1350 |
| GALL | Galloway | Scotland | 55° 6' N | 4° 0' W | 0.1333 |
| SALR | Salers | France | 45° 6' N | 2° 42' E | 0.1333 |
| JRSY | Jersey | Channel Islands | 49° 12' N | 2° 7' W | 0.1318 |
| TARE | Tarentaise/Tarine | France | 45° 21' 30" N | 4° 30' E | 0.1306 |
| HOLS | Holstein | The Netherlands | 53° 20' N | 7° 17' 30" E | 0.1285 |
| ABND | Abondance | France | 46° 12' N | 6° 46' E | 0.1280 |
| BDAQ | Blonde d'Aquitaine | France | 43° 46' N | 0° 18' E | 0.1278 |
| AUBR | Aubrac | France | 44° 34' N | 3° 5' E | 0.1275 |
| GELB | Gelbvieh | Germany | 50° 0' N | 11° 30' E | 0.1273 |
| MONT | Montbeliarde | France | 47° 31' N | 6° 48' E | 0.1268 |
| PDMT | Piedmontese | Italy | 45° 3' N | 7° 32' E | 0.1259 |
| VOSG | Vosgienne | France | 48° 15' N | 7° 16' E | 0.1257 |
| BRSW | Brown Swiss | Switzerland | 47° 5' N | 8° 42' E | 0.1248 |
| GASC | Gascon | France | 43° 15' N | 0° 20' E | 0.1214 |
| PINZ | Pinzgauer | Austria | 47° 19' N | 12° 48' E | 0.1207 |
| CHAR | Charolais | France | 46° 26' N | 4° 17' E | 0.1171 |
| CHNN | Chianina | Italy | 43° 14' N | 11° 50' E | 0.0780 |
| ROMG | Romagnola | Italy | 44° 32' N | 11° 3' E | 0.0711 |
| MARC | Marchigiana | Italy | 43° 29' N | 13° 1' E | 0.0602 |

^1^ Estimated latitude and longitude used for geographical coordinates on **Figure 5**.

Table S8. Mean jackknifed *D* statistics (lower triangular matrix) and standard errors (upper triangular matrix) for pairwise comparisons between cattle breeds from different geographical regions.

A positive *D* statistic indicates that the breed group on the top row displays a higher level of British aurochs admixture than the breed group in the first column. Statistically significant differences indicated with coloured shading according to *P*-value threshold (adjusted for multiple testing using the Benjamini-Hochberg method [8]).

|  | **Britain and Ireland** | **Channel Islands** | **Northern Europe** | **Italy** | **Spanish American** | **Far East** | **West Africa** |
| --- | --- | --- | --- | --- | --- | --- | --- |
| **UK and Ireland** |  | 0.0063 | 0.0038 | 0.0056 | 0.0057 | 0.0074 | 0.0086 |
| **Channel Islands** | 0.0152 |  | 0.0055 | 0.0066 | 0.0074 | 0.0087 | 0.0096 |
| **Northern Europe** | 0.0229 | 0.0080 |  | 0.0039 | 0.0051 | 0.0070 | 0.0082 |
| **Italy** | 0.0588 | 0.0454 | 0.0373 |  | 0.0057 | 0.0072 | 0.0082 |
| **Spanish American** | 0.0836 | 0.0704 | 0.0625 | 0.0258 |  | 0.0081 | 0.0091 |
| **Far East** | 0.0712 | 0.0579 | 0.0502 | 0.0133 | -0.0126 |  | 0.0095 |
| **West Africa** | 0.1974 | 0.1886 | 0.1823 | 0.1487 | 0.1232 | 0.1383 |  |

| adj. *P* ≤ 0.05 | adj. *P* ≤ 0.01 | adj. *P* ≤ 0.001 | adj. *P* ≤ 0.0001 |
| --- | --- | --- | --- |

Table S9. Ensembl VEP (build 78) predicted effects for 263 SNPs and indels potentially selected post-domestication in European *B. taurus* cattle.

| **Ensembl VEP effect** | **Count of effects** |
| --- | --- |
| 3' prime UTR variant | 12 |
| Downstream gene variant | 167 |
| Missense variant | 8 |
| Non coding transcript exon variant | 1 |
| Splice region variant | 1 |
| Synonymous variant | 11 |
| Upstream gene variant | 121 |

Table S10. Details of eight missense mutations potentially selected post-domestication in European *B.* *taurus* cattle.

| **Chrom** | **Position (bp)** | **Ensembl gene ID** | **cDNA position** | **Protein position** | **Amino acids** | **Exon** | **Gene Start (bp)** | **Gene End (bp)** | **Strand** | **Gene symbol** | **Gene name** |
| --- | --- | --- | --- | --- | --- | --- | --- | --- | --- | --- | --- |
| 13 | 338,176 | ENSBTAG00000024891 | 893 | 298 | R/K | 1/1 | 338,154 | 339,068 | -1 | *N/A* | Olfactory receptor |
| 14 | 1,802,266 | ENSBTAG00000026356 | 782 | 232 | A/E | 8/17 | 1,795,351 | 1,804,562 | 1 | *DGAT1* | Diacylglycerol O-acyltransferase 1 |
| 18 | 39,903,447 | ENSBTAG00000047367 | 587 | 196 | H/R | 1/1 | 39,902,861 | 39,905,155 | 1 | *CMTR2* | Cap methyltransferase 2 |
| 7 | 43,292,578 | ENSBTAG00000019925 | 659 | 220 | H/R | 1/4 | 43,291,920 | 43,293,589 | 1 | *N/A* | Olfactory receptor |
| X | 14,324,467 | ENSBTAG00000010463 | 3976 | 1326 | D/N | 22/36 | 14,294,117 | 14,368,145 | -1 | *N/A* | Uncharacterized protein |
| X | 61,108,352 | ENSBTAG00000013760 | 379 | 127 | S/P | 5/42 | 61,054,247 | 61,125,754 | -1 | *COL4A6* | Collagen, type IV, alpha 6 |
| X | 74,933,298 | ENSBTAG00000014485 | 700 | 234 | F/L | 9/21 | 74,869,019 | 74,967,178 | 1 | *POF1B* | Premature ovarian failure, 1B |
| X | 75,151,693 | ENSBTAG00000013077 | 258 | 86 | Q/H | 4/4 | 75,149,333 | 75,151,831 | 1 | *SATL1* | Spermidine/spermine N1-acetyl transferase-like 1 |

Table S11. Top 25-ranking IPA gene categories enriched for 166 out of 193 genes associated with post-domestic DNA sequence variants (VEP analysis) that mapped to molecules in the IPA Knowledge Base (see also Additional File 2).

| **Gene category** | **Functional annotation** | ***P*-value** |
| --- | --- | --- |
| Cancer, Gastrointestinal Disease, Hepatic System Disease | Hyperplasia of hepatocytes | 0.000057 |
| Hereditary Disorder | X-linked hereditary disease | 0.000070 |
| Connective Tissue Disorders, Immunological Disease, Inflammatory Disease, Skeletal and Muscular Disorders | Systemic juvenile idiopathic arthritis | 0.000221 |
| Nervous System Development and Function, Organ Morphology | Size of cerebellar vermis | 0.000338 |
| Developmental Disorder, Neurological Disease | Mental retardation | 0.000351 |
| Neurological Disease | Cognitive impairment | 0.000364 |
| Cell Morphology, Nervous System Development and Function | Morphology of Schwann cells | 0.000511 |
| Hereditary Disorder, Neurological Disease | Dejerine-Sottas disease | 0.000560 |
| Cancer, Endocrine System Disorders | Tumorigenesis of adrenal gland | 0.000560 |
| Cellular Development, Cellular Growth and Proliferation | Proliferation of stomach cancer cell lines | 0.000910 |
| Developmental Disorder, Hereditary Disorder, Metabolic Disease | Fatty acid oxidation disorder | 0.000971 |
| Developmental Disorder, Neurological Disease | Syndromic mental retardation | 0.001060 |
| Cell Cycle | Arrest in G2 phase of ovarian cancer cell lines | 0.001160 |
| Developmental Disorder, Hereditary Disorder, Neurological Disease | Syndromic X-linked mental retardation | 0.001210 |
| Cellular Compromise, Inflammatory Response | Degranulation of leukocyte cell lines | 0.001250 |
| RNA Post-Transcriptional Modification | Unwinding of RNA | 0.001540 |
| Neurological Disease | Peripheral neuropathy | 0.001570 |
| Cancer, Haematological Disease, Immunological Disease, Organismal Injury and Abnormalities | Diffuse large B-cell lymphoma | 0.001670 |
| Post-Translational Modification | Phosphorylation of protein | 0.001690 |
| Cell Morphology, Cellular Assembly and Organization, Cellular Development, Cellular Function and Maintenance, Cellular Growth and Proliferation, Embryonic Development, Nervous System Development and Function, Tissue Development | Growth of dendrites | 0.001760 |
| Cellular Assembly and Organization, Cellular Function and Maintenance | Quantity of cellular protrusions | 0.001830 |
| Cell Morphology, Nervous System Development and Function, Tissue Morphology | Abnormal morphology of myelin sheath | 0.001960 |
| Amino Acid Metabolism, Small Molecule Biochemistry | Catabolism of serine family amino acid | 0.002460 |
| Cellular Development, Cellular Growth and Proliferation | Proliferation of bladder cancer cell lines | 0.002520 |
| Cell Cycle, Cell Morphology, Cellular Assembly and Organization, DNA Replication, Recombination, and Repair | Abnormal morphology of synaptonemal complexes | 0.002990 |

Table S12. The 16 top-ranking significant IPA canonical pathways (*P* ≤ 0.05) enriched for 166 out of 193 genes associated with post-domestic DNA sequence variants (VEP analysis) that mapped to molecules in the IPA Knowledge Base (see also Additional File 2).

| **Functional categories** | ***P*-value** |
| --- | --- |
| Phenylalanine Degradation IV (Mammalian, via Side Chain) | 0.004898 |
| Glutathione Redox Reactions I | 0.007943 |
| Atherosclerosis Signalling | 0.014125 |
| Taurine Biosynthesis | 0.015136 |
| Threonine Degradation II | 0.015136 |
| Glycine Biosynthesis I | 0.015136 |
| Diphthamide Biosynthesis | 0.022387 |
| Hypusine Biosynthesis | 0.022387 |
| Glutamate Degradation II | 0.022387 |
| Aspartate Biosynthesis | 0.022387 |
| Neuregulin Signalling | 0.029512 |
| L-cysteine Degradation I | 0.030200 |
| dTMP De Novo Biosynthesis | 0.037154 |
| Folate Polyglutamylation | 0.037154 |
| Antioxidant Action of Vitamin C | 0.037154 |
| Role of Oct4 in Mammalian Embryonic Stem Cell Pluripotency | 0.047863 |

Table S13. Genes within genomic windows with evidence for positive selection in the *B. taurus* lineage identified using the HKA test (colour-coding highlights uncorrected *P*-value significance thresholds).

| **Chr** | **Gene ID** | **Gene start co-ordinates** | **Gene end co-ordinates** | **Gene symbol** | **Gene description** | ***Window P-*value** |
| --- | --- | --- | --- | --- | --- | --- |
| 1 | ENSBTAG00000006872 | 80,940,147 | 80,940,854 |  | Novel processed pseudogene | ≤ 0.05 |
| 1 | NM_001159316 | 107,774,352 | 107,834,443 | *KPNA4* | Karyopherin alpha 4 (importin alpha 3) | ≤ 0.05 |
| 1 | NM_001080310 | 107,879,882 | 107,892,250 | *TRIM59* | Tripartite motif containing 59 | ≤ 0.05 |
| 1 | NM_001105326 | 107,895,049 | 107,928,238 | *SMC4* | Structural maintenance of chromosomes 4 | ≤ 0.05 |
| 2 | NM_176852 | 72,523,470 | 72,527,172 | *INHBB* | Inhibin, beta B | ≤ 0.01 |
| 2 | NM_001034542 | 72,589,395 | 72,592,508 | *CCT6A* | Chaperonin containing TCP1, subunit 6A (zeta 1) | ≤ 0.05 |
| 2 | NM_001192488 | 73,379,884 | 73,441,557 | *TFCP2L1* | Transcription factor CP2-like 1 | ≤ 0.05 |
| 2 | NM_001191498 | 130,498,485 | 130,852,033 | *EPHB2* | EPH receptor B2 | ≤ 0.05 |
| 2 | ENSBTAG00000048247 | 130,553,870 | 130,553,935 |  | Novel miRNA | ≤ 0.05 |
| 3 | NM_001143860 | 107,179,185 | 107,518,531 | *MACF1* | Microtubule-actin crosslinking factor 1 | ≤ 0.01 |
| 3 | ENSBTAG00000044999 | 107,466,835 | 107,466,971 | *ACA59* | Small nucleolar RNA ACA59 | ≤ 0.05 |
| 3 | ENSBTAG00000043643 | 107,486,376 | 107,486,515 | *U2* | U2 spliceosomal RNA | ≤ 0.05 |
| 4 | NM_001034256 | 78,053,909 | 78,147,627 | *COA1* | Cytochrome C oxidase assembly factor 1 homolog (S. cerevisiae) | ≤ 0.01 |
| 4 | NM_001083422 | 78,160,560 | 78,203,744 | *STK17A* | Serine/threonine kinase 17a | ≤ 0.01 |
| 4 | NM_001034672 | 93,881,395 | 93,901,224 | *TSPAN33* | Tetraspanin 33 | ≤ 0.01 |
| 4 | NM_001192220 | 93,922,580 | 93,946,896 | *SMO* | Smoothened, frizzled family receptor | ≤ 0.01 |
| 4 | NM_001101143 | 93,956,615 | 94,148,561 | *AHCYL2* | Adenosylhomocysteinase-like 2 | ≤ 0.01 |
| 4 | NM_001035475 | 97,318,803 | 97,612,111 | *CHCHD3* | Coiled-coil-helix-coiled-coil-helix domain containing 3 | ≤ 0.01 |
| 8 | ENSBTAG00000020752 | 54,433,665 | 54,468,588 | *CEP78* | Uncharacterized protein | ≤ 0.05 |
| 9 | NM_173990 | 39,903,106 | 39,924,416 | AMD1 | Adenosylmethionine decarboxylase 1 | ≤ 0.05 |
| 9 | NM_001205707 | 39,956,473 | 40,123,498 | CDK19 | Cyclin-dependent kinase 19 | ≤ 0.05 |
| 9 | NM_001076059 | 51,485,364 | 51,561,211 | FBXL4 | F-box and leucine-rich repeat protein 4 | ≤ 0.05 |
| 10 | NM_174305 | 59,227,894 | 59,282,939 | CYP19A1 | Cytochrome P450, family 19, subfamily A, polypeptide 1 | ≤ 0.01 |
| 10 | NR_003052 | 59,305,356 | 59,328,689 | LOC503858 | Aromatase cytochrome P450 pseudogene | ≤ 0.01 |
| 10 | ENSBTAG00000044706 | 70,122,670 | 70,122,796 | SNORA42 | Small nucleolar RNA SNORA42/SNORA80 family | ≤ 0.05 |
| 10 | ENSBTAG00000006341 | 70,145,554 | 70,186,590 | *SLC35F4* | Uncharacterized protein | ≤ 0.05 |
| 10 | NM_001102316 | 85,840,139 | 85,952,853 | *LIN52* | Lin-52 homolog (*C. elegans*) | ≤ 0.05 |
| 11 | NM_001205952 | 36,424,165 | 36,524,445 | *PSME4* | Proteasome (prosome, macropain) activator subunit 4 | ≤ 0.05 |
| 13 | NM_001099141 | 49,550,049 | 49,561,304 | *BMP2* | Bone morphogenetic protein 2 | ≤ 0.05 |
| 15 | NM_001075230 | 39,778,549 | 39,840,041 | *BTBD10* | BTB (POZ) domain containing 10 | ≤ 0.05 |
| 16 | ENSBTAG00000014014 | 43,258,416 | 43,268,285 | *UBIAD1* | Uncharacterized protein | ≤ 0.01 |
| 16 | ENSBTAG00000015325 | 43,275,756 | 43,396,218 | *MTOR* | Uncharacterized protein | ≤ 0.01 |
| 16 | NM_001014909 | 43,328,465 | 43,334,550 | *ANGPTL7* | Angiopoietin-like 7 | ≤ 0.05 |
| 16 | NM_174049 | 45,401,694 | 45,416,462 | *ENO1* | Enolase 1, (alpha) | ≤ 0.05 |
| 16 | ENSBTAG00000042245 | 45,429,775 | 45,429,878 | *U6* | U6 spliceosomal RNA | ≤ 0.05 |
| 16 | ENSBTAG00000018272 | 45,621,457 | 45,879,645 | *RERE* | Uncharacterized protein | ≤ 0.05 |
| 16 | NM_001192339 | 45,886,756 | 45,908,565 | *SLC45A1* | Solute carrier family 45, member 1 | ≤ 0.05 |
| 17 | NM_001193067 | 57,398,013 | 57,422,729 | *SH2B3* | SH2B adaptor protein 3 | ≤ 0.05 |
| 18 | NM_173991 | 53,040,104 | 53,042,792 | *APOE* | Apolipoprotein E | ≤ 0.05 |
| 18 | NM_001034807 | 53,054,656 | 53,057,040 | *APOC4* | Apolipoprotein C-IV | ≤ 0.05 |
| 18 | NM_001102380 | 53,057,716 | 53,059,957 | *APOC2* | Apolipoprotein C-II | ≤ 0.05 |
| 18 | NM_001046613 | 53,066,527 | 53,093,157 | *CLPTM1* | Cleft lip and palate associated transmembrane protein 1 | ≤ 0.05 |
| 19 | NM_001015553 | 34,914,350 | 34,929,470 | *SHMT1* | Serine hydroxymethyltransferase 1 (soluble) | ≤ 0.05 |
| 19 | ENSBTAG00000017090 | 34,932,550 | 34,960,757 | *SMCR8* | Uncharacterized protein | ≤ 0.05 |
| 19 | ENSBTAG00000017233 | 52,947,903 | 53,006,764 | *RNF213* | Uncharacterized protein | ≤ 0.01 |
| 19 | NM_001014866 | 53,023,435 | 53,041,077 | *SLC26A11* | Solute carrier family 26, member 11 | ≤ 0.01 |
| 19 | NM_001102189 | 53,041,241 | 53,048,385 | *SGSH* | N-sulfoglucosamine sulfohydrolase | ≤ 0.01 |
| 19 | NM_001205901 | 53,048,428 | 53,069,156 | *CARD14* | Caspase recruitment domain family, member 14 | ≤ 0.01 |
| 19 | NM_001046188 | 53,085,462 | 53,097,208 | *EIF4A3* | Eukaryotic translation initiation factor 4A3 | ≤ 0.01 |
| 19 | NM_173913 | 53,100,964 | 53,113,264 | *GAA* | Glucosidase, alpha; acid | ≤ 0.01 |
| 19 | ENSBTAG00000030186 | 53,108,151 | 53,108,444 | *USMG5* | Up-regulated during skeletal muscle growth protein 5 | ≤ 0.01 |
| 19 | NM_001206271 | 53,181,378 | 53,246,057 | *TBC1D16* | TBC1 domain family, member 16 | ≤ 0.05 |
| 20 | NM_001206580 | 29,121,454 | 29,567,228 | *HCN1* | Hyperpolarization activated cyclic nucleotide-gated potassium channel 1 | ≤ 0.01 |
| 25 | NM_001075649 | 2,867,519 | 2,884,578 | *NAA60* | N(alpha)-acetyltransferase 60, NatF catalytic subunit | ≤ 0.05 |
| 25 | NM_001206478 | 2,885,983 | 2,887,757 | *C25H16orf90* | Chromosome 25 open reading frame, human C16orf90 | ≤ 0.05 |
| 25 | NM_001105450 | 2,894,809 | 2,924,574 | *CLUAP1* | Clusterin associated protein 1 | ≤ 0.01 |
| 25 | ENSBTAG00000004509 | 2,958,710 | 2,977,159 | *SLX4* | Uncharacterized protein | ≤ 0.01 |
| 25 | NR_031246 | 2,988,662 | 2,988,738 | *MIR2383* | microRNA | ≤ 0.05 |
| 25 | NM_174534 | 2,992,453 | 2,995,476 | *DNASE1* | Deoxyribonuclease I | ≤ 0.05 |
| 25 | NM_001038675 | 2,995,414 | 3,044,580 | *TRAP1* | TNF receptor-associated protein 1 | ≤ 0.05 |
| 25 | NM_001164022 | 3,050,213 | 3,169,185 | *CREBBP* | CREB binding protein | ≤ 0.05 |
| 27 | ENSBTAG00000033381 | 32,417,551 | 32,418,888 |  | Uncharacterized protein | ≤ 0.01 |
| 28 | NM_001192127 | 25,268,531 | 25,328,274 | *STOX1* | Storkhead box 1 | ≤ 0.05 |
| 28 | NM_001206053 | 25,334,951 | 25,368,167 | *DDX50* | DEAD (Asp-Glu-Ala-Asp) box polypeptide 50 | ≤ 0.01 |
| 28 | NM_001083527 | 25,376,358 | 25,399,769 | *DDX21* | DEAD (Asp-Glu-Ala-Asp) box polypeptide 21 | ≤ 0.01 |
| X | ENSBTAG00000021344 | 16,857,812 | 16,860,502 | *USP26* | Ubiquitin carboxyl-terminal hydrolase | ≤ 0.05 |
| X | ENSBTAG00000002427 | 23,837,351 | 23,838,406 |  | Novel pseudogene | ≤ 0.05 |
| X | ENSBTAG00000021713 | 33,285,824 | 33,402,706 | *MAMLD1* | Uncharacterized protein | ≤ 0.05 |
| X | NM_001206425 | 33,460,692 | 33,552,556 | *MTM1* | Myotubularin 1 | ≤ 0.05 |
| X | NM_001206067 | 33,558,426 | 33,635,000 | *MTMR1* | Myotubularin related protein 1 | ≤ 0.05 |
| X | NM_001083758 | 33,636,322 | 33,739,781 | *CD99L2* | CD99 molecule-like 2 | ≤ 0.05 |
| X | NM_174052 | 34,423,521 | 34,429,968 | *FATE1* | Foetal and adult testis expressed 1 | ≤ 0.05 |
| X | NM_001001139 | 34,433,640 | 34,450,338 | *CNGA2* | Cyclic nucleotide gated channel alpha 2 | ≤ 0.05 |
| X | NM_001192639 | 35,299,888 | 35,314,450 | *GABRQ* | Gamma-aminobutyric acid (GABA) receptor, theta | ≤ 0.05 |
| X | NM_001038515 | 35,382,933 | 35,386,969 | *CETN2* | Centrin, EF-hand protein, 2 | ≤ 0.05 |
| X | NM_001035482 | 35,387,261 | 35,413,263 | *NSDHL* | NAD(P) dependent steroid dehydrogenase-like | ≤ 0.05 |
| X | NM_001075790 | 38,708,010 | 38,788,208 | *BRCC3* | BRCA1/BRCA2-containing complex, subunit 3 | ≤ 0.05 |
| X | NM_001078151 | 38,788,661 | 38,800,168 | *MTCP1NB* | Mature T-cell proliferation 1 neighbour | ≤ 0.05 |
| X | NM_001195623 | 38,795,144 | 38,796,283 | *CMC4* | Mature T-cell proliferation 1 | ≤ 0.05 |
| X | NM_174338 | 38,815,310 | 38,838,616 | *FUNDC2* | FUN14 domain containing 2 | ≤ 0.05 |
| X | NM_001145508 | 38,838,454 | 38,982,287 | *F8* | Coagulation factor VIII, procoagulant component | ≤ 0.01 |
| X | NM_001075484 | 39,003,630 | 39,036,437 | *MPP1* | Membrane protein, palmitoylated 1, 55kDa | ≤ 0.01 |
| X | NM_001105395 | 39,037,543 | 39,049,378 | *DKC1* | Dyskeratosis congenita 1, dyskerin | ≤ 0.01 |
| X | NR_031274 | 39,043,972 | 39,044,054 | *MIR664* | microRNA | ≤ 0.05 |
| X | NM_001192307 | 39,064,088 | 39,147,727 | *GAB3* | GRB2-associated binding protein 3 | ≤ 0.05 |
| X | ENSBTAG00000014169 | 51,118,208 | 51,246,606 | *PCDH19* | Uncharacterized protein | ≤ 0.05 |
| X | NM_001045930 | 60,925,305 | 60,958,778 | *VSIG1* | V-set and immunoglobulin domain containing 1 | ≤ 0.05 |
| X | NM_001046406 | 60,966,170 | 60,973,473 | *PSMD10* | Proteasome (prosome, macropain) 26S subunit, non-ATPase, 10 | ≤ 0.05 |
| X | NR_031278 | 61,016,866 | 61,016,930 | *MIR2284E* | microRNA mir-2284e | ≤ 0.05 |
| X | NM_001001171 | 61,017,761 | 61,050,945 | *ATG4A* | ATG4 autophagy related 4 homolog A (S. cerevisiae) | ≤ 0.05 |
| X | ENSBTAG00000047549 | 69,381,843 | 69,381,949 | *U6* | U6 spliceosomal RNA | ≤ 0.05 |
| X | NM_001205926 | 69,497,490 | 69,691,979 | *RPS6KA6* | Ribosomal protein S6 kinase, 90kDa, polypeptide 6 | ≤ 0.05 |
| X | ENSBTAG00000016230 | 71,002,059 | 71,091,449 | *BRWD3* | Uncharacterized protein | ≤ 0.05 |
| X | ENSBTAG00000048181 | 71,160,481 | 71,160,587 | *U6* | U6 spliceosomal RNA | ≤ 0.05 |
| X | ENSBTAG00000014485 | 74,869,019 | 74,967,178 | *POF1B* | Uncharacterized protein | ≤ 0.05 |
| X | NM_001205925 | 74,972,457 | 74,999,240 | *ZNF711* | Zinc finger protein 711 | ≤ 0.05 |
| X | ENSBTAG00000013077 | 75,149,333 | 75,151,831 | *SATL1* | Uncharacterized protein | ≤ 0.05 |
| X | NM_001035031 | 75,160,532 | 75,265,102 | *APOOL* | Apolipoprotein O-like | ≤ 0.05 |
| X | ENSBTAG00000042136 | 87,243,997 | 87,244,103 | *U6* | U6 spliceosomal RNA | ≤ 0.05 |
| X | NM_001193203 | 90,149,332 | 90,230,185 | *CHST7* | Carbohydrate (N-acetylglucosamine 6-O) sulfotransferase 7 | ≤ 0.05 |
| X | ENSBTAG00000003290 | 90,182,201 | 90,265,892 | *SLC9A7* | Sodium/hydrogen exchanger | ≤ 0.05 |
| X | ENSBTAG00000044384 | 93,637,743 | 93,637,845 | *U6* | U6 spliceosomal RNA | ≤ 0.05 |
| X | NM_001015604 | 95,933,519 | 95,953,706 | *GPR173* | G protein-coupled receptor 173 | ≤ 0.05 |
| X | ENSBTAG00000017783 | 95,957,461 | 95,964,356 | *TSPYL2* | Uncharacterized protein | ≤ 0.05 |
| X | NM_174614 | 96,218,219 | 96,252,806 | *SMC1A* | Structural maintenance of chromosomes 1A | ≤ 0.05 |
| X | NM_001075585 | 96,253,140 | 96,266,987 | *RIBC1* | RIB43A domain with coiled-coils 1 | ≤ 0.01 |
| X | NM_174334 | 96,267,143 | 96,269,467 | *HSD17B10* | hydroxysteroid (17-beta) dehydrogenase 10 | ≤ 0.01 |
| X | NM_001110004 | 96,362,880 | 96,520,246 | *HUWE1* | HECT, UBA and WWE domain containing 1 | ≤ 0.01 |
| X | NR_031360 | 96,382,627 | 96,382,746 | *MIR98* | microRNA | ≤ 0.01 |
| X | NR_031268 | 96,383,531 | 96,383,614 | *MIRLET7F-2* | microRNA | ≤ 0.01 |
| X | NM_001128500 | 130,423,302 | 130,712,929 | *SH3KBP1* | SH3-domain kinase binding protein 1 | ≤ 0.05 |
| X | ENSBTAG00000019855 | 130,730,265 | 130,875,653 | *MAP3K15* | Uncharacterized protein | ≤ 0.05 |
| X | ENSBTAG00000009545 | 131,866,408 | 131,954,986 | *BT.48801* | Serine/threonine-protein phosphatase | ≤ 0.05 |
| X | NM_001145232 | 144,430,888 | 144,489,539 | *TBL1X* | Transducin (beta)-like 1X-linked | ≤ 0.05 |

Table S14. Top 25-ranking IPA gene categories enriched for 96 unique genes mapping to IPA Knowledge Base molecules and within genomic windows under positive selection in the *B. taurus* lineage according to the HKA analysis.

| **Gene category** | **Functional annotation** | ***P*-value** |
| --- | --- | --- |
| Hereditary Disorder | X-linked hereditary disease | 0.000005 |
| Cell Morphology, Haematological System Development and Function, Inflammatory Response | M2 polarization of bone marrow-derived macrophages | 0.000019 |
| Developmental Disorder, Hereditary Disorder, Neurological Disease | X-linked mental retardation | 0.000036 |
| Cancer, Organismal Injury and Abnormalities, Reproductive System Disease | Hormone receptor positive advanced breast cancer | 0.000058 |
| Developmental Disorder, Neurological Disease | Syndromic mental retardation | 0.000059 |
| Cellular Development, Cellular Growth and Proliferation, Skeletal and Muscular System Development and Function, Tissue Development | Proliferation of PASMC cells | 0.000065 |
| Cell Morphology, Cellular Function and Maintenance, Renal and Urological System Development and Function | Autophagy of kidney cell lines | 0.000159 |
| Neurological Disease | Cognitive impairment | 0.000172 |
| Developmental Disorder, Hereditary Disorder, Neurological Disease | Syndromic X-linked mental retardation | 0.000254 |
| Carbohydrate Metabolism, Small Molecule Biochemistry | Metabolism of proteoglycan | 0.000282 |
| Cancer, Organismal Injury and Abnormalities, Reproductive System Disease | Estrogen receptor positive breast cancer | 0.000288 |
| RNA Post-Transcriptional Modification | Modification of RNA fragment | 0.000288 |
| Haematological System Development and Function, Haematopoiesis, Tissue Morphology | Quantity of erythroid cells | 0.000288 |
| Organ Development | Function of muscle | 0.000324 |
| Carbohydrate Metabolism, Lipid Metabolism, Small Molecule Biochemistry | Dephosphorylation of phosphatidylinositol-3-phosphate | 0.000401 |
| Organ Morphology, Skeletal and Muscular System Development and Function | Mass of skeletal muscle | 0.000529 |
| Carbohydrate Metabolism, Lipid Metabolism, Small Molecule Biochemistry | Hydrolysis of phosphatidylinositol-3-phosphate | 0.000534 |
| Cellular Assembly and Organization | Quantity of autophagosomes | 0.000534 |
| Embryonic Development, Nervous System Development and Function, Organ Development, Organismal Development, Tissue Development | Regionalization of telencephalon | 0.000534 |
| RNA Post-Transcriptional Modification | Unwinding of RNA | 0.000534 |
| DNA Replication, Recombination, and Repair | Repair of DNA | 0.000591 |
| Organismal Development | Lean body mass | 0.000605 |
| Cancer, Organismal Injury and Abnormalities, Reproductive System Disease | Hormone receptor positive breast carcinoma | 0.000633 |
| Reproductive System Development and Function, Tissue Morphology | Abnormal morphology of epigonadal fat pad | 0.000684 |
| Cancer, Organismal Injury and Abnormalities, Reproductive System Disease | Metastatic hormone receptor positive breast cancer | 0.000684 |

Table S15. The top 25-ranking IPA canonical pathways enriched for 96 unique genes mapping to IPA Knowledge Base molecules and within genomic windows under positive selection in the *B. taurus* lineage according to the HKA analysis.

| **Gene categories** | ***P*-value** |
| --- | --- |
| FXR/RXR Activation | 0.000251 |
| Production of Nitric Oxide and Reactive Oxygen Species in Macrophages | 0.001230 |
| Clathrin-mediated Endocytosis Signalling | 0.001380 |
| Estrogen-Dependent Breast Cancer Signalling | 0.002630 |
| FLT3 Signalling in Hematopoietic Progenitor Cells | 0.004365 |
| TGF-β Signalling | 0.006761 |
| Spermine Biosynthesis | 0.008913 |
| Glycine Biosynthesis I | 0.008913 |
| Estrogen Biosynthesis | 0.011749 |
| NGF Signalling | 0.012023 |
| Spermidine Biosynthesis I | 0.013183 |
| Role of IL-17F in Allergic Inflammatory Airway Diseases | 0.016218 |
| LPS/IL-1 Mediated Inhibition of RXR Function | 0.016218 |
| LXR/RXR Activation | 0.016596 |
| Atherosclerosis Signalling | 0.017378 |
| dTMP De Novo Biosynthesis | 0.021878 |
| Folate Polyglutamylation | 0.021878 |
| IL-12 Signalling and Production in Macrophages | 0.022387 |
| CNTF Signalling | 0.022387 |
| Wnt/Ca+ pathway | 0.025704 |
| Zymosterol Biosynthesis | 0.026303 |
| ATM Signalling | 0.028184 |
| Superpathway of Serine and Glycine Biosynthesis I | 0.030903 |
| ERK5 Signalling | 0.031623 |
| Folate Transformations I | 0.038905 |

Table S16. Number of genes targeted by the five miRNAs containing polymorphisms (in the mature miRNA sequence) between *B. primigenius* and *B. taurus.*

| **miRNA** | ***B. taurus*** | ***B. primigenius*** | **Intersection** |
| --- | --- | --- | --- |
| miR-769 | 573 | 572 | 570 |
| miR-940 | 1246 | 1241 | 1233 |
| miR-2391 | 349 | 344 | 343 |
| miR-2469 | 712 | 705 | 701 |
| miR-2893 | 1148 | 1077 | 610 |

Table S17. *B. taurus* genes selected for Agilent SureSelect target enrichment.

| **Gene Name** | **Ensembl Gene ID** | **Gene Start (bp)** | **Gene End (bp)** | **Gene description** | **Chr** |
| --- | --- | --- | --- | --- | --- |
| *ABCG2* | ENSBTAG00000017704 | 37,913,110 | 38,030,583 | ATP-binding cassette, sub-family G (WHITE), member 2 | 6 |
| *AGRP* | ENSBTAG00000014556 | 35,205,885 | 35,206,635 | Agouti related protein homolog (mouse) | 18 |
| *AHSG* | ENSBTAG00000000522 | 81,202,132 | 81,209,114 | Alpha-2-HS-glycoprotein | 1 |
| *ALB* | ENSBTAG00000017121 | 90,232,762 | 90,251,126 | Albumin | 6 |
| *APOA1* | ENSBTAG00000002258 | 27,932,200 | 27,934,085 | Apolipoprotein A-I (APOA1) | 15 |
| *APOE* | ENSBTAG00000010123 | 53,040,105 | 53,042,792 | Apolipoprotein E | 18 |
| *ARHGDIA* | ENSBTAG00000030209 | 51,633,694 | 51,637,708 | Rho GDP dissociation inhibitor alpha | 19 |
| *ASIP* | ENSBTAG00000034077 | 64,234,645 | 64,239,783 | Agouti signalling protein | 13 |
| *ATRN* | ENSBTAG00000003848 | 52,003,104 | 52,192,979 | Attractin | 13 |
| *CAPN1* | ENSBTAG00000010230 | 44,064,429 | 44,089,990 | Calpain 1, (mu/I) large subunit | 29 |
| *CAPN2* | ENSBTAG00000012778 | 27,781,671 | 27,840,009 | Calpain 2, (m/II) large subunit | 16 |
| *CAPN3* | ENSBTAG00000008868 | 37,828,797 | 37,885,860 | Calpain 3, (p94) | 10 |
| *CAST* | ENSBTAG00000000874 | 98,444,979 | 98,581,253 | Calpastatin | 7 |
| *CD14* | ENSBTAG00000015032 | 53,446,106 | 53,448,979 | CD14 molecule | 7 |
| *CD40* | ENSBTAG00000020736 | 75,563,518 | 75,574,215 | CD40 molecule, TNF receptor superfamily member 5 | 13 |
| *CD46* | ENSBTAG00000005397 | 77,483,017 | 77,522,665 | CD46 molecule, complement regulatory protein | 16 |
| *CSN1S1* | ENSBTAG00000007695 | 87,141,556 | 87,159,096 | Casein alpha s1 | 6 |
| *CSN1S2* | ENSBTAG00000005005 | 87,262,457 | 87,280,936 | Casein alpha-S2 | 6 |
| *CSN2* | ENSBTAG00000002632 | 87,179,502 | 87,188,025 | Casein beta | 6 |
| *CSN3* | ENSBTAG00000039787 | 87,378,398 | 87,392,750 | Casein kappa | 6 |
| *DBH* | ENSBTAG00000004508 | 104,554,824 | 104,572,619 | Dopamine beta-hydroxylase (dopamine beta-monooxygenase) | 11 |
| *DCT* | ENSBTAG00000002300 | 69,502,941 | 69,544,658 | Dopachrome tautomerase | 12 |
| *DGAT1* | ENSBTAG00000026356 | 1,795,351 | 1,804,562 | Diacylglycerol O-acyltransferase 1 | 14 |
| *DRD1* | ENSBTAG00000047719 | 5,653,453 | 5,655,684 | Dopamine receptor D1 | 10 |
| *DRD2* | ENSBTAG00000010860 | 24,303,204 | 24,316,174 | Dopamine receptor D2 | 15 |
| *EDNRB* | ENSBTAG00000005299 | 53,310,604 | 53,407,024 | Endothelin receptor type B | 12 |
| *FASN* | ENSBTAG00000015980 | 51,384,922 | 51,403,614 | Fatty acid synthase | 19 |
| *GH1* | ENSBTAG00000017220 | 48,768,618 | 48,772,014 | Growth hormone 1 | 19 |
| *GHR* | ENSBTAG00000001335 | 31,890,736 | 32,199,996 | Growth hormone receptor | 20 |
| *GNAS* | ENSBTAG00000017475 | 58,010,287 | 58,049,012 | Guanine nucleotide-binding protein G(s) subunit alpha isoforms short | 13 |
| *GNAS* | ENSBTAG00000047223 | 58,019,524 | 58,049,012 | Neuroendocrine secretory protein 55 LSAL tetrapeptide GAIPIRRH peptide | 13 |
| *GNB1* | ENSBTAG00000000215 | 52,106,960 | 52,184,132 | Guanine nucleotide binding protein (G protein), beta polypeptide 1 | 16 |
| *GRB10* | ENSBTAG00000017086 | 5,104,899 | 5,244,348 | Growth factor receptor-bound protein 10 | 4 |
| *GSTT1* | ENSBTAG00000040298 | 73,300,145 | 73,307,793 | Glutathione S-transferase theta 1 | 17 |
| *GSTT3* | ENSBTAG00000008587 | 73,309,907 | 73,315,353 | Glutathione S-transferase, theta 3 | 17 |
| *HSTN* | ENSBTAG00000048250 | 87,189,736 | 87,204,439 | Histatherin | 6 |
| *HTR2A* | ENSBTAG00000013498 | 16,823,524 | 16,889,281 | 5-hydroxytryptamine (serotonin) receptor 2A | 12 |
| *IFNG* | ENSBTAG00000012529 | 45,830,158 | 45,834,981 | Interferon, gamma | 5 |
| *IGF1R* | ENSBTAG00000021527 | 8,208,822 | 8,268,093 | Insulin-like growth factor 1 receptor | 21 |
| *IGF2* | ENSBTAG00000013066 | 50,046,626 | 50,065,230 | Insulin-like growth factor 2 | 29 |
| *IGF2R* | ENSBTAG00000002402 | 97,638,172 | 97,740,003 | Cation-independent mannose-6-phosphate receptor precursor | 9 |
| *IL1B* | ENSBTAG00000001321 | 46,410,277 | 46,418,787 | Interleukin 1, beta | 11 |
| *IL6* | ENSBTAG00000014921 | 31,578,311 | 31,582,667 | Interleukin 6 | 4 |
| *INS* | ENSBTAG00000013003 | 50,036,101 | 50,037,262 | Insulin | 29 |
| *KIT* | ENSBTAG00000002699 | 71,796,318 | 71,917,431 | v-kit Hardy-Zuckerman 4 feline sarcoma viral oncogene homolog | 6 |
| *LALBA* | ENSBTAG00000005859 | 31,347,861 | 31,349,882 | Lactalbumin, alpha | 5 |
| *LGB* | ENSBTAG00000014678 | 103,301,664 | 103,306,381 | Progestagen-associated endometrial protein | 11 |
| *LTF* | ENSBTAG00000001292 | 53,521,906 | 53,556,089 | Lactotransferrin | 22 |
| *MAOA* | ENSBTAG00000016206 | 105,380,194 | 105,462,564 | Monoamine oxidase A | X |
| *MC1R* | ENSBTAG00000023731 | 14,757,332 | 14,759,082 | Melanocortin 1 receptor | 18 |
| *MGRN1* | ENSBTAG00000018999 | 3,754,935 | 3,791,613 | Mahogunin ring finger 1, E3 ubiquitin protein ligase | 25 |
| *MITF* | ENSBTAG00000006679 | 31,735,990 | 31,769,463 | Microphthalmia-associated transcription factor | 22 |
| *MSR1* | ENSBTAG00000002885 | 19,976,214 | 20,056,927 | Macrophage scavenger receptor 1 | 27 |
| *MSTN* | ENSBTAG00000011808 | 6,213,566 | 6,220,196 | Myostatin | 2 |
| *NFKB1* | ENSBTAG00000020270 | 23,557,311 | 23,679,508 | Nuclear factor of kappa light polypeptide gene enhancer in B-cells 1 | 6 |
| *NPY* | ENSBTAG00000004503 | 72,068,358 | 72,075,482 | Neuropeptide Y | 4 |
| *PCYOX1* | ENSBTAG00000002783 | 68,560,573 | 68,572,075 | Prenylcysteine oxidase 1 | 11 |
| *PEG3* | ENSBTAG00000023338 | 64,270,787 | 64,282,112 | Paternally-expressed gene 3 | 18 |
| *PEG10* | ENSBTAG00000038093 | 11,912,009 | 11,921,418 | Paternally expressed gene 10 | 4 |
| *PGLYRP1* | ENSBTAG00000002635 | 53,946,097 | 53,948,160 | Peptidoglycan recognition protein 1 | 18 |
| *PIGR* | ENSBTAG00000019798 | 4,533,505 | 4,551,569 | Polymeric immunoglobulin receptor | 16 |
| *PMCH* | ENSBTAG00000013008 | 66,359,938 | 66,361,103 | Pro-melanin-concentrating hormone | 5 |
| *POMC* | ENSBTAG00000007897 | 74,109,410 | 74,116,893 | Proopiomelanocortin | 11 |
| *PRNP* | ENSBTAG00000027937 | 47,400,413 | 47,418,507 | Prion protein | 13 |
| *PTX3* | ENSBTAG00000009012 | 111,027,804 | 111,033,868 | Pentraxin 3, long | 1 |
| *R3HDM1* | ENSBTAG00000013302 | 61,969,459 | 62,130,680 | R3H domain containing 1 | 2 |
| *RAB11B* | ENSBTAG00000002475 | 18,249,534 | 18,259,141 | RAB11B, member RAS oncogene family | 7 |
| *RAB18* | ENSBTAG00000009871 | 37,303,894 | 37,331,999 | RAB18, member RAS oncogene family | 13 |
| *RAB3A* | ENSBTAG00000010635 | 4,944,325 | 4,950,010 | RAB3A, member RAS oncogene family | 7 |
| *RAB3C* | ENSBTAG00000007306 | 20,448,540 | 20,741,101 | RAB3C, member RAS oncogene family | 20 |
| *RAP1B* | ENSBTAG00000008967 | 45,355,308 | 45,399,175 | RAP1B, member of RAS oncogene family | 5 |
| *RASGRF1* | ENSBTAG00000019940 | 25,714,965 | 25,816,371 | Ras protein-specific guanine nucleotide-releasing factor 1 | 21 |
| *RTL1* | ENSBTAG00000046585 | 67,427,514 | 67,431,509 | Retrotransposon-like 1 | 21 |
| *S100A2* | ENSBTAG00000037651 | 16,869,280 | 16,872,613 | S100 calcium binding protein A2 | 3 |
| *SAR1A* | ENSBTAG00000016858 | 26,542,608 | 26,553,908 | SAR1 homolog A (*S. cerevisiae*) | 28 |
| *SAR1B* | ENSBTAG00000021226 | 47,717,606 | 47,747,828 | SAR1 homolog B (*S. cerevisiae*) | 7 |
| *SERGEF* | ENSBTAG00000005340 | 35,019,670 | 35,267,343 | Secretion regulating guanine nucleotide exchange factor | 15 |
| *SLAMF1* | ENSBTAG00000007927 | 9,090,441 | 9,129,487 | Signalling lymphocytic activation molecule family member 1 | 3 |
| *SLAMF6* | ENSBTAG00000014368 | 9,272,217 | 9,293,445 | SLAM family member 6 | 3 |
| *SLAMF7* | ENSBTAG00000001197 | 8,969,737 | 8,985,751 | SLAM family member 7 | 3 |
| *SLC24A5* | ENSBTAG00000004854 | 62,467,611 | 62,489,654 | Solute carrier family 24, member 5 | 10 |
| *SLC6A4* | ENSBTAG00000019349 | 21,852,431 | 21,872,404 | Solute carrier family 6 (neurotransmitter transporter, serotonin), member 4 | 19 |
| *STAT1* | ENSBTAG00000007867 | 79,888,206 | 79,973,705 | Signal transducer and activator of transcription 1, 91kDa | 2 |
| *STAT3* | ENSBTAG00000021523 | 43,056,660 | 43,132,624 | Signal transducer and activator of transcription 3 | 19 |
| *STAT5A* | ENSBTAG00000009496 | 43,033,597 | 43,054,075 | Signal transducer and activator of transcription 5A | 19 |
| *STATH* | ENSBTAG00000024683 | 87,229,656 | 87,239,005 | Statherin | 6 |
| *TBC1D1* | ENSBTAG00000013699 | 58,848,558 | 59,065,295 | TBC1 (tre-2/USP6, BUB2, cdc16) domain family, member 1 | 6 |
| *TICAM1* | ENSBTAG00000019966 | 20,547,964 | 20,550,264 | Toll-like receptor adaptor molecule 1 | 7 |
| *TLR10* | ENSBTAG00000014042 | 59,670,233 | 59,677,223 | Toll-like receptor 10 | 6 |
| *TLR2* | ENSBTAG00000008008 | 3,949,710 | 3,963,032 | Toll-like receptor 2 | 17 |
| *TLR4* | ENSBTAG00000006240 | 108,828,899 | 108,839,910 | Toll-like receptor 4 | 8 |
| *TLR6* | ENSBTAG00000014031 | 59,687,276 | 59,706,713 | Toll-like receptor 6 | 6 |
| *TYR* | ENSBTAG00000011813 | 6,351,877 | 6,462,240 | Tyrosinase (oculocutaneous albinism IA) | 29 |
| *TYRP1* | ENSBTAG00000020985 | 31,710,698 | 31,726,956 | Tyrosinase-related protein 1 | 8 |
| *YWHAB* | ENSBTAG00000016846 | 74,018,886 | 74,040,819 | Tyrosine 3-monooxygenase/tryptophan 5-monooxygenase activation protein, beta polypeptide | 13 |
| *ZIM2* | ENSBTAG00000011664 | 64,175,124 | 64,177,906 | Zinc finger, imprinted 2 | 18 |

Supplementary Methods

1. Aurochs CPC98 humerus bone DNA extraction

All DNA extraction, purification and Illumina^®^ sequencing library preparation steps were performed in a dedicated ancient DNA laboratory located at the Department of Genetics, Trinity College, Dublin, Ireland. The C1, C2 and C3 DNA extracts were prepared from three independently generated bone powder samples obtained from different locations on the aurochs CPC98 humerus. The methods used for bone powder processing and DNA extraction and purification for the C1–C3 samples have been fully described by us elsewhere [9]. The C1–C3 DNA extracts were used for Illumina^®^ single-read library preparation [9].

For the present study, three additional DNA extracts (C4, C5 and C6) were generated from three independent bone powder samples obtained from different sites on the CPC98 humerus. Powdered bone samples were prepared for DNA extraction using a modified procedure previously described by our group [10-12]. In brief, all laboratory consumables and reagents (with the exception of the Proteinase K) were UV-irradiated prior to use. 200−300 mg bone powder was incubated overnight at 55°C in 1 ml DNA extraction buffer (20 mM Tris-HCl, pH 8.0; 500 mM EDTA, pH 8.0; 0.5% vol/vol Sarkosyl; 200 µg/ml Proteinase K), after which the incubation temperature was reduced to 37°C and the bone powder was incubated for an additional 24 hours. After the incubation period, the samples were centrifuged for 15 min at 12,000 × *g* and the supernatant transferred to a Centricon^®^ YM-30 filter contained in a 10 ml tube. 3 ml 10 mM Tris-HCl (pH 8.0) was added to the Centricon^®^ filter and centrifuged for 30 min at 2,500 rpm. Once centrifuged, the flow-through was discarded and the filter was recentrifuged until almost all the supernatant had passed through the filter. The filter was then washed once by adding 3 ml 10 mM Tris-HCl (pH 8.0) to the filter followed by centrifugation for 30 min at 2,500 rpm. After this wash step, the volume retained above the filter (~200 µl) was transferred to a fresh DNase- and RNase-free 2 ml screw cap tube and subjected to further purification using a Qiagen MinElute kit as per the manufacturer’s instructions, apart from an additional PE wash step. The DNA for each CPC98 extract was eluted in in 100 µl 1× Tris-EDTA buffer, which was subsequently divided into three separate ~30 µl aliquots (labelled C4_1-3_, C5_1-3_ and C6_1-3_); all nine DNA aliquots were used to generate paired-end CPC98 Illumina^®^ sequencing libraries. **Figure S1** shows a schema detailing the laboratory procedures used for the preparation of the C1−C6 DNA extracts, the Illumina^®^ sequencing libraries generated, the sequencing platforms used, and the sequencing centre locations.

1. High-throughput sequencing of aurochs single-read libraries

Pooled Illumina^®^ sequencing libraries C1−C3 generated from three aliquots of each extract were previously sequenced and analysed by us using the Illumina^®^ Genome Analyzer IIx (GA IIx) platform [9]. For the present study, additional sequencing data were generated from these libraries using the Illumina^®^ HiSeq 2000 platform.

Previous analysis of the C1 and C2 sequencing libraries revealed that 27.06% of reads from these libraries consisted largely or entirely of Illumina^®^ GA sequencing adaptor sequence [9]. Consequently, for the present study, aliquots of the C1 and C2 pooled single-read libraries were used for gel purification using a 4% agarose gel stained with 0.5 µg/ml ethidium bromide (Invitrogen/Life Technologies Corp., Paisley, UK). This step was followed by DNA extraction using a QIAquick gel extraction kit (Qiagen Ltd., Crawley, UK) to remove excess Illumina^®^ PCR adaptor dimer fragments. The C1 and C2 libraries were analysed and quantified post-gel purification on an Agilent Bioanalyzer using an Agilent DNA 7500 Labchip (Agilent Technologies, Santa Clara, CA, USA). **Figure S12** shows the gel purification procedure and Agilent electropherograms pre- and post-gel purification for the C1 and C2 libraries. Sequencing of the modified C1 and C2 pooled libraries was performed at the Beijing Genome Institute (BGI), Shenzhen, China, using 49 bp reads on the Illumina^®^ HiSeq 2000 sequencing platform.

1. Paired-end aurochs library preparation and sequencing
   1. Blunt end-repair of aurochs DNA extracts

Nine purified CPC98 DNA extract aliquots (C4_1-3_, C5_1-3_ and C6_1-3_) were used for Illumina^®^ paired-end DNA sequencing according to the NEBNext^®^ DNA Library Prep Master Mix Set for Illumina^®^ (New England Biolabs Inc. [NEB], Ipswich, MA, USA). For this procedure, blunt end-repair was performed on each DNA extract. This involved the inclusion of 30 µl of aurochs DNA in a 100 µl final reaction mixture containing 1× T4 DNA ligase buffer with 1 mM dATP (NEB), 400 µM of each dNTP (Invitrogen/Life Technologies), 15 U T4 DNA polymerase (NEB), 5 U DNA Polymerase I Large (Klenow) Fragment (NEB) and 50 U T4 polynucleotide kinase (NEB). Reaction mixtures were incubated at 20°C for 30 min, after which end-repaired DNA was purified using a QIAquick PCR Purification Kit (Qiagen) and eluted in 32 µl of elution buffer according to manufacturer’s instructions.

- 1. Generation of 3'-dATP overhangs on the end-repaired aurochs DNA extracts

To facilitate Illumina^®^ GA adaptor ligation, a single ‘A’ base was added to the 3'-ends of the blunt-end repaired aurochs DNA extracts. 32 µl of purified blunt end-repaired aurochs extract DNA was included in a final 50 µl reaction mixture containing: 1 × Klenow fragment buffer (NEB), 200 µM dATP (Invitrogen/Life Technologies), and 15 U Klenow fragment with 3'-to-5' exonuclease activity (NEB). Reactions were incubated at 37°C for 30 min, after which DNA was purified using a QIAquick MinElute Kit (Qiagen) and eluted in 19 µl of elution buffer according to the manufacturer’s instructions.

- 1. Illumina® adaptor ligation

Ligation reactions (50 µl volumes) involved incubation of 19 µl of blunt-ended aurochs DNA extracts (with a 3'-dATP overhang) in 1× DNA ligase buffer (NEB), 15 µM of each proprietary Illumina^®^ GA paired-end genomic adaptors and 10 U T4 DNA ligase (Invitrogen/Life Technologies). Extracts were incubated at room temperature for 15 min, purified using QIAquick MinElute Kit (Qiagen) and eluted in 19 µl of elution buffer according to the manufacturer’s instructions.

- 1. PCR amplification of purified end-repaired, adaptor-ligated DNA templates

The nine individual paired-end Illumina^®^ GA libraries (C4_1-3_, C5_1-3_ and C6_1-3_) were generated via PCR amplification of the end-repaired adaptor-ligated DNA templates prior to sequencing. PCR amplifications (50 µl) comprised 19 μl of blunt end-repaired-linker-ligated aurochs DNA; 1× Phusion^®^ High-Fidelity DNA polymerase buffer (NEB); 1 μl of forward primer, 1 μl of reverse primer (Illumina^®^); 250 nM each dNTP (Invitrogen/Life Technologies); and 1 U Phusion^®^ High-Fidelity DNA polymerase (NEB). PCR amplification reactions consisted of an initial denaturation step of 98°C for 30 s, 12 cycles of 98°C for 10 s, 65°C for 30 s and 72°C for 30 s, followed by a final extension step of 72°C for 5 min. Non-template controls were included with all PCR amplification reactions.

PCR products were visualized following electrophoresis on a 1.5% agarose gel stained with ethidium bromide (0.5 µg/ml; Invitrogen/Life Technologies). Examination of the PCR products indicated that the majority of the aurochs DNA inserts within the Illumina^®^ GA libraries were 40−60 bp in length. Individual libraries were subsequently combined according to their initial extract number (C4_1-3_, C5_1-3_ and C6_1-3_) to form three final pooled paired-end libraries labelled C4, C5 and C6, respectively (**Figure S1**). Pooled libraries were purified using a QIAquick PCR Purification Kit (Qiagen) and eluted in 30 µl elution buffer according to the manufacturer’s instructions. Purified libraries were quantified using a Qubit™ fluorometer (Invitrogen/Life Technologies) and a Quant-iT™ double-stranded DNA High-Sensitivity Assay Kit (Invitrogen/Life Technologies). The final molar concentration of each of the three pooled libraries ranged between 0.25–0.31 µM based on a median library fragment size of 250 bp (40.6–50.3 ng/µl).

- 1. High-throughput sequencing of aurochs paired-end libraries C4-C6

For the paired-end libraries C4, C5 and C6, cluster generation and sequencing was performed on an Illumina^®^ Cluster Station and a GA IIx sequencer according to the manufacturer’s instructions. Libraries were sequenced using both single-read and paired-end sequencing. Three flow cells were used for single-read sequencing, with read lengths of 36 bp, 42 bp and 70 bp. Three additional flow cells were used for paired-end sequencing, all with a read length of 42 bp. Analysis was performed using the standard Illumina^®^ GA pipeline. Intensity files generated by the IPAR server software were base called using Bustard base calling software package (Illumina^®^). The first flow cell was processed using the Illumina^®^ GA pipeline version 1.0. All subsequent flow cells were processed using Illumina^®^ GA pipeline version 1.3. This sequencing of paired-end libraries was performed at the Animal Genomics and Improvement Laboratory, Agricultural Research Service, USDA, Beltsville, MD, USA. Additional single-end sequencing of one of the paired-end libraries was performed at the Conway Institute of Biomolecular and Biomedical Research, University College Dublin, Ireland, using the method previously described for libraries C1 to C3 [9].

1. Targeted enrichment of aurochs nuclear genes

The two single-read Illumina^®^ GA sequencing libraries, C1 and C2, were used for targeted enrichment of bovine genes using the Agilent SureSelect oligonucleotide hybridisation solution-based capture system (Agilent Technologies). The complete list of targeted genes is provided in **Table S17**. The synthetic RNA baits were designed to capture exonic sequences for all genes based on the Btau 4.0 build of the *B. taurus* genome assembly. In total, approximately 3.30 Mb of DNA sequence was targeted for enrichment.

Targeted enrichment of aurochs gene sequences was performed according to the manufacturer’s instructions using half-volume reactions for all protocol steps. In brief, biotinylated RNA sequence capture baits were incubated with ~145 ng of each of the C1 and C2 libraries for 24 h at 65°C. Captured material was removed from solution using streptavidin-coated M280 Dynabeads^®^ (Invitrogen/Life Technologies) and eluted in 12.5 µl SureSelect Elution Buffer (Agilent Technologies). The eluate solution was subsequently neutralised with 12.5 µl SureSelect Neutralisation Buffer and eluted in 10 µl Elution Buffer following purification using a MinElute kit (Qiagen), according to the manufacturer’s instructions.

The enriched C1 and C2 libraries were then PCR amplified in 20 µl final volume reactions that included 3 µl enriched library DNA template; 0.5 nM final concentration of the proprietary Illumina PCR 1.1 and 2.1 primers; 200 nM final concentration of each dNTP; 1× Phusion^®^ High-Fidelity DNA Polymerase buffer (NEB); and 0.4 U Phusion^®^ High-Fidelity DNA Polymerase (NEB). PCR amplifications consisted of an initial denaturation step of 98°C for 30 s, 40 cycles of 98°C for 10 s, 65°C for 30 s and 72°C for 30 s, followed by a final extension step of 72°C for 5 min. Non-template controls were included with all PCR amplifications.

PCR products were visualized following electrophoresis on a 1.5% agarose gel stained with ethidium bromide (0.5 µg/ml; Invitrogen/Life Technologies) and eluted in 20 µl Elution Buffer following purification using a Qiagen MinElute kit, according to the manufacturer’s instructions. The PCR-amplified SureSelect-enriched C1 and C2 libraries were quantified using a Qubit™ fluorometer and a Quant-iT™ double-stranded DNA High-Sensitivity Assay Kit (Invitrogen/Life Technologies). The final molar concentrations for each of the SureSelect-enriched library were 0.28 μM (C1) and 0.21 μM (C2). The SureSelect-enriched C1 and C2 libraries were used for single-read sequencing, with a read length of 42 bp, on an Illumina^®^ GA IIx sequencer located in the Conway Institute of Biomolecular and Biomedical Research, University College Dublin, Ireland.

1. Bovine genome template sequence

The bovine genome template used for all analyses was version UMD3.1, released November 2009. Sequences for all reference genome chromosomes and unmapped scaffolds were downloaded from the UCSC genome browser website (<http://genome.ucsc.edu>). The GenBank haplogroup P complete mtDNA genome sequence (GenBank accession no. DQ124389) was used to replace the Hereford bovine genome UMD3.1 reference mtDNA sequence. It is important to acknowledge that mapping CPC98 sequence reads to the UMD3.1 *B. taurus* reference genome could introduce alignment biases as outlined by Schubert and colleagues [13] that may influence downstream population genomics analyses.

1. Alignment of Illumina sequence reads to genome sequences

Reads were trimmed to remove Illumina^®^ adapter sequences from the 3' ends using the trimLRPatterns function from the Bioconductor package ShortRead [14]. Trimming was applied to reads for which one or more bases at the 3' terminus matched the 5' end of the sequencing adapter. Mismatch toleration was used to allow for sequencing error, with zero nucleotide mismatches permitted for adapter matches 4 bp or shorter, one mismatch for adapter matches of five to 8 bp, two mismatches for matches from nine to 20 bp and three mismatches for longer adapter matches. Reads containing at least 14 bp matching the 5' end of the adapter were also truncated, allowing up to two nucleotide mismatches. Unpaired reads shorter than 18 bp post-truncation were excluded from subsequent analyses. Low complexity reads containing ≥ 60% of a single nucleotide, and poorly-sequenced reads containing ≥ 10% Ns (ambiguous nucleotides) were also excluded.

Distributions of nucleotide composition were determined for each base position across all sequence reads for each flow cell lane in the forward (5' → 3') and reverse (3' → 5') directions using a custom perl script. Reads were trimmed to remove any systematic bias detected: all C1 and C2 library reads were truncated by 1 bp at the 5' end and by 2 bp at the 3' end, all C3 library reads by 1 bp at both 5' and 3' ends, and all C4, C5 and C6 library reads by 1 bp at the 3' end. Sequence quality was assessed using the utility FastQC [version 0.9.0] ([www.bioinformatics.bbsrc.ac.uk/projects/fastqc](http://www.bioinformatics.bbsrc.ac.uk/projects/fastqc)) to evaluate sequence quality across reads and identify over-represented sequences remaining after trimming.

Trimmed Illumina GA reads were aligned to the UMD3.1 bovine genome sequence using the software package BWA (Burrows-Wheeler Aligner), version 0.6.1-r104 [15]. Alignments were performed separately for each flow cell lane. Two gaps were permitted per read (bwa aln ‘-o’ option), the seed length was set to 16 bp (bwa aln ‘-l’ option) and the right-trim parameter was set to 15 (bwa aln ‘-q’ option). Reads aligning equally well to multiple locations were assigned mapping quality scores of zero, and were filtered out prior to downstream analyses (bwa samse / sampe ‘-n’ option). For paired-end reads, maximum insert size was set to 50 kb (bwa sampe ‘-a’ option). All other parameters were left as default.

After alignment, reads that failed to align were filtered out and reads that successfully aligned were realigned to account for known SNPs and indels using the Broad Institute GATK software package [version v1.4-8-g63b7a70] according to the GATK best practice recommendations [16, 17] and with polymorphism data from bovine dbSNP Build 133 ([www.ncbi.nlm.nih.gov/snp](http://www.ncbi.nlm.nih.gov/snp)). Mate positions for paired end reads were corrected post-realignment using the picard tools (version 1.61) fixmate utility (<http://broadinstitute.github.io/picard>) and duplicate reads were removed using the picard markduplicates tool. Base alignment quality recalibration was performed using the GATK software package, with polymorphism data from bovine dbSNP version 133.

Alignment files were merged to give one file for each sequencing library, and a second round of duplicate removal, SNP-indel sensitive read realignment and read mate position fixing was performed as described above. The final individual library files were merged to give a single alignment file incorporating all uniquely-aligned non-duplicate reads.

1. Assessment of aurochs genome sequence authenticity

The complete CPC98 mtDNA sequence was used to estimate the amount of modern bovine DNA contamination as described by us previously using a smaller data set of sequence reads from the CPC98 mitochondrial genome [9].

We also estimated potential nuclear DNA contamination by taking advantage of the hemizygous X chromosome in the male CPC98 specimen. To do this, X chromosome SNPs were extracted from the region outside the pseudoautosomal region (PAR) of the X chromosome. These SNPs were selected according to the following criterion: 10 or more *B. indicus* and 15 or more European *B. taurus* animals have one or more read at the SNP position and the two clades are fixed for different alleles (*i.e.* the reference allele observed in taurine males and an alternate allele observed in zebu males). This procedure generated 1,530 non-PAR SNPs. The SAMtools [18] mpileup command was then used with the CPC98 BAM alignment file to obtain CPC98 reads covering these 1,530 SNP positions. Two levels of filtering were used: a) map quality phred score ≥ 20 and base quality score ≥ 13 (default); and b) map quality phred score ≥ 20 and base quality score ≥ 20 (stricter threshold to exclude reads that may be sequenced incorrectly). Following this, for SNPs with a read depth of ≥ 5 in the CPC98 specimen, genotypes were called as reference (*i.e.* European taurine), alternate (*i.e.* zebu) or heterozygous based on a simple majority allele count (heterozygous if tied). An upper estimate of European taurine nuclear DNA contamination was obtained through examination of non-PAR SNP positions where CPC98 exhibited the ‘zebu’ genotype and counting of ‘zebu’ alleles versus the subset of reads that conflict with the majority genotype (*i.e.* those that have the European taurine allele). An upper estimate of zebu nuclear DNA contamination was estimated in the same fashion using non-PAR SNP positions in CPC98 with the European taurine genotype and the subset of reads that have the zebu allele.

A mapDamage 2.0 analysis was completed using a subset of 1% (4.7 million) of the uniquely mapped reads from the final CPC98 alignment. The mapDamage 2.0 program was run using default settings, apart for the subsetting, and the output graphs were visually inspected (see **Figure S4**). An increase of C→T and G→A transitions were observed at the 5' and 3' ends of reads, respectively, a feature characteristic of aDNA. However this increase is reduced in comparison to other bones of a similar age; for example, those recently reported by Gamba and colleagues [19]. This result is likely due to an artefact caused by the Illumina^®^ AT-overhang library preparation and the Phusion polymerase used. Similar damage patterns to those seen in CPC98 were demonstrated in a Late Pleistocene (circa 11,480 YBP) Hippidion bone (*Hippidion saldiasi*) by Seguin-Orlando and co-workers using an AT-overhang library protocol in a comparative study of DNA damage patterns for different library preparation methods [20]. Nucleotide frequencies of the aligned reference sequence (see **Figure S4**) also showed the aDNA characteristic enrichment in purines at genomic positions preceding read starts is evident for CPC98 sequence reads.

1. Population genomics, phylogenetics and functional analyses
   1. Identification of SNP and indel variation

GATK (version v1.4-8-g63b7a70) [17] was used to call variants, recalibrate variant quality using a subset of variants called at high quality, and to filter the resulting variant list.

Initial variant calling was performed using the GATK UnifiedGenotyper tool, calling both SNPs and indels with phred quality > 40, and further outputting low quality variants with phred quality between 10 and 40. This initial pass identified 5,047,183 potential SNPs and 185,552 potential indels, which were subsequently filtered to retain only variants called with high confidence. For quality recalibration in the absence of an independent training set of variants, training sets were filtered from the CPC98 data itself by selecting SNPs and indels with very high phred quality (216,907 SNPs and 33,391 indels with phred quality ≥ 300).

The GATK VariantRecalibrator tool was used separately for SNPs and for indels using the full list of SNPs/indels as the test set and the high quality filtered SNPs/indels as the training set. The Gaussian models of variant quality generated by VariantRecalibrator were applied to the SNP and indel data sets using the GATK ApplyRecalibration tool, using a truth sensitivity threshold of 95% of SNPs from the SNP training data set and of 90% of indels from the indel training data set.

Additional filtering was applied using the GATK VariantFiltration tool to remove any remaining variants with low phred scores (< 50), low quality-by-depth scores (QD < 1.5), high strand bias (FS > 20), low read depth (DP < 5), and having a high proportion of reads with map quality of zero (MQ0 ≥ 4 and MQ0/DP < 0.1) and low recalibrated VQSLOD scores (vqslod < 2).

Recalibration and filtering of the initial variant list yielded a total of 2,009,261 confidently called SNPs and 104,655 confidently called indels. Of the 2,009,261 high confidence SNPs, 99.9% had a read depth between 5 and 31 and of the 104,655 high confidence indels, 99.9% had a read depth between 5 and 24.

- 1. Comparative sequence and SNP data from shallow cattle genome sequencing

Shallow Illumina sequencing was performed using a panel of 81 individual cattle samples from 11 different *B. taurus* and *B. indicus* breeds to give a composite sequence coverage of 128.4×, as follows: Angus (*n* = 10 animals, 15.5× total coverage); Holstein (*n* = 10 + 1, 12.0× + 9.9×); Jersey (*n* = 10, 10.8×); Limousin (*n* = 10, 16.0×); Romanola (*n* = 3, 3.7×); Fleckvieh (*n* = 1, 10.0×); Brahman (*n* = 10, 13.9×); Gir (*n* = 6, 2.6×); Nelore (*n* = 10, 14.0×); Sahiwal pooled (*n* = 5, 10.0×); N'Dama pooled (*n* = 5, 10.0×).

The Illumina^®^ GA IIx sequencing platform was used to generate 75 bp paired-end reads and sequence data was mapped to the Btau UMD 3.1 genome assembly using BWA [15]. The resulting mapped read files were split by chromosome and then all samples were merged by chromosome. For each chromosome, pileups were generated using SAMtools [18]. SNPs were then detected from the alignment pileups using a custom in-house SNP caller (Schroeder, S., unpublished). The in-house SNP caller associated each base-call in the pileup output with the animal source and breed. Based on this information, a SNP had to meet the following criteria: 1) only covering reads that have phred quality ≥ 15 at the SNP location counted; 2) at least 3 reads covering the SNP; 3) ≥ 1 read aligned from each direction; and 4) observed in at least two animals. Additional SNPs were also called, flagged and filtered into lower-quality groups with less stringent criteria (forward or reverse read orientation and reduced numbers of cumulative reads and sample observations). The most stringently filtered group consisted of 18,794,430 SNPs and 1,480,183 indels.

- 1. Assembly of BovineSNP50 SNP reference data set

Nucleotide calls were obtained for the CPC98 aurochs at nucleotide positions represented on the Illumina BovineSNP50 v2 BeadChip [6], using flanking sequence obtained from the dbSNP database to convert data to Illumina top strand encoding. For the current study, we genotyped 30 Kerry cattle samples from Ireland (previously collected by us [21]) using the Illumina BovineSNP50 v2 BeadChip. Additional BovineSNP50 data from a further 1,524 modern cattle and yak (*Bos grunniens*) samples were obtained from previously published work [2-7]. All data were converted, where necessary, to top strand encoding, and SNP identifiers and nucleotide positions were updated to bovine genome build UMD3.1. Data were imported into the plink software package [22] for processing. Duplicate samples were identified using pairwise identity by state calculation, and merged where found. A custom perl script was used to identify samples that were potentially parent/offspring pairs and one member of each such pair was removed at random. SNPs that were duplicated, were identified as incorrectly mapped [23], deviated from HWE in any breed (FDR controlled *P* ≤ 0.05), were untyped in over 10% of animals, or were covered by fewer than 10 reads in the aurochs CPC98 specimen were excluded. Individuals typed at fewer than 90% of remaining SNPs were excluded, giving a data set comprising 1,296 animals and 15,498 autosomal SNPs. It is important to note that the majority (73%) of SNPs removed to generate the final data set of 15,498 SNPs were excluded on the basis of the read depth criterion used for the CPC98 whole-genome sequencing data (≥10 reads covering each SNP).

This data set was further filtered such that the maximum number of samples representing any breed or population was limited to 40 animals by random removal of samples. This yielded a final data set comprising 1,226 animals representing a single aurochs specimen and samples from 73 modern populations (**Table S6**). Mean observed heterozygosity for the dataset is 0.275 ± 0.048, and observed heterozygosity for the CPC98 sample is 0.250. This figure for aurochs is lower than that for European *B. taurus* animals (0.310 ± 0.015), but higher than for *B. indicus* (0.171 ± 0.016) and West African *B. taurus* (0.213 ± 0.021), indicating no bias towards heterozygote excess or deficiency in the CPC98 BovineSNP50 SNP position genotype calls.

- 1. STRUCTURE analysis

For computational efficiency, the 15,498 autosomal SNPs in the BovineSNP50 SNP reference data were reduced by half through exclusion of every alternate SNP locus, giving 7,749 SNP loci that were used for STRUCTURE analysis. When grouping animals into populations, the N’Dama from Burkina Faso were considered as two distinct populations as per Gautier and colleagues [5]. A total of 73 modern populations—mostly representing distinct breeds—were used for analysis. For all datasets generated, STRUCTURE analysis parameters were left at their defaults except for burn-in replicates (set to 20,000) and further repetitions (set to 100,000). A range of assumed populations *K* from 2 to 24 were explored [24]. Genotype input files for STRUCTURE were produced from the SNP data via plink using the --recode-structure flag. Bar charts of the STRUCTURE output were then drawn using the DISTRUCT program [25] with standard settings. To aid visualisation, the STRUCTURE output data for the single CPC98 aurochs specimen was expanded to a width corresponding to 20 samples.

- 1. Phylogenetic tree construction

The SNPhylo software package [26] was used to construct a maximum likelihood (ML) phylogenetic tree (**Figure 2**). For this, the BovineSNP50 reference SNP data set (15,498 SNPs for 1,296 samples) was first formatted to a SNPhylo input file using a custom Python script. The data set was then filtered using a linkage disequilibrium threshold [LD] (*r*^2^ ≤ 0.5) implemented in SNPhylo, which generated a subset of 10,923 high-quality SNPs. This tree was also constructed with a reduced panel of 278 representative cattle [a maximum of five randomly sampled animals per breed from 59 modern breeds excluding the taurine/zebu or African taurine/European taurine crossbred populations] (**Table S6**). The evolutionary distance metric used for this tree was generated from the *p*-distance matrix for all samples included in the analysis. This *p*-distance metric represented the proportion of SNP genotypes that differed for any two-sample comparison using all SNP data; this calculation was performed for all samples in the data set and the resulting *p*-distance values in the matrix were used as an evolutionary distance for phylogenetic tree construction [26]. Bootstrap support values for the phylogeny were generated using 100 pseudo-replicates of the SNP genotypes and a maximum likelihood tree was generated, visualized and annotated using the FigTree program ([http://tree.bio.ed.ac.uk/software/figtree](http://tree.bio.ed.ac.uk/software/figtree/)).

An additional ML tree (**Figure S5**) with Bayesian branch support values was produced using the PhyML software package [27] and the same subset of 10,923 high-quality BovineSNP50 SNPs for the reduced panel of 278 cattle samples. The evolutionary distance metric used by the PhyML package is based on the model of DNA sequence evolution described by Gascuel [1].

The TreeMix software package (version 1.12) [28] was also used for phylogenetic analyses and to investigate interpopulation migration using the ancestry graphs generated by this program. Using genome-wide SNP data, TreeMix initially models genetic drift to infer relationships among sampled populations and generate a maximum likelihood tree. The covariance structure represented by this tree is then compared to the observed covariance and admixture is assumed when populations are more closely related than modelled by the tree. The program then adds a link (edge) between the two populations or population groups, which generates a phylogenetic network that models the timing and likely source and recipient populations for migration events. The migration edges are colour-coded according to migration weight, *i.e.*, the ancestry proportion received from the source population.

The BovineSNP50 reference SNP data set (15,498 SNPs) for 50 taurine cattle breeds from Europe and the Americas grouped into regional population groups was used with the single CPC98 aurochs specimen for TreeMix analysis. The Brahman zebu breed was used as an outgroup. Standard software settings for TreeMix were used with 15,498 SNPs grouped into blocks of 50 (-k 50) to account for linkage disequilibrium and the sample size correction (-noss) turned off as recommended in the TreeMix manual [29]. Migration edges were added sequentially to the population phylogenetic network up to 10 individual migration edges (-m 1 to –m 10). **Figures S7**, **S9** and **Figure 6** show the phylogenies and phylogenetic network resulting from these analyses. In addition, the three population test for admixture described by Reich and colleagues [30] and implemented with the threepop program in the TreeMix software package was used for the CPC98 aurochs and the eight regional population groups with blocks of 50 SNPs as described above.

- 1. Principal components analysis

Principal components for plotting were produced using SMARTPCA within the EIGENSOFT 4.2 software package [31]. Following Skoglund and colleagues [32], one SNP was removed from each pair of SNPs with an *r*^2^ value greater than 0.2. All other parameters were left at their defaults. These settings resulted in a dataset of 12,874 high-quality filtered BovineSNP50 SNPs and 1,226 animals for EIGENSOFT PCA.

- 1. ABBA/BABA test for genomic admixture

The ABBA/BABA (*D* statistic) test [33] was performed using the primary set of 15,498 autosomal SNPs to identify breeds showing significant admixture with British aurochs. ABBA/BABA tests were run using a population-level test, with significance assessed using a weighted block jackknife as described by Green and colleagues [34]. The ABBA/BABA test uses four populations: an outgroup species that diverged from the other three early in their history (O, *B. grunniens* − the yak); an archaic population, the putative source of the gene flow (A, the CPC98 aurochs); and two closely related test populations (P1 and P2). The tree topology is therefore (((P1, P2), A), O) and the test is whether there has been gene flow between A and P1, or conversely between A and P2. The test compares populations P1 and P2 to detect any excess of derived alleles due to admixture with the archaic population (A). Requirements for the test are that the archaic population (A) must feature a derived allele that is absent in the outgroup O (*B. grunniens* − the yak) and that this derived allele must also be present in at least one of the two test populations P1 and P2. For populations P1 and P2, all possible pairwise combinations of the 60 taurine populations (1,770) were used, with the Burkina Faso N’Dama considered as two distinct populations as described by Gautier and colleagues [5].

The block jackknife determines the variance of the test statistic by dividing the genome into *n* blocks of fixed physical length and performing the test while excluding each block in turn. It is recommended that the length of the blocks exceeds the length of linkage disequilibrium (LD) blocks in the test populations [34]. Consequently, block lengths of 20 Mb were used here and also a weighting scheme was used for the block jackknife to take account of variable numbers of SNPs in different blocks.

A geographical representation of individual European breed *D* statistics (**Table S7**) was generated as a contour plot (**Figure 5**) using the ArcMap component of the ArcGIS software suite version 10.1 (ESRI Ireland, Dublin, Ireland) with the means of the seven pairwise comparisons to the West African taurine populations (Baoulé, Lagune, Somba and four separate N’Dama populations [Burkina Faso ×2, Gambia, Guinea]) for each European taurine population. The West African taurine populations were used under the assumption that they have minimal or no European aurochs admixture. Also, there were seven discrete West African taurine populations with relatively large sample sizes (mean *n* = 20.8) compared to the five discrete Asian/American zebu populations with the least taurine admixture (ZBRA, ZGIR, ZGUZ, ZNEL, ZSAH) [mean *n* = 15.6]. In addition, the aurochs is not an outgroup to the *B. indicus* clade, which violates an assumption of the ABBA/BABA method [33].

- 1. Variant effect predictor analyses applied to CPC98 and modern animal SNPs and indels

SNPs and indels distinguishing the CPC98 genome sequence from the *B. taurus* reference genome were evaluated for potential functional effects using the Ensembl Variant Effect Predictor (VEP) tool (build 78) [35]. The set of high confidence SNPs and indels in **Table 1** were used as input, and their locations were determined relative to coding and regulatory sequences in Ensembl genome build 78 based on the *B. taurus* reference genome build UMD3.1. Results are shown in **Additional File 2**.

We also searched for potentially functionally important SNPs and indels that apparently arose in modern European *B. taurus* after the divergence from Eurasian aurochs and subsequently increased in frequency to fixation or near fixation, possibly as a result of selection. The input data set comprised 6,805,463 high confidence SNPs and 83,218 high confidence indels obtained from shallow sequencing of modern cattle for which the CPC98 genotype could also be called at high confidence. We filtered for biallelic variants with alleles *A* and *B* for which the frequency of allele *A* in genotyped modern *B. indicus* animals (*n* ≥ 5) was at least 95% and such that the CPC98 specimen was homozygous for allele *A*, while the frequency of allele *B* was at least 95% in European *B. taurus* animals (*n* ≥ 5). The filtered data set included 2,306 SNPs and 74 indels. The Ensembl VEP tool was used to locate these variants within genes in the UMD3.1 reference genome (Ensembl build 78) and thereby identify any SNPs and indels that might have an effect on gene function.

Intronic and intergenic variants were excluded, and only variants within coding sequence, 5' and 3' UTRs and 5 kb up- and downstream flanking sequences of genes were retained. 263 such variants were observed, with a total of 321 associated variant effects reported. The 263 variants were observed to be associated with 193 different bovine genes, which were used for subsequent analyses using IPA. **Table S9** shows predicted effects for these 263 SNPs and indels potentially selected post-domestication in European *B. taurus* cattle. In addition, **Table S10** shows details for eight missense SNPs potentially selected post-domestication in European *B. taurus* cattle.

- 1. HKA test for detection of selective sweeps

The Hudson, Kreitman and Aguadé (HKA) test of neutral molecular evolution is based on the ratio of allelic diversity within an ingroup population/species to the evolutionary distance to an outgroup at a particular locus [36]. In the absence of selective sweeps or balancing selection, this parameter should be constant throughout the genome. However, selective sweeps, which cause a local genomic reduction in effective population size, will reduce the ingroup diversity, while balancing selection may increase allelic diversity.

For the present study, we applied a modified genome-wide multilocus partial HKA test method (HKAdirect) described by Esteve-Codina and colleagues and used to detect selective sweeps in various Iberian pig populations [37]. This analysis used 6.89 million SNPs generated from comparison of 128.4× combined coverage whole genome re-sequencing of 11 taurine and zebu breeds, for which the CPC98 aurochs genome provided high-quality allele calls. The genome was partitioned into 50 kb, 100 kb or 200 kb overlapping windows, which were moved by 10% of the window length every iteration. For the HKAdirect test, only European taurine animals were used as the ingroup and one allele was sampled from each animal from either *n* = 20 or *n* = 25, with SNPs represented in fewer than *n* animals excluded. For the outgroup, one allele was sampled from the CPC98 aurochs genome and SNPs with information for fewer than *n* animals or no information from CPC98 were excluded from the analysis. For each window, the number of segregating sites was obtained within the ingroup and the mean allele-sharing pairwise diversity between the CPC98 aurochs and the ingroup samples was calculated. The partial HKA test was applied to each window and windows with a test statistic significance value ≤ 0.05 were identified for all six analyses. In addition, where windows overlapped, they were incorporated into extended regions. Genes overlapping the detected regions were tabulated and subject to downstream systems analyses using the Ingenuity^®^ Systems Pathway Analysis Knowledge Base (IPA; [www.ingenuity.com](http://www.ingenuity.com)).

- 1. Identification and ranking of miRNA targets in cattle and aurochs

Aurochs miRNAs were identified by mapping the cattle miRNA coordinates onto whole genome sequence alignments of the cattle genome (UMD3.1) and the aurochs consensus sequence. To identify mammalian miRNAs in the genome of aurochs that are not annotated in the bovine genome, the mature sequences of the mammalian miRNAs (across all available species) were used in conjunction with the aurochs consensus sequence and putative miRNA genes were identified using MapMi [38]. The putative aurochs miRNA genes were compared to the closest mammalian homolog as defined by MapMi scores; these putative miRNA genes were then searched for in the aligned bovine reference genome sequence.

To identify miRNA binding sites in target genes, the 3' UTRs sequences and their coordinates for the bovine genome were retrieved from Ensembl using Biomart (UMD3.1) [39, 40]. The aurochs-predicted 3' UTR sequences were retrieved from the assembled aurochs genome using the coordinates associated with each bovine 3' UTR. Integration of 3' UTR sequences from cattle and aurochs into multiple sequence alignments was used for Targetscan miRNA binding site prediction. To identify miRNA target binding sites within cattle and aurochs 3' UTRs, Targetscan was used [41]. Targetscan has been shown to be more accurate than other programs [42, 43], as it takes into account the extent of evolutionary conservation of a predicted miRNA binding site, providing higher confidence scores[44].

TargetScan is divided into two main Perl scripts, targetscan_60.pl and targetscan_60_context_scores.pl. The script targetscan_60.pl takes as its input the multiple sequence alignments of the 3' UTRs and the list of miRNA seed regions for *B. taurus* miRNAs and returns the identified binding sites. Any domestic cattle and aurochs seed regions identified that contain ambiguous nucleotides in the aurochs 3' UTR were removed from the analysis. Any seed sites overlapping with an already identified seed site for the same miRNA were also removed. The second script targetscan_60_context_scores.pl provides a score for each site. The Targetscan context score corresponds to a prediction, based on experimental miRNA repression expressed in log-ratios [45, 46]. To parse the results, a custom Python script was used, which returned the results as two files, one for the genes with polymorphic binding sites between aurochs and domestic cattle and a second one with common binding sites. For miRNAs with multiple binding sites, the score for each gene/miRNA pair, was made by summing the individual context scores calculated by TargetScan for each site. To rank genes, we calculated an additional score, which we termed the *Score-Site ratio* (*SSr*) to allow us to compare the extent to which miRNA binding sites were polymorphic between domestic cattle and aurochs, compared to those which were common between them. The *SSr* was calculated by summing all context scores, for each predicted miRNA binding site across each 3' UTR and multiplied by the ratio of the number of polymorphic sites over the number of the common sites between cattle and aurochs:

$$SSr= \sum_{1}^{n} {score}_{i}\times\frac{psn}{csn}$$

where: *n* = the number of miRNA binding sites identified for one gene UTR; *score*_i_ = the score for each predicted site; *psn* = the number of polymorphic miRNA binding sites for one gene; and *csn* = the number of common miRNA binding sites for one gene.

The *SSr* score was used to rank the genes with miRNA binding site polymorphisms between aurochs and domestic cattle and identify which genes have been the most affected by the evolution of miRNA site polymorphisms between aurochs and domestic cattle.

IPA was used to identify biological pathways that are most enriched for genes with binding site variation in the 3' UTRs between domestic cattle and aurochs. The bovine genes targeted by a polymorphic miRNA when compared with aurochs, were used as inputs for IPA. A score was then attributed to each significant pathway, which corresponded to the sum of the *SSr* score of each gene involved in the pathway. The pathways were then ranked based on these scores to obtain a list of the top pathways for polymorphism effects in miRNA binding sites between domestic cattle and aurochs.

References

1. Gascuel O: **BIONJ: an improved version of the NJ algorithm based on a simple model of sequence data.** *Mol Biol Evol* 1997, **14:**685-695.

2. Gautier M, Laloe D, Moazami-Goudarzi K: **Insights into the genetic history of French cattle from dense SNP data on 47 worldwide breeds.** *PLoS ONE* 2010, **5**.

3. Gibbs RA, Taylor JF, Van Tassell CP, Barendse W, Eversole KA, Gill CA, Green RD, Hamernik DL, Kappes SM, Lien S, et al: **Genome-wide survey of SNP variation uncovers the genetic structure of cattle breeds.** *Science* 2009, **324:**528-532.

4. Decker JE, Pires JC, Conant GC, McKay SD, Heaton MP, Chen K, Cooper A, Vilkki J, Seabury CM, Caetano AR, et al: **Resolving the evolution of extant and extinct ruminants with high-throughput phylogenomics.** *Proc Natl Acad Sci U S A* 2009, **106:**18644-18649.

5. Gautier M, Flori L, Riebler A, Jaffrezic F, Laloe D, Gut I, Moazami-Goudarzi K, Foulley JL: **A whole genome Bayesian scan for adaptive genetic divergence in West African cattle.** *BMC Genomics* 2009, **10:**550.

6. Matukumalli LK, Lawley CT, Schnabel RD, Taylor JF, Allan MF, Heaton MP, O'Connell J, Moore SS, Smith TP, Sonstegard TS, Van Tassell CP: **Development and characterization of a high density SNP genotyping assay for cattle.** *PLoS ONE* 2009, **4:**e5350.

7. Flori L, Fritz S, Jaffrezic F, Boussaha M, Gut I, Heath S, Foulley JL, Gautier M: **The genome response to artificial selection: a case study in dairy cattle.** *PLoS ONE* 2009, **4:**e6595.

8. Benjamini Y, Hochberg Y: **Controlling the false discovery rate - a practical and powerful approach to multiple testing.** *J R Stat Soc Ser B Method* 1995, **57:**289-300.

9. Edwards CJ, Magee DA, Park SD, McGettigan PA, Lohan AJ, Murphy A, Finlay EK, Shapiro B, Chamberlain AT, Richards MB, et al: **A complete mitochondrial genome sequence from a mesolithic wild aurochs (*Bos primigenius*).** *PLoS ONE* 2010, **5:**e9255.

10. Edwards CJ, Gaillard C, Bradley DG, MacHugh DE: **Y-specific microsatellite polymorphisms in a range of bovid species.** *Anim Genet* 2000, **31:**127-130.

11. MacHugh DE, Edwards CJ, Bailey JF, Bancroft DR, Bradley DG: **The extraction and analysis of ancient DNA from bone: a survey of current methodologies.** *Ancient Biomolecules* 2000, **3:**81–102.

12. Troy CS, MacHugh DE, Bailey JF, Magee DA, Loftus RT, Cunningham P, Chamberlain AT, Sykes BC, Bradley DG: **Genetic evidence for Near-Eastern origins of European cattle.** *Nature* 2001, **410:**1088-1091.

13. Schubert M, Jonsson H, Chang D, Der Sarkissian C, Ermini L, Ginolhac A, Albrechtsen A, Dupanloup I, Foucal A, Petersen B, et al: **Prehistoric genomes reveal the genetic foundation and cost of horse domestication.** *Proc Natl Acad Sci U S A* 2014, **111:**E5661-5669.

14. Morgan M, Anders S, Lawrence M, Aboyoun P, Pages H, Gentleman R: **ShortRead: a bioconductor package for input, quality assessment and exploration of high-throughput sequence data.** *Bioinformatics* 2009, **25:**2607-2608.

15. Li H, Durbin R: **Fast and accurate short read alignment with Burrows-Wheeler transform.** *Bioinformatics* 2009, **25:**1754-1760.

16. Van der Auwera GA, Carneiro MO, Hartl C, Poplin R, del Angel G, Levy-Moonshine A, Jordan T, Shakir K, Roazen D, Thibault J, et al: **From FastQ data to high-confidence variant calls: the Genome Analysis Toolkit best practices pipeline.** In *Current Protocols in Bioinformatics.* John Wiley & Sons, Inc.; 2013

17. DePristo MA, Banks E, Poplin R, Garimella KV, Maguire JR, Hartl C, Philippakis AA, del Angel G, Rivas MA, Hanna M, et al: **A framework for variation discovery and genotyping using next-generation DNA sequencing data.** *Nat Genet* 2011, **43:**491-498.

18. Li H, Handsaker B, Wysoker A, Fennell T, Ruan J, Homer N, Marth G, Abecasis G, Durbin R: **The Sequence Alignment/Map format and SAMtools.** *Bioinformatics* 2009, **25:**2078-2079.

19. Gamba C, Jones ER, Teasdale MD, McLaughlin RL, Gonzalez-Fortes G, Mattiangeli V, Domboroczki L, Kovari I, Pap I, Anders A, et al: **Genome flux and stasis in a five millennium transect of European prehistory.** *Nat Commun* 2014, **5:**5257.

20. Seguin-Orlando A, Schubert M, Clary J, Stagegaard J, Alberdi MT, Prado JL, Prieto A, Willerslev E, Orlando L: **Ligation bias in illumina next-generation DNA libraries: implications for sequencing ancient genomes.** *PLoS ONE* 2013, **8:**e78575.

21. MacHugh DE, Shriver MD, Loftus RT, Cunningham P, Bradley DG: **Microsatellite DNA variation and the evolution, domestication and phylogeography of taurine and zebu cattle (*Bos taurus* and *Bos indicus*).** *Genetics* 1997, **146:**1071-1086.

22. Purcell S, Neale B, Todd-Brown K, Thomas L, Ferreira MA, Bender D, Maller J, Sklar P, de Bakker PI, Daly MJ, Sham PC: **PLINK: a tool set for whole-genome association and population-based linkage analyses.** *Am J Hum Genet* 2007, **81:**559-575.

23. Fadista J, Bendixen C: **Genomic position mapping discrepancies of commercial SNP chips.** *PLoS ONE* 2012, **7:**e31025.

24. Pritchard JK, Stephens M, Donnelly P: **Inference of population structure using multilocus genotype data.** *Genetics* 2000, **155:**945-959.

25. Rosenberg NA: **DISTRUCT: a program for the graphical display of population structure.** *Mol Ecol Notes* 2004, **4:**137-138.

26. Lee TH, Guo H, Wang X, Kim C, Paterson AH: **SNPhylo: a pipeline to construct a phylogenetic tree from huge SNP data.** *BMC Genomics* 2014, **15:**162.

27. Guindon S, Dufayard JF, Lefort V, Anisimova M, Hordijk W, Gascuel O: **New algorithms and methods to estimate maximum-likelihood phylogenies: assessing the performance of PhyML 3.0.** *Syst Biol* 2010, **59:**307-321.

28. Pickrell JK, Pritchard JK: **Inference of population splits and mixtures from genome-wide allele frequency data.** *PLoS Genet* 2012, **8:**e1002967.

29. Pickrell JK, Pritchard JK: **User Manual for TreeMix v1.1 (1st October 2012).** 2012**:**<https://bitbucket.org/nygcresearch/treemix>.

30. Reich D, Thangaraj K, Patterson N, Price AL, Singh L: **Reconstructing Indian population history.** *Nature* 2009, **461:**489-494.

31. Patterson N, Price AL, Reich D: **Population structure and eigenanalysis.** *PLoS Genet* 2006, **2:**e190.

32. Skoglund P, Malmstrom H, Raghavan M, Stora J, Hall P, Willerslev E, Gilbert MT, Gotherstrom A, Jakobsson M: **Origins and genetic legacy of Neolithic farmers and hunter-gatherers in Europe.** *Science* 2012, **336:**466-469.

33. Durand EY, Patterson N, Reich D, Slatkin M: **Testing for ancient admixture between closely related populations.** *Mol Biol Evol* 2011, **28:**2239-2252.

34. Green RE, Krause J, Briggs AW, Maricic T, Stenzel U, Kircher M, Patterson N, Li H, Zhai W, Fritz MH, et al: **A draft sequence of the Neandertal genome.** *Science* 2010, **328:**710-722.

35. McLaren W, Pritchard B, Rios D, Chen Y, Flicek P, Cunningham F: **Deriving the consequences of genomic variants with the Ensembl API and SNP Effect Predictor.** *Bioinformatics* 2010, **26:**2069-2070.

36. Hudson RR, Kreitman M, Aguade M: **A test of neutral molecular evolution based on nucleotide data.** *Genetics* 1987, **116:**153-159.

37. Esteve-Codina A, Paudel Y, Ferretti L, Raineri E, Megens HJ, Silio L, Rodriguez MC, Groenen MA, Ramos-Onsins SE, Perez-Enciso M: **Dissecting structural and nucleotide genome-wide variation in inbred Iberian pigs.** *BMC Genomics* 2013, **14:**148.

38. Guerra-Assuncao JA, Enright AJ: **MapMi: automated mapping of microRNA loci.** *BMC Bioinformatics* 2010, **11:**133.

39. Flicek P, Amode MR, Barrell D, Beal K, Brent S, Carvalho-Silva D, Clapham P, Coates G, Fairley S, Fitzgerald S, et al: **Ensembl 2012.** *Nucleic Acids Res* 2012, **40:**D84-90.

40. Kinsella RJ, Kahari A, Haider S, Zamora J, Proctor G, Spudich G, Almeida-King J, Staines D, Derwent P, Kerhornou A, et al: **Ensembl BioMarts: a hub for data retrieval across taxonomic space.** *Database (Oxford)* 2011, **2011:**bar030.

41. Lewis BP, Burge CB, Bartel DP: **Conserved seed pairing, often flanked by adenosines, indicates that thousands of human genes are microRNA targets.** *Cell* 2005, **120:**15-20.

42. Baek D, Villen J, Shin C, Camargo FD, Gygi SP, Bartel DP: **The impact of microRNAs on protein output.** *Nature* 2008, **455:**64-71.

43. Ulitsky I, Laurent LC, Shamir R: **Towards computational prediction of microRNA function and activity.** *Nucleic Acids Res* 2010, **38:**e160.

44. Friedman RC, Farh KK, Burge CB, Bartel DP: **Most mammalian mRNAs are conserved targets of microRNAs.** *Genome Res* 2009, **19:**92-105.

45. Garcia DM, Baek D, Shin C, Bell GW, Grimson A, Bartel DP: **Weak seed-pairing stability and high target-site abundance decrease the proficiency of lsy-6 and other microRNAs.** *Nat Struct Mol Biol* 2011, **18:**1139-1146.

46. Grimson A, Farh KK, Johnston WK, Garrett-Engele P, Lim LP, Bartel DP: **MicroRNA targeting specificity in mammals: determinants beyond seed pairing.** *Mol Cell* 2007, **27:**91-105.
